# A Mass Spectrometry Based Metabolite Profiling Workflow for Selecting Abundant Specific Markers and Their Structurally Related Multi-Component Signatures in Traditional Chinese Medicine Multi‐Herb Formulae

**DOI:** 10.3389/fphar.2020.578346

**Published:** 2020-12-03

**Authors:** Joëlle Houriet, Pierre-Marie Allard, Emerson Ferreira Queiroz, Laurence Marcourt, Arnaud Gaudry, Lennie Vallin, Songhua Li, Yu Lin, Ruwei Wang, Kenny Kuchta, Jean-Luc Wolfender

**Affiliations:** ^1^ School of Pharmaceutical Sciences, University of Geneva, Geneva, Switzerland; ^2^ Institute of Pharmaceutical Sciences of Western Switzerland, University of Geneva, Geneva, Switzerland; ^3^ Izumo Clinic, Izumo-shi, Japan; ^4^ Kunisawa Clinic, Gotsu-shi, Japan; ^5^ Zhejiang Provincial Key Laboratory of Traditional Chinese Medicine Pharmaceutical Technology, Hangzhou, China; ^6^ Forschungsstelle für Fernöstliche Medizin, Department of Vegetation Analysis and Phytodiversity, Albrecht von Haller Institute of Plant Sciences, Georg August University, Göttingen, Germany

**Keywords:** multi-herb formulae, Traditional Chinese Medicine, feature-based molecular network, quality control, multi-component signature, TCM, Mass spectrometry, Chemical markers

## Abstract

In Traditional Chinese Medicine (TCM), herbal preparations often consist of a mixture of herbs. Their quality control is challenging because every single herb contains hundreds of components (secondary metabolites). A typical 10 herb TCM formula was selected to develop an innovative strategy for its comprehensive chemical characterization and to study the specific contribution of each herb to the formula in an exploratory manner. Metabolite profiling of the TCM formula and the extract of each single herb were acquired with liquid chromatography coupled to high-resolution mass spectrometry for qualitative analyses, and to evaporative light scattering detection (ELSD) for semi-quantitative evaluation. The acquired data were organized as a feature-based molecular network (FBMN) which provided a comprehensive view of all types of secondary metabolites and their occurrence in the formula and all single herbs. These features were annotated by combining MS/MS-based *in silico* spectral match, manual evaluation of the structural consistency in the FBMN clusters, and taxonomy information. ELSD detection was used as a filter to select the most abundant features. At least one marker per herb was highlighted based on its specificity and abundance. A single large-scale fractionation from the enriched formula enabled the isolation and formal identification of most of them. The obtained markers allowed an improved annotation of associated features by manually propagating this information through the FBMN. These data were incorporated in the high-resolution metabolite profiling of the formula, which highlighted specific series of related components to each individual herb markers. These series of components, named multi-component signatures, may serve to improve the traceability of each herb in the formula. Altogether, the strategy provided highly informative compositional data of the TCM formula and detailed visualizations of the contribution of each herb by FBMN, filtered feature maps, and reconstituted chromatogram traces of all components linked to each specific marker. This comprehensive MS-based analytical workflow allowed a generic and unbiased selection of specific and abundant markers and the identification of multiple related sub-markers. This exploratory approach could serve as a starting point to develop more simple and targeted quality control methods with adapted marker specificity selection criteria to given TCM formula.

## Introduction

Multi-herb mixtures are used in many traditional medicines, such as Traditional Chinese Medicine (TCM), Japanese Kampo medicine, traditional European phytomedicine, but also in modern evidence-based herbal medicinal products (Abdel-Aziz et al., 2017). The quality assessment of such complex multi-component mixtures is challenging. The chemical quality markers are often secondary metabolites, which are frequently referred to as *components* in TCM literature (Liu et al., 2017), which is the terminology used in this study (for a glossary of terms, see Table 1). For individual herbs, modern pharmacopoeial monographs propose methods to check multiple chemical components both in terms of presence and relative quantification. Such methods, called *single standard to determine multi-components methods* (SSDMC), have the advantage of reducing the number of required standards, but remain limited to the verification of a single plant (Gao et al., 2009; Liang et al., 2013; Hou et al., 2019). These SSDMC methods address the limitations encountered with quality controls (QC) restricted to a single marker per herbal drug. These limitations have been highlighted by cases of adulteration and falsification, which concern all types of herbal preparations, including TCMs (Wang et al., 2018), and even more so, the huge market of food supplements (Abdel-Tawab, 2018). By being limited to a single marker or to the total amount of a given class of components, cases of falsification by adding the said marker have been reported, for example involving extracts of *Ginkgo biloba* leaves (Czigle et al., 2018). In the food supplement market, which is not submitted to specific QC, illegal additions of potentially dangerous pure substances were observed in herbal preparations (Skalicka-Wozniak et al., 2017; Kee et al., 2018).

**TABLE 1:** Terminology of terms used in this study (alphabetical order).

Anchor point	A node in a molecular network, whose formal identification is based on NMR after targeted isolation or by comparison of two independent and orthogonal data with a pure standard (i.e., retention time and *m*/*z*) (level 1 identification according to the Metabolomic Standard Initiative (MSI) (Sumner et al., 2007)
Bar chromatogram	A bar plot reconstituted from peak descriptors obtained after LC-HRMS data processing. The peak height is represented in bar form, as a function of retention time. The chromatographic peak is thus represented in a centroid way. This depiction allows simplifying the original chromatogram and focusing on the intensity and mean retention time of the peaks
Cluster specificity percentage	In a FBMN, average of the specificity percentages of the features grouped in a cluster for a given herb
Component	A chemical compound, also called chemical constituent, specialized or secondary metabolite, or natural product
Feature-based molecular network (FBMN)	Incorporation of LCMS features information (*m*/*z*, retention time, intensity) in the molecular networking of MS/MS spectra. It allows, among others, to distinguish isomers and to integrate relative quantitative information. In a FBMN, a feature is equivalent to a node (Nothias et al., 2020)
Formula	A combination of several TCM herbs (Pei et al., 2013)
Marker	A defined chemical compound for an herbal material utilized for control purposes (WHO, 2007)
Multi-component signature	A series of analogue components that are specific to one herb in a formula
Specificity percentage	Relative height intensity of one feature in a multi-herb formula. The peak height of one aligned feature in one herb is divided by the sum of the heights in all herbs

For multi-herb TCM mixture, named formulae (Table 1), developing holistic analytical methods that considers their complexity is an urgent goal (Guo et al., 2015; Yang et al., 2017; Hou et al., 2019). In a formula, checking several markers representing each herb is already challenging (Yao et al., 2016). Furthermore, the pharmacopoeial marker of an herb may be ubiquitous and also present in other herbs of the same formula (Wu et al., 2018). Thus, in this complex and multifactorial context, QC of multi-herb formulae should ideally verify the presence of each herb in a specific way, as well as the absence of falsification.

To give a rationale for developing appropriate methods of QC, an in-depth investigation of the chemical composition is one of the first conditions (Hou et al., 2019). For this, liquid chromatography coupled with mass spectrometry (LC-MS) is ideal for a comprehensive chemical characterization (Hou et al., 2019). In MS, the latest generations of mass spectrometers combine sensitive detection and high mass resolution power and are capable of acquiring high resolution spectra (HRMS) alternating with fragmentation spectra (HRMS/MS). In the field of TCM, these fragmentation spectra are today mainly acquired in the data dependent acquisition mode (DDA) (Hou et al., 2019).

One of the key limitations of plant metabolite profiling still resides in the unambiguous identification of all components. At present, in-depth chemical characterizations based on UHPLC-HRMS allow potential identifications, often referred to as annotation or dereplication (Wolfender et al., 2019). Interpreting untargeted HRMS data has greatly improved thanks to dedicated software and metabolomic approaches (Wolfender et al., 2019). Furthermore, organizing HRMS/MS data by molecular networking (MN) brought a novel dimension to metabolite annotation (Wang et al., 2016a). As a way of classifying structurally related chemicals, MN is seen as a tool of interest for TCMs chemical investigation (Hou et al., 2019) and has recently begun to be employed in TCM research to annotate single herb extracts (Pan et al., 2019; Wang et al., 2019; Wang et al., 2020b). Data processing step by open-source software, such as MZmine (Pluskal et al., 2010) prior to MN has enabled the generation of so-called *feature-based molecular networks* (FBMN) (Nothias et al., 2020) (Table 1). Such FBMN offer the following advantages compared to classic MN: accurate mass, semi-quantitative information conservation, and isomer separation. Additionally, *in silico* prediction of fragmentation pattern has increased the number of MS/MS spectra available for annotation, which led to the generation of large databases of theoretical spectra containing more than 200,000 spectra of secondary metabolites (Allard et al., 2016).

In this exploratory study, a 10 herbs TCM formula was selected to evaluate new approaches for in-depth chemical investigation from a QC perspective. This 10 herbs formula was chosen following an open-label clinical study that evaluated three new multi-herb formulae to treat atopic dermatitis (Li et al., 2013). This affection, also called atopic eczema, is a common inflammatory skin disorder and is treated in Western medicine by topical corticosteroids and emollients (Dempster et al., 2011). The oral formula generated the best clinical outcomes (Li et al., 2013), and was therefore selected for this study. It contains 10 herbs whose main pharmacological effects and TCM indications have previously been summarized (Li et al., 2013). In terms of chemical composition, these 10 herbs were previously described and several references, as well as the pharmacopoeial markers, are summarized in Table 2.

**TABLE 2:** TCM herbal drugs with their proportion in the formula before decoction, their botanical name and pharmacopoeial markers.

Code	Herbs	Family	Plant part	Proportion (w/w)	Marker compound	References
A	*Angelica sinensis* (Oliv.) Diels	Apiaceae	Roots	3.4%	Ligustilide (CPh)	Jin et al. (2012), Ma et al. (2015)
					*trans*-Ferulic acid (Ph.Eur.)	
C	*Chrysanthemum indicum* L.	Asteraceae	Flowers	6.9%	Chlorogenic acid (ChP)	Luyen et al. (2015)
					Luteolin (JP)	
G	*Glycyrrhiza uralensis* Fisch.	Fabaceae	Roots	3.4%	Uralsaponin (ChP, Ph.Eur.)	Wang et al. (2013)
I	*Isatis tinctoria* L.	Brassicaceae	Leaves	6.9%	Indican (ChP)	Mohn et al. (2009)
					Arginine (Ph.Eur.)	
O	*Oldenlandia diffusa* (Willd.) Roxb.	Rubiaceae	Entire plant	20.7%	Oleanolic acid (ChP)	Chen et al. (2016)
PO	*Reynoutria japonica* Houtt. (synonym *Polygonum cuspidatum* Siebold & Zucc.)	Polygonaceae	Rhizomes	10.3%	Emodin (ChP)	Peng et al. (2013)
PR	*Prunella vulgaris* L.	Lamiaceae	Fruiting tops	13.8%	Ursolic acid (ChP, Ph.Eur.)	Bai et al. (2016)
SC	*Scutellaria baicalensis* Georgi	Lamiaceae	Roots	6.9%	Baicalin (ChP, Ph.Eur.)	Karimov and Botirov (2017)
SM	*Smilax glabra* Roxb.	Smilacaceae	Rhizomes	20.7%	Octacosanol, astilbin (ChP)	Hua et al. (2018)
SO	*Sophora flavescens* Aiton	Fabaceae	Roots	6.9%	Matrine (ChP)	He et al. (2015)

ChP, Chinese Pharmacopoeia; Ph.Eur., European Pharmacopoeia; JP, Japanese Pharmacopoeia.

This study aimed at exploring the potential role of innovative UHPLC-HRMS/MS data processing tools for a comprehensive chemical characterization of such complex TCM formulae. The rationale was to select relevant specific markers for characterizing each herb in the formula from an unbiased data-driven manner. A workflow is proposed that combines state-of-the-art annotation of UHPLC-HRMS/MS metabolite profiling, FBMN, semi-quantitative Evaporative Light Scattering Detection (ELSD), as well as UV-PDA. ELSD belongs to the category of detectors which provides a proportional semi-quantitative response almost universal, but are not very sensitive (Lucena et al., 2007). In this study, ELSD was evaluated as a tool to focus on the main components from a semi-quantitative point of view. By combining these different detectors, a comprehensive view of the chemical composition of the formula was obtained by linking the detailed structural information provided by HRMS/MS and UV-PDA with the ELSD semi-quantitative information. From this comprehensive view, our workflow aimed to effectively evaluate the herb specificity of all features, to select specific and abundant markers, as well as to highlight series of related less abundant specific components of each herb marker in the formula for advanced traceability analysis. This exploratory study aims to assist in the development of routine QC methods well adapted to a given TCM formula.

## Results

### Study Overview

The formula selected for this study contains 10 various herbal drugs from eight botanical families: *Angelica sinensis* (Oliv.) Diels (Apiaceae)*, Chrysanthemum indicum* L. (Asteraceae), *Glycyrrhiza uralensis* Fisch (Fabaceae), *Isatis tinctoria* L. (Brassicaceae), *Oldenlandia diffusa* (Willd.) Roxb. (Rubiaceae)*, Reynoutria japonica* Houtt. (Polygonaceae), whose synonym, *Polygonum cuspidatum* Siebold & Zucc. was used in this study to stay in line with (Li et al., 2013)*, Prunella vulgaris* L. (Lamiaceae), *Scutellaria baicalensis* Georgi (Lamiaceae), *Smilax glabra* Roxb. (Smilacaceae) and *Sophora flavescens* Aiton (Fabaceae) (Table 2). A wide variety of different chemical classes has been described for these 10 herbal drugs, ranging from the usual flavonoids to alkaloids (*S. flavescens* (He et al., 2015), *I. tinctoria* (Mohn et al., 2009), *A. sinensis* (Jin et al., 2012; Ma et al., 2015), *O. diffusa* (Chen et al., 2016)), triterpenoids (*G. uralensis* (Wang et al., 2013), *P. vulgaris* (Bai et al., 2016), *S. flavescens* (He et al., 2015)), quinones (*P. cuspidatum* (Peng et al., 2013)), iridoids (*O. diffusa* (Chen et al., 2016)), *S. flavescens* (He et al., 2015), and phtalides (*A. sinensis* (Jin et al., 2012; Ma et al., 2015)), among others. Thus, this TCM formula is representative of typical TCM formulae and exhibits a very complex composition. In order to study the contribution of every herbal drug to this multi-herb formula and to rationally select markers representative of each of the 10 herbs, the formula and each individual herb were extracted by decoction. The 10 individual herbs and one formula are abbreviated below as 10H and 1F.

**FIGURE 1:**
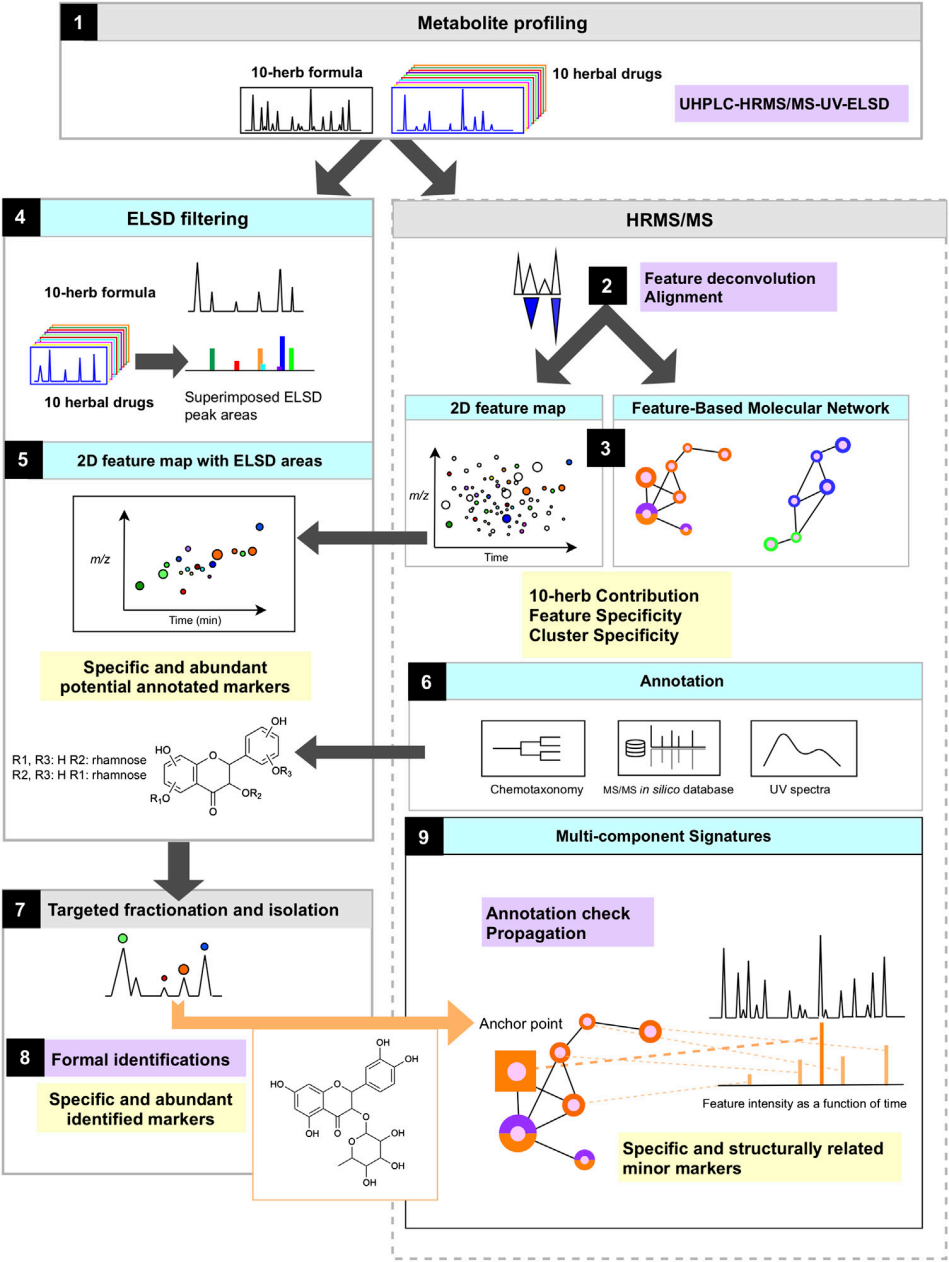
Scheme of the study workflow and analytical strategy to evaluate the metabolome contribution of each herb, identify specific markers of the formula and establish multi-component signature of each herb.

**FIGURE 2:**
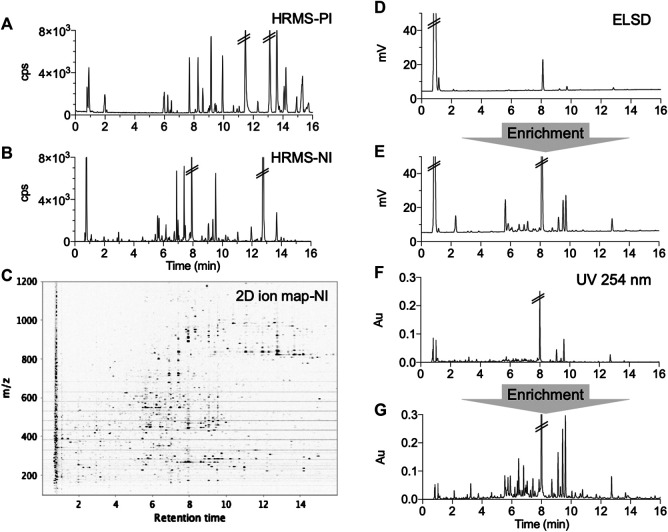
UHPLC metabolite profiling of the formula with different detections before and after enrichment: **(A,B)** complementary positive (PI) and negative (NI) ionization chromatograms, **(C)** 2D ion map presenting all ions detected in NI, **(D,E)** ELSD chromatograms before and after SPE enrichment showing the unretained polar metabolites (mainly saccharides), **(F,G)** UV chromatograms (254 nm) before and after enrichment.

For a comprehensive metabolome characterization, the strategy applied in this study (Figure 1) is based on the UHPLC-HRMS/MS metabolite profiling of the formula as well as of the 10 herbal drugs included in its composition (Figure 1.1). The 10H and 1F are analyzed by FBMN, which allows 1) to align all HRMS features extracted by peak-picking and represent them in the form of a colored ion map (Figure 1.3) and 2) to group all features according to structural similarity by molecular networking (Figure 1.3).

Since in such an untargeted LC-MS metabolite profiling, the MS response is not proportional to the amounts of components, all extracts are analyzed by ELSD in order to highlight the main components of each herb only within the formula (Figures 1.4, 1.5). Altogether, this permits to localize precisely abundant markers specific to each herb in the formula.

All main components are annotated based on their corresponding HRMS and HRMS/MS spectra. The annotation strategy combined FBMN for grouping structurally related features, spectral comparisons against a comprehensive *in silico* MS/MS spectral database of secondary metabolites and taxonomic filtering of the results (Figure 1.6). Their isolation is performed by a direct fractionation of the multi-herb formula at the gram scale (Figure 1.7). This process ideally both provides specific standards markers for all herbs in one step and validates the identification of annotated components (Figure 1.8). The isolated components are then used as anchor points in the FBMN to propagate structural information to the clustered features, which allows retrieving groups of herbal-specific components, which we refer to as specific multi-component signatures (Figure 1.9).

### Metabolite Profiling of the Formula and Each Individual Herb

Decoctions of the 10H and 1F were prepared according to the traditional recipe and were then freeze-dried (Section *Preparation of Extracts, Formula and Enriched Formula*). The formula was first analyzed by reverse phase (RP) UHPLC-PDA-ELSD and UHPLC-HRMS using a broad linear gradient (Figures 2A–F). The ELSD trace revealed a high quantity of polar constituents (mainly sugars) (Figure 2D), while the HRMS metabolite profiling highlighted secondary metabolites (Figures 2A–C). An SPE enrichment procedure was implemented to improve the detection of secondary components by ELSD and PDA (Figures 2E,G).

**FIGURE 3:**
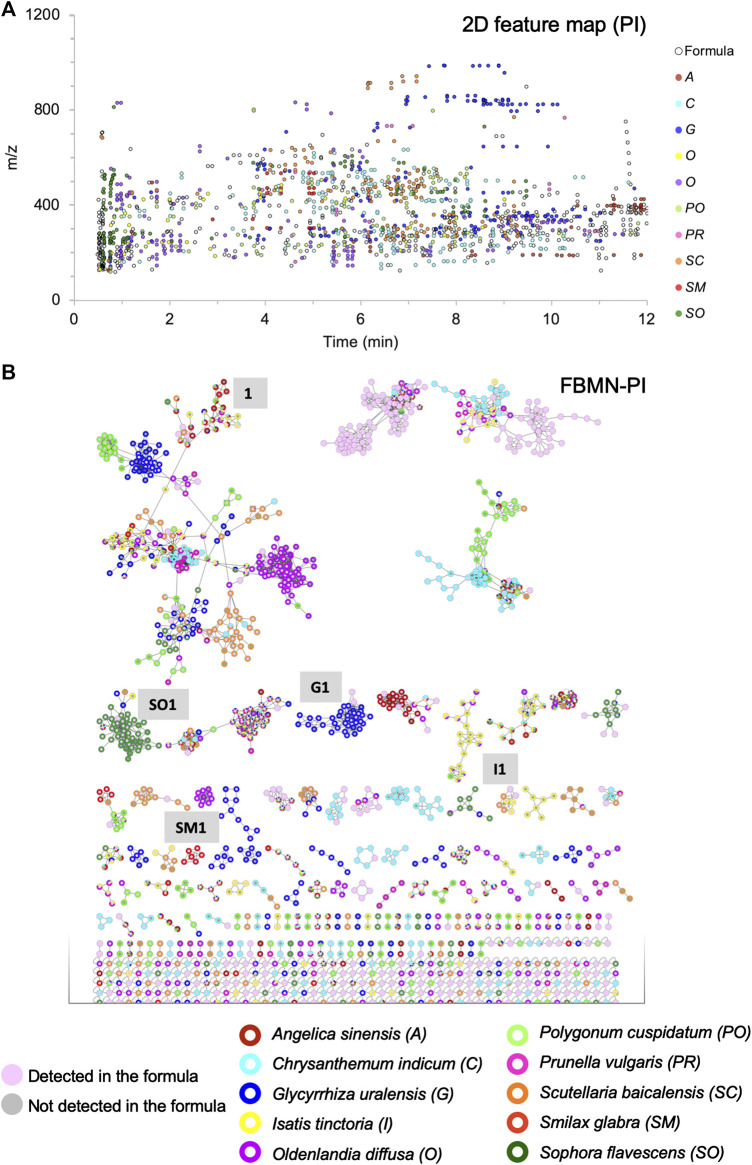
Visualization of the contribution of the 10 herbs to the formula, in which each herb is represented by a different color: **(A)** 2D feature map in PI: each black circle represents a feature detected in the formula, the size of the circles is fixed and equal for all features. The inner color of the circle indicates that the feature is specifically detected in one of the 10 herbs (90% specificity threshold). **(B)** FBMN in PI for the organization of the MS/MS spectra of all features presented in **(A)**, with the same color coding and fixed node size.

To study the contribution of each herb in the formula, the 10H and 1F extracts were then profiled by UHPLC-HRMS/MS in both positive ionization (PI) and negative ionization (NI) modes using an optimized gradient in RP conditions. All analyses were performed in the data dependent mode to generate both HRMS spectra for molecular formula assignments and corresponding HRMS/MS fragmentation spectra on most detected HRMS features. Data were processed with MZmine software (Pluskal et al., 2010), to generate an aligned peak list of all features (Figure 1.2). This list of aligned features has been employed in two ways. On the one hand, it has been presented in the form of a 2D feature map, in which the features detected in the formula have been colored according to their occurrence in the individual herbs (Figures 3A, 5A and Supplementary Figures S1A, S2A). This display provided a first view of a qualitative contribution of each herb to the formula. On the other hand, the list of aligned features was submitted to the Global Natural Products Social Molecular Networking (GNPS) platform to organize the HRMS/MS fragmentation spectra in clusters of features sharing similar fragmentation and thus potentially grouping structural analogues (Wang et al., 2016a; Nothias et al., 2020). In the resulting FBMN, the same color code as in the 2D feature map was applied to represent each herb (Figure 3B and Supplementary Figure S1B). Finally, peaks were annotated based on HRMS/MS spectra matches against an *in silico* database (ISDB-DNP) (Allard et al., 2016).

**FIGURE 4:**
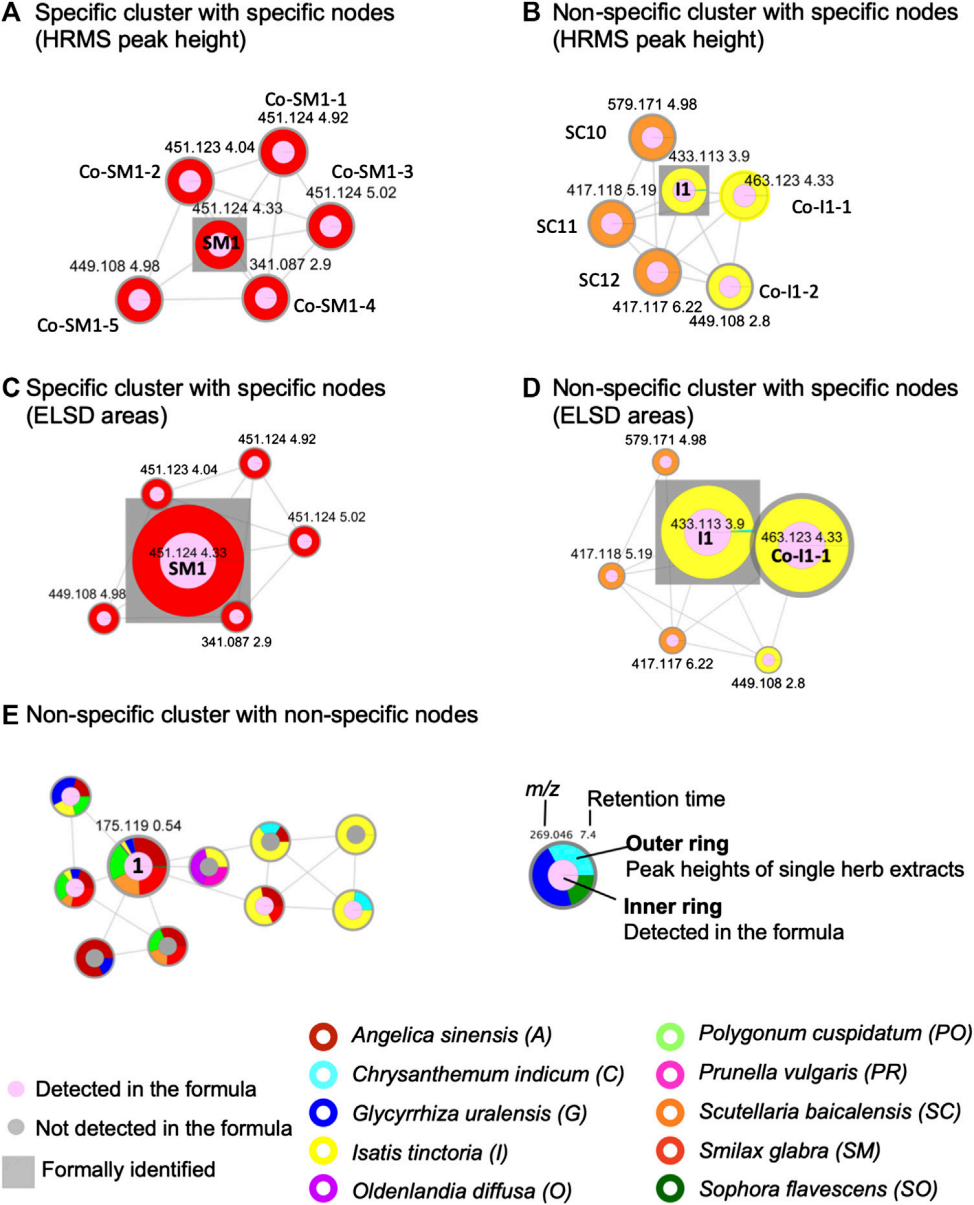
Selected examples of cluster and node specificity in the FBMN-PI: **(A)** specific cluster for *S. glabra* (100%) exhibiting only specific nodes (the size node is proportional to the HRMS height of the peak), **(B)** non-specific cluster (50% *S. baicalensis* and 50% *I. tinctoria*), with nodes each specific to one single herb (specific components of each herb sharing common structural type for both herbs) (the size node is proportional to the HRMS height of the peak), **(C)** the same cluster as in **(A)** with the node sizes proportional to the ELSD areas, **(D)** the same cluster as in **(B)** with the node sizes proportional to the ELSD areas, **(E)** non-specific cluster common to several herbs with a node annotated as an ubiquitous component, arginine (1) (the node size is proportional to the HRMS height of the peak). For arginine (1), the colors shows the following specificity percentage: *A. sinensis* 28%, *G. uralensis* 5%, *I. tinctoria* 3%, *O. diffusa* 1%, *P. cuspidatum* 21%, *S. baicalensis* 18%, *S. glabra* 24%, *S. flavescens* 1%) (the size node is proportional to the HRMS height of the peak). Each herb is represented by a different color on the external ring. On the nodes, the numbers indicate feature *m*/*z* and retention time. The square is used to label formally identified components. Codes such as **Co-Sm1-2** or **SC10** referred to annotations, see Supplementary Tables S5, S9, S10.

The FBMNs resulting from this processing allowed the organization of the metabolite profiling in both ionization modes (Table 3). The FBMN allowed assigning a given node to each feature for which a fragmentation spectrum was recorded. Any feature was detected in the formula and in none, one or several herbs, or in some herbs and not always in the formula. At the node level, in order to keep this information, all features were displayed by nodes having an inner circle and an external ring (legend Figure 4). The inner circle was of pink color if detected in the formula and of gray color if not detected in the formula but only in the individual herbs. The size of the nodes was proportional to the intensity of the peak in the formula. On each node, the external ring indicated in which individual herb(s) the feature was detected according to the corresponding herb color code. A ring with a single color was characteristic of a feature specific to a single herb (Figures 4A,B), while multiple colors in the ring indicated the presence of this feature in all corresponding herbs (Figure 4C). The colored ring area was proportional to the feature intensities in each herb.

**TABLE 3:** Description of FBMNs in both ionization modes.

	Positive ionization	Negative ionization
Number of nodes	2,713	2,058
Number of clusters	148	80
Number of clustered nodes	1,258	573
Number of ISDB-DNP annotated nodes	1,698 (63%)	1,126 (55%)

Molecular networking allows nodes to be grouped according to the similarity of their associated HRMS/MS spectra, and thus similar structures were grouped in clusters. At the cluster level, node mapping highlighted three main types of clusters: 1) monochrome clusters with a single color on the node external ring, i.e., specific analogues series for a given herb (Figures 3B, 4A), 2) multi-color clusters with a single color on the node external ring, i.e., specific and structurally related components from different herbs (Figures 3B, 4B) and 3) multi-color clusters with multiple colors of the node external rings, i.e., ubiquitous components present in different herbs (Figures 3B, 4E).

The FBMN provided a very detailed view of the specificity/ubiquity of each feature and cluster across all 10H and 1F extracts. This also allowed a global qualitative vision of the contributions of each herb to the formula and the relationship between all features. In order to precisely assign the features present in the formula that were herb-specific and to identify clusters of features that showed series of analogues that were also very specific, a script was written as an alternative to the visualization classically used in FBMN. This script permitted to express the specificity numerically as a percentage of relative intensity at the node and cluster levels (Section *Node and Cluster Specificity* and Supplementary Figure S4)

The script considered the feature heights after the alignment of the 10 herbs of the formula and the repartition of the features in clusters. At the node level, similarly to the ring visualization, the script enabled to express as a percentage the relative height intensity of one feature in the 10 herbs (Supplementary Figure S4). This relative height intensity was named the *specificity percentage* of a feature (Table 1). Fully specific features, presenting a 100% specificity percentage, were highlighted. Due to the sensitivity of the HRMS detection and the ubiquity of natural components, in practice, the specificity percentage threshold was set at 90% for a given herb. The number of specific peaks (90% threshold) in the formula was 1,030 in PI (65%) and 734 in NI (72%) (the numbers of specific features for each herb are presented in Supplementary Figures S5–S7).

**FIGURE 5:**
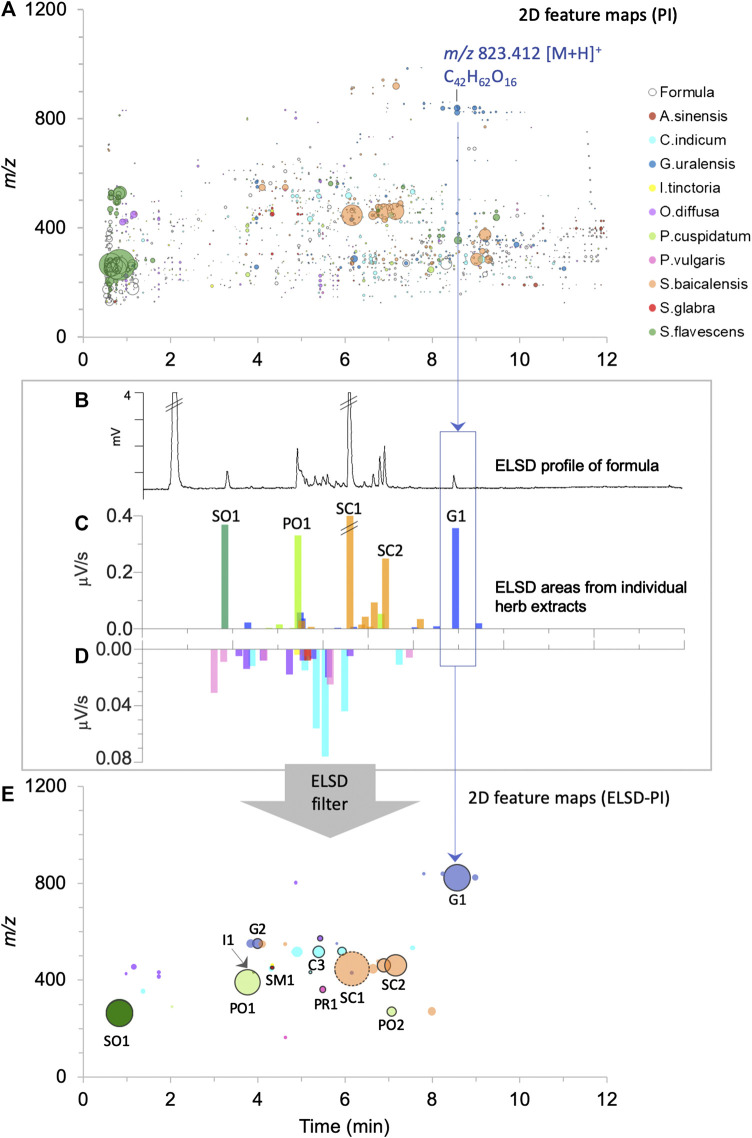
Visualization of the contribution of the 10 herbs to the formula normalized by ELSD filtering to consider semi-quantitative relationship between components. Each herb is represented by a different color as in Figures 3, 4: **(A)** 2D feature map in PI: the size of the circles is proportional to the feature height intensity in the formula, only features detected in the formula are represented. The inner coloration of the circles indicates that the feature is specifically detected in one of the 10 herbs (90% specific threshold), **(B)** ELSD profile of the formula, **(C,D)** bar plots proportional to the chromatogram retention time dimension with superimposed ELSD areas of the individual herb extracts, presented in two scales, from 0 to 0.4 μV/s in **(C)** and from 0 to 0.08 μV/s in **(D)**. **(E)** 2D feature map (PI) presenting the features to which ELSD peaks were assigned. The size of the dots is proportional to ELSD areas, with the exception of the dot with a dashed circle (SC1), where half of the area value is shown to improve visualization (very major component). The dots with a black outer circle represented components that have been formally identified.

To visualize these specific features, all features detected in the formula were represented in the form of a 2D feature map comparable to the raw LC-HRMS ion map (Figures 1.3, 2C, 3A, 5A for PI; Supplementary Figures S1A, S2A (NI)). Features detected in the formula and found in a single herb were tagged with the herb color code. Those that were not specific were represented by a white dot with a black circle. The 2D feature map also enabled to highlight chromatographic information, which are not easily visible in the FBMN.

The specific contributions varied from one herb to another: *Glycyrrhiza glabra* provided a high number of specific features (189 (18.3% of all specific features) and 168 (22.9%), respectively, in PI and NI), while the amount of dried herb represented only 3.4% of the crude herb mixture before extraction. Some of these specific features (blue color dots in Figures 3A, 5A; Supplementary Figures S1A, S2A) were observed around 8 min with *m*/*z* > 800, and annotated as oleanane triterpenoids. In the FBMN, these features were organized into a specific cluster, which is highlighted by the blue color code blue (Figure 3B, code G1; Supplementary Figure S1B). In contrast, *Smilax glabra* which was one of the main dried herbs in formula (20.7%) had only 20 specific features (1.9% of all specific features) and 40 (5.4%) in PI and NI modes, respectively (red color dots mainly around 4–5 min in Figures 3A, 5A and Supplementary Figure S1A). Some of the features from *Smilax glabra* were also organized in a specific cluster (Figure 3B, code SM1; Supplementary Figure S1B). In PI, several features of *Sophora flavescens* were highlighted before 2 min (Figures 3A, 5A, dark green dots), annotated as quinolizidine alkaloids and organized in a specific cluster in the FBMN-PI (Figure 3B, code SO1). Overall, the feature map representation also highlighted a good spreading of all specific features across *m*/*z* and retention time dimensions.

Similarly to the treatment at the node level, the script enabled to describe with a percentage the specificity of each cluster to a given herb (*cluster specificity percentage* (Table 1)). For each cluster, the averages of the specificity percentages for each herb were calculated. The script also allowed to sort the clusters according to their cluster specificity percentages for each herb (Supplementary Figure S4). A threshold of 75% was arbitrarily chosen to identify clusters mainly specific to one of the 10 herbs. The cluster specificity percentage was also used to describe clusters whose contributions came from several herbs. Among all PI clusters, 36 clusters (24% of all clusters) were found to be specific to one of the 10 herbs. These specific clusters contained 346 nodes (13% of all nodes). In NI, 23 clusters (29% of all clusters) were found to be specific to one of the 10 herbs, which concerned 207 nodes (10% of all nodes) (Supplementary Figure S8).

### Selection of the Most Abundant Components of Each Herb by Evaporative Light Scattering Detection Filtering

The 2D feature map and the FBMN provided a detailed visualization of the metabolome of the 10 herbs formula in terms of herb specificity and contribution which were also estimated by calculating specificity percentages. However, this analysis did not provide rational information in terms of component abundance, since untargeted HRMS/MS detection is not quantitative. To further select abundant components of interest, ELSD was used as a complement for semi-quantitative and quasi-universal detection to filter the features related to the most abundant components (Figure 1.4).

UHPLC-ELSD peak areas of the 10 herbs plotted in the form of bar plots (Figures 5C,D) highlighted 47 peaks representing the main components (Supplementary Figures S9, S10 present the original chromatograms). Four herbs (*Scutellaria baicalensis*, *Polygonum cuspidatum*, *Glycyrrhiza uralensis*, and *Sophora flavescens*) presented rich ELSD traces with each several main components (Figure 5C). On the contrary, other herbs contributed only to few and low intensity UHPLC-ELSD signals (Figure 5D): *Isatis tinctoria* yielded two weak signals and *Smilax glabra* only one, while no signal was recorded for *Angelica sinensis* at the exception of metabolites with no retention in RP conditions (Supplementary Figures S9, S10).

The features of the 47 main components were localized thanks to the 2D feature maps (see Section *UHPLC-UV-PDA-HRMS/MS System* for a detailed description), and their specificity was verified as described previously. An ELSD peak was considered as specific if the corresponding feature has a specificity percentage above 90% in both ionization modes. Five main components were considered as non-specific under this criterion. In addition, five ELSD peaks were not detected in the formula decoction.

**FIGURE 6:**
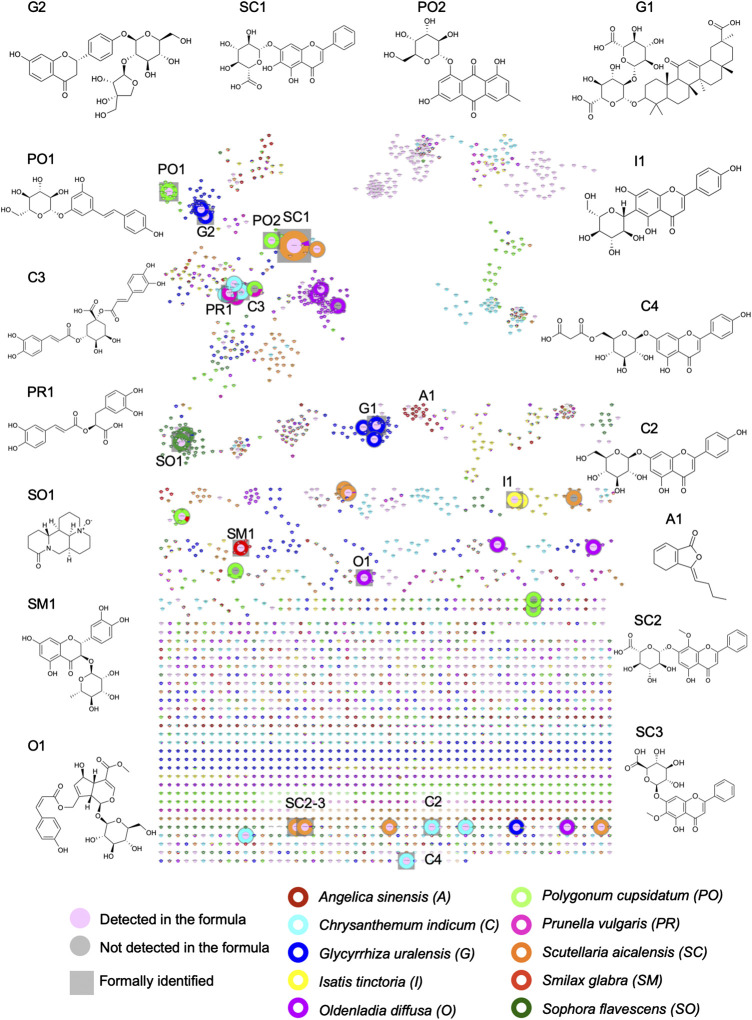
Visualization of the ELSD detected peaks in the FBMN-PI and structures of all formally identified components. In this representation of the FBMN, the size of the nodes is proportional to ELSD areas. See Table 4 for the name of the components.

**FIGURE 7:**
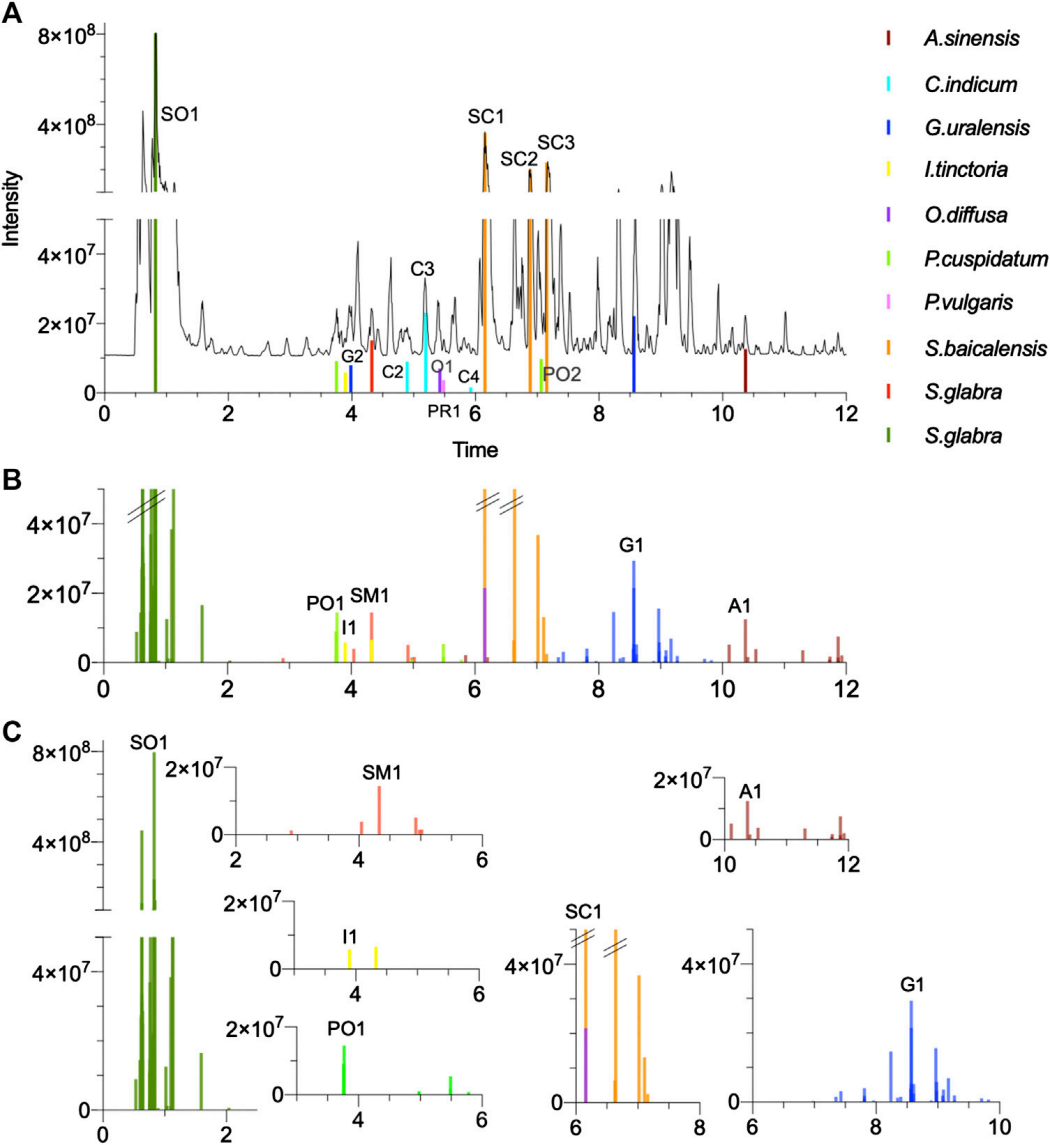
Multi-component signatures extracted from the FBMN (Figures 3B, 6) and represented in the form of bar chromatograms presenting the feature height intensity as a function of retention time in PI: **(A)** metabolite profile of the formula showing all formally identified components, **(B)** bar chromatograms showing seven multi-component signatures. **(C)** multicomponent signatures for *S. glabra* and its marker astilbin (SM1), *I. tinctoria* and isovitexin (I1), *P. cuspidatum* and *E*-piceid (PO1), *A. sinensis* and ligustilide (A1), *G. uralensis* and uralsaponin A (G1), *S. flavescens* and oxymatrine (SO1) and *S. baicalensis* and baicalin (SC1). All *X* axes represent time dimension, all *Y* axes feature height intensity. See Supplementary Tables S2–S11 for annotations.

The 42 remaining abundant herb components (40 and 34 peaks, respectively, in PI and NI) were all found to be specific (90% threshold) in the formula and were highlighted in the 2D feature map and in the FBMN by mapping their ELSD intensities instead of HRMS intensities (Figures 1.5, 4C,D, 5E, 6, 7A and Supplementary Figures S6E, S7). Among them, 26 and 24 peaks had a specificity percentage of 100%, respectively, in PI and NI. Twelve and eight peaks had specificity percentages between 95 and 99%, respectively, in PI and NI and two peaks between 90 and 94% in both modes (Supplementary Figure S3). As shown here, the threshold of specificity percentage had to be set at 90% to consider several abundant peaks in the formula. The lack of specificity was not linked to potential carryovers effects by carefully considering the order of analysis. The low abundant detected features were thus due to minor contributions from other herbs of the TCM. However, depending on the formula composition, this threshold can be adapted.

Among the five main components that were not specific (below 90% threshold) are two main peaks of *Scutellaria baicalensis* (**SC1** and **SC2**). The component **SC1** was shared with *Oldenlandia diffusa* (94–6% in PI and 83–17% in NI) (Chen et al., 2016), while **SC2** was additionally detected in *Chrysanthemum indicum* (Figure 7B). Although not perfectly specific to *Scutellaria baicalensis*, these peaks were considered for further identification, being the most abundant components of the formula. The other non-specific features concerned an ELSD peak of *Chrysanthemum indicum* (**C6**), annotated as chlorogenic acid, which was shared in small amounts with three other herbs, as well as one peak from *Polygonum cuspidatum* (**PO4-F**), shared with *Smilax glabra*. Finally, one peak from *Prunella vulgaris* (**PR3**) was shared with three other herbs in small amounts. It has to be noted that these three peaks (**C6**, **PO4-F**, **PR3**) were not the main ELSD peaks for the corresponding herbs.

The features selected by ELSD filtering were all annotated in detail. The automated annotation strategy of all features combined molecular formula assignment and HRMS/MS comparison with the *in silico* spectral database. Since the filtered features (40 in PI and 34 in NI) were much less than the total number of annotated features (1,698 in PI and 1,126 in NI), their annotations were verified and completed in detail (Supplementary Section S5 for a detailed description of the annotation workflow). For all annotations obtained, the top six structure candidates in both modes were checked for consistency in previously reported components for each herb (taxonomic consistency). When available, UV spectra were used to confirm the annotations or to discriminate them. The strategy enabled the partial annotation of all main components, however, in some instances, exact isomer differentiation was not possible (Supplementary Section S5 and Supplementary Tables S2–S11).

At this stage of the workflow, potential markers that were both specific and ELSD detected were annotated by combining several levels of spectral and taxonomic information. This allowed the annotation of one or more potential markers for each herb, that are specific in the formula with proposals of structures that are taxonomically coherent.

### Targeted Isolation of Potential Markers From the Formula

To obtain these markers rapidly in view of the standardization of the formula, the information collected was used to target their isolation from a large quantity of the formula itself rather than initiating multiple isolations on each individual herb. This step was also used to confirm the annotations made. For this, a large-scale fractionation on MPLC was attempted directly on the enriched formula extract (Figure 1.7). The chromatographic conditions were first optimized using an HPLC analytical column packed with the same stationary phase than the MPLC column. The optimized HPLC conditions were then geometrically transferred to the preparative scale (Challal et al., 2015) (Section *Isolation of the Markers*). The fifty MPLCs fractions were profiled by UHPLC-HRMS and ELSD in the same chromatographic conditions. Peak detection in the whole dataset with MZmine permitted the localization of the features of interest in related fractions. The ELSD traces of the selected fractions were used to assess purity. Six markers from five herbs were directly obtained from the MPLC fractions. The other markers were found in five additional fractions which were further submitted to optimized semi-preparative isolation. Altogether, the process yielded thirteen markers specific to seven herbs of the formula. These isolated components were subsequently analyzed by 1D and 2D-NMR for unambiguous identification (Supplementary Section S7 for precise descriptions). This provided a definitive discrimination in cases where isomers could not be univocally annotated.

Based on this strategy, 13 specific standards were obtained for seven herbs of the formula. It was however not possible to obtain standards for three herbs (*Angelica sinensis*, *Prunella vulgaris* and *Isatis tinctoria*). For these herbs, the annotated features were ligustilide (**A1**) for *Angelica sinensis*, rosmarinic acid (**PR1**) and salviaflaside (**PR2**) for *Prunella vulgaris* and isovitexin (**I1**) and isoscoparin (**I2**) for *Isatis tinctoria* ( Supplementary Tables S2, S5, S8). This difficulty was not surprising, because the ELSD profiling revealed that these components were present in low quantities. Based on the annotations, in these cases, three standards were purchased and confirmed the annotations of **A1**, **PR1** and **I1** after comparison of their retention time and HRMS/MS spectra. All formally identified markers are summarized in Table 4 (see their structures in Figure 6 and their localization in the metabolite profiling in Figure 7A and Supplementary Figure S15 (NI)). Finally, *Scutellaria baicalensis* and *Chrysanthemum indicum* were represented by three markers, *Glycyrrhiza glabra* and *Polygonum cuspidatum* by two markers, whereas *Angelica sinensis*, *Isatis tinctoria*, *Oldenlandia diffusa*, *Prunella vulgaris* and *Sophora flavescens* were represented by one marker.

**TABLE 4:** Formally identified markers.

Herb	Codes	Formally identified markers	Molecular formula	Class of compounds
*A. sinensis*	A1	Ligustilide	C_12_H_14_O_2_	Isobenzofuran
*C. indicum*	C1	1,3-Dicaffeoyl-epi-quinic acid	C_25_H_24_O_12_	Caffeoylquinic acid
	C2	Cosmosiin	C_21_H_20_O_10_	Trihydroxyflavone *O*-glucosides
	C4	6″-Malonylcosmossiin	C_24_H_22_O_13_	
*G. uralensis*	G1	Uralsaponin A	C_42_H_62_O_16_	Oleanane triterpenoid
	G2	Liquiritin apioside	C_26_H_30_O_13_	Flavanone
*I. tinctoria*	I1	Isovitexin	C_21_H_20_O_10_	Flavone *C*-glucoside
*O. diffusa*	O1	10-*O*-*p*-*cis*-Coumaroyl scandoside methyl ester	C_26_H_30_O_13_	Scandoside
*P. cuspidatum*	PO1	*E*-piceid	C_20_H_22_O_8_	Stilbene
	PO2	Emodin-8-*O*-glucoside	C_21_H_20_O_10_	Anthraquinone
*P. vulgaris*	PR1	Rosmarinic acid	C_18_H_16_O_8_	Rosmarinic lignan
*S. baicalensis*	SC1	Baicalin	C_21_H_18_O_11_	Flavone *O*-glucuronides
	SC2	Wogonoside	C_22_H_20_O_11_	
	SC3	Oroxyloside	C_22_H_20_O_11_	
*S. glabra*	SM1	Astilbin	C_21_H_22_O_11_	Flavanone
*S. flavescens*	SO1	Oxymatrine	C_15_H_24_N_2_O_2_	Quinolizidine alkaloid

### Metabolome Analyses for Selecting Multi-Component Signatures

**FIGURE 8:**
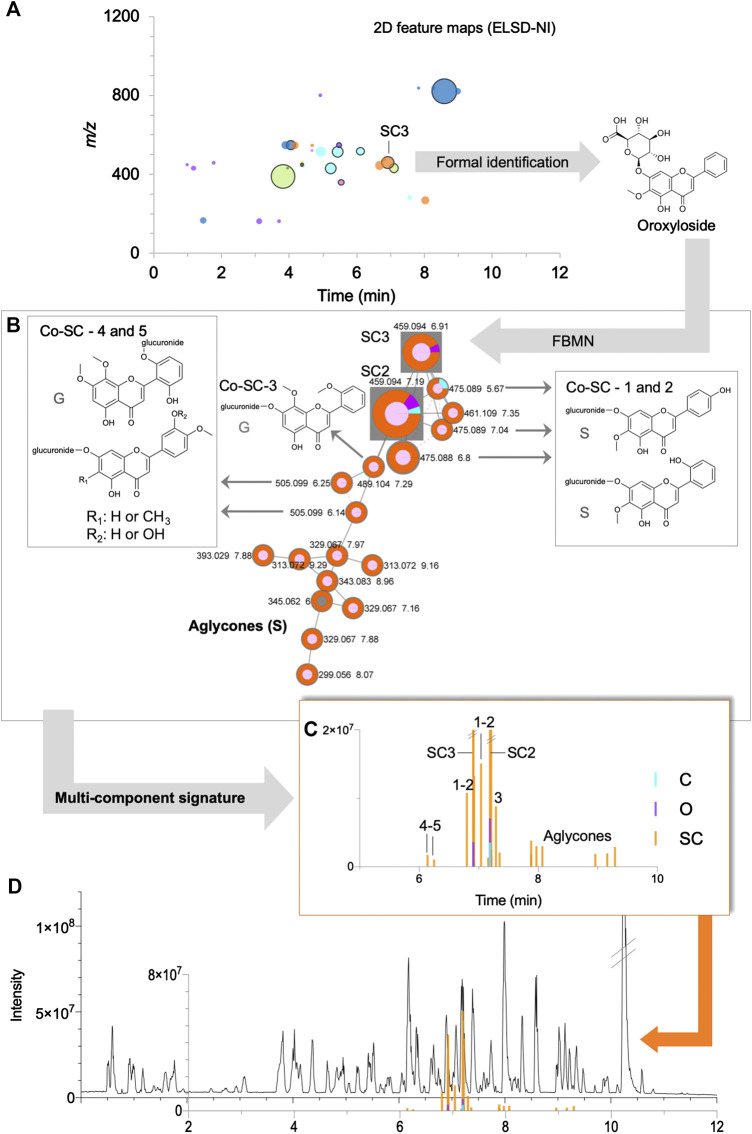
Example of the detailed mining of a multi-component signature for *Scutellaria baicalensis* in the formula: **(A)** 2D feature map (NI) filtered by ELSD (see Figure 5E), **(B)** specific cluster in FBMN-NI containing the identified markers **SC2** and **SC3** and their related annotated nodes, some with structures described for the species (S) or genus (G), **(C)** bar chromatograms showing the corresponding multi-component signature with their chromatographic data, **(D)** UHPLC-HRMS/MS metabolite profile of the formula in NI in regards of the multi-component signature. Codes such as **Co-SC-2** referred to annotations, see Supplementary Table S9.

All formally identified markers were used as anchor points in the FBMN to correct ambiguous annotation, and to highlight minor components that could serve as potential multi-component signatures. An *anchor point* is defined as a node whose formal identification is based on NMR after targeted isolation or by comparison of two independent and orthogonal data with a pure standard (i.e., retention time and *m*/*z*) with a pure standard (level 1 identification according to the Metabolomic Standard Initiative (MSI) (Sumner et al., 2007) (Table 1). In a FBMN, these anchor points increase the annotation confidence for all related nodes in a given cluster. The analysis of the clusters containing such anchor points revealed some series of analogues structurally similar to the identified components and whose specificity could be rapidly evaluated. These analogues were annotated by manually propagating the information provided by the anchor points and were labeled by keeping reference to the identified marker (**Co-SC1-1**, with **Co** for co-marker) (Supplementary Tables S2–S11). This manual propagation consisted first in sorting the *in silico* annotation, by keeping the structures of the same chemical class as the anchor point as the most relevant. In cases of multiple relevant annotations, the sorting was done according to the chemotaxonomic relevance. Such series of clustered annotated analogues were defined as *multi-component signatures* specific to each herb marker in the formula. Together, the set of the 10 herbs multi-component signatures provide a kind of chemical passport specific to the formula. In order to visualize these multi-component signatures as a subset on the main chromatogram, the peak data from the selected clusters were exported and represented as *bar chromatograms* (Table 1), which presented the intensity of HRMS peaks as a function of time (Figures 7B,C, 8C,D and Supplementary Figures S15–S19).

Several categories of multi-component signatures were distinguished: 1) herb-specific clusters containing a formally identified marker (Figure 8 and Supplementary Figures S16, S17, S19D), 2) non-specific clusters containing a formally identified marker (Supplementary Figures S17, S19B) and 3) specific clusters with no formally identified marker and taxonomically relevant annotations (Supplementary Figure S18).

#### Category 1: Herb-Specific Clusters Containing a Formally Identified Marker

This ideal category highlighted identified markers and their structurally related analogues that are herb-specific in the formula. This was the case for seven clusters in both ionization modes with identified markers representing seven herbs (**A1** and **SO1** in PI, **SC2**, **SC3**, **C3** and **I1** in NI, **GI** and **SM1** in PI/NI) (Supplementary Tables S2–S5, S9–11). The analogues detected were often isomers of known markers, as shown for *Angelica sinensis* (**A1**, **Co-A1-1** and **2**), *Chrysanthemum indicum* (**C3**, **Co-C3-1** to **5**) *Glycyrrhiza uralensis* (**G1**, **Co-G1-1** to **3**) and *Smilax glabra* (**SM1**, Co-SM1-1 to 3) (Supplementary Tables S2–S4, S10). Concerning *Sophora flavescens*, the cluster consisted of quinolizidine alkaloids analogues of oxymatrine (**SO1**) that were not isomeric structures (**Co-SO1-1** to **6**, Supplementary Table S11). In the case of *Scutellaria baicalensis*, three markers were identified (**SC1**, **SC2** and **SC3**) but none were 100% specific. In order to find 100% specific makers, the nodes associated either with **SC1** or the ones associated to the cluster with **SC2** and **SC3** were inspected in detail (Supplementary Table S9). This revealed that the cluster with **SC2** and **SC3** had structurally related nodes that were 100% specific to the herb (Figures 8B,C). These nodes were annotated as analogues with additional hydroxyl or/and methyl groups. Similarly, methylated flavone aglycones were present in the same cluster.

#### Category 2: Non-Specific Clusters Containing a Formally Identified Marker

This type of clusters contained nodes specific to different herbs which were structurally related. This was the case for *Polygonum cuspidatum* and *Prunella vulgaris*.

For *Polygonum cuspidatum*, its marker (*E*-piceid (**PO1**), a resveratrol derivative, was detected in a large cluster shared between all herbs of the formula. Within this large cluster, several analogues of **PO1** were specific to this herb and organized in a sub cluster (**Co-PO1-1** to **4**, Supplementary Table S7 and Supplementary Figure S17)). Their presence in this cluster showed that these features shared structural (at least spectral) similarities with features from other herbs.

Another mixed cluster in NI had a cluster specificity percentage of 68.9% for *Prunella vulgaris*, including **PR1**, surrounded by nodes coming from *Scutellaria baicalensis* and *Sophora flavescens* (Supplementary Figure S19B). The specific nodes attributed to *Prunella vulgaris* which were annotated as salviaflaside (**Co-PR1-1**) and danshensuan C (**Co-PR1-2**) were considered as co-markers of the formally identified rosmarinic acid (**PR1**) (Supplementary Table S8).

#### Category 3: Specific Clusters With No Formally Identified Marker

In some cases, a marker may be in a cluster that cannot be used for a multi-component signature. The marker of *Oldenlandia diffusa* (**O1**) represented such a case. It was linked only with features with the same retention time (in-source adduct or fragment). In such a case, clusters containing only specific nodes can easily be evidenced by the proposed workflow to interpret the specificity and can, in addition to the marker, contribute to the traceability of this herb in the formula. For this herb, specific clusters contained features annotated as flavonols di-glycosides (**Co-O-1** to **3** in NI and PI), which were previously described for this species and could serve as multi-component signature (Supplementary Table S6 and Supplementary Figure S17).

## Discussion

This exploratory study enabled a comprehensive overview of the chemical composition of a 10 herb TCM formula and highlighted the contribution of each herb at the metabolite level. The proposed analytical workflow was oriented to generate useful information for the establishment of quality control (QC) methods that consider the specificity of markers, their abundance, and the presence of multi-component signatures that could serve as co-markers. The strategy combined untargeted metabolite profiling with ELSD detection, which permitted, on the one hand, identifying at least one main marker per herb and assigned its specificity in the formula. On the other hand, it permitted the extraction of specific multi-component signatures from all the information gathered in the FBMN.

### Feature-Based Molecular Network for Multi-Herb Formulae Composition Assessment

To our knowledge, this study is the first to explore the potentialities of FBMN for documenting the detailed composition of a TCM multi-herb formula. Molecular networking was previously successfully used to annotate components of interest in single TCM herb extracts (Pan et al., 2019; Wang et al., 2019; Wang et al., 2020b).

Metabolite profiling processing through the FBMN workflow directly supported the visual inspection of the node and cluster specificities. However, this information-rich dataset is difficult to interpret as such. To summarize the specificity information, a script was developed to calculate the specificity of the features and clusters according to the 10 herbs and to display this information in the chromatographic dimensions (Figures 3A, 7 and Supplementary Figure S4). This generic data processing enabled all data from single herbs to be linked to the formula, provided that a careful alignment of all features was performed. This fully automated task during MZmine 2 processing was key for providing an interpretation of the contribution and specificity of each herb to the formula. Thanks to the data processing, the contribution of each herb to the formula was visualized in the form of the 2D feature map (Figures 3A, 5A), which provided a qualitative and comprehensive view.

The data generated by this untargeted exploratory metabolite profiling study were used to investigate the contribution of each herb to a given formula to assist the development of improved QC methods. The same TCM metabolome dataset could also have been processed to describe the different chemical classes contained in the formula and possibly annotate as many features as possible. The proposed workflow to capture the specificity of each node and cluster could also be employed, for example, to focus on the ubiquitous features or the features present only in the formula.

### Evaporative Light Scattering Detection Filtering for the Selection of Abundant Components

From the QC perspective, the semi-quantitative aspects between the different components are important (Yang et al., 2017). In this work, we decided to integrate the ELSD data into the dataset obtained from the metabolite profiling. The ELSD detection allowed us to filter the large dataset and to focus on less biased data in semi-quantitative terms. As shown in Figures 4A–D, 5, 6, complementary and simplified visualizations could be generated, which present the semi-quantitative information in addition to the contribution and specificity of each feature.

The combination of ELSD data with specific features has permitted locating potential markers precisely in the metabolite profile. This can support the targeted purifications of markers. These data can also be compared with data from the literature, and, for annotated markers, a choice among those that are commercially available is also possible to obtain specific standards and confirm the annotation process. In this study, a direct purification from the formula was attempted to identify most of the markers of interest and to obtain sufficient quantities of pure markers to develop a QC method. While this was successful for the most abundant components, the less abundant markers were obtained in small quantities or not pure enough for developing a QC method. Isolation from each individual herb would, therefore, be required for the less abundant selected markers.

### Marker Selection

This workflow allowed the selections of at least one identified marker for each herb in the formula, ensuring a degree of specificity which is unique to this formula. This innovative approach is unbiased, as it is directly related to data observation. *A priori*, it can be applied to other formulae and could be generic, but further studies are needed to demonstrate it. The markers revealed by this approach are compared below with the ones proposed by the European (PhEur) and Chinese (ChP) Pharmacopoeias to evaluate their relevance. Among them, four markers were identical to the markers proposed by the Pharmacopoeias (**A1**, **G1**, **SC1**, **SM1** (Tables 2, 4)) for *Angelica sinensis, Glycyrrhiza uralensis, Scutellaria baicalensis* and *Smilax glabra*. Three markers were closely related (oxymatrine (**SO1**) instead of matrine for *Sophora flavescens*, emodin-8-glucoside (**PO2**) instead of emodin for *Polygonum cuspidatum,* and 1,3-dicaffeoyl-epi-quinic acid (**C3**) instead of chlorogenic acid for *Chrysanthemum indicum*. For these three cases, the pharmacopoeial markers were detected but were less abundant based on ELSD detection or not specific. Additionally, in line with recently developed *single standard to determine multi-components methods* (SSDMC) for the QC of single TCM herb (Gao et al., 2009), our workflow highlighted wogonoside (**SC2**) as a second marker for *Scutellaria baicalensis* (Wang et al., 2017), and *E*-piceid (**PO1**) for *Polygonum cuspidatum* (Yang et al., 2015).

For the three other herbs of the formula, the pharmacopoeial markers were either not detected or/and not sufficiently specific. For *Oldenlandia diffusa* and *Prunella vulgaris*, the ChP suggested triterpene aglycones, oleanolic acid and ursolic acid, respectively. Such lipophilic compounds are difficult to extract by decoction and were not detected by RP UHPLC-HRMS/MS. Our workflow formally identified a specific scandoside derivative for *Oldenlandia diffusa* (10-*O*-*p*-*cis*-coumaroyl scandoside methyl ester (**O1**)), previously described for the genus (Otsuka et al., 1991), and whose analogues were reported for this species (Chen et al., 2016). For *Prunella vulgaris*, rosmarinic acid (**PR1**) was selected as a marker in this formula (Bai et al., 2016), since its specificity could be proved by our workflow, even if this component is reported to be occurring in many plants (Petersen and Simmonds, 2003). The analyses of the other components related to **PR1** showed the presence of specific analogues that support the herb traceability (Supplementary Figure S19 and Supplementary Table S8). For *Isatis tinctoria*, the ChP marker, indican, was not detected. Its PhEur marker, arginine, was present but our approach confirmed that such a primary metabolite was not specific and occurred in several herbs of the formula (Figure 4C, code 1). For this herb, three flavones-*C*-glycosides were found to be specific and one of them was formally identified as isovitexin (**I1**) (Supplementary Table S5) (Mohn et al., 2009).

### Multi-Component Signature for Improved Traceability

In addition to the single marker selection, the approach has permitted highlighting multi-component signatures for most herb markers in the formula, that were presented as bar chromatograms (Figure 7). For QC, the ability to demonstrate the presence of multi-component signatures (or at least a few components) is required to improve the traceability of each herb in a formula (Yang et al., 2017). Improved traceability is crucial to detect cases of adulterations that add a single marker to another herb or to a herb of poor quality (Czigle et al., 2018). If QC methods using LC-MS are not yet incorporated in the Pharmacopeias, new methods attempt to incorporate these advanced techniques. Recently, methods using LC-MS were proposed to check multiple components of a given herb used in different multi-herb formulae (Si et al., 2016), as well as multiple components representing three herbs of a given formula (Yao et al., 2016). In both cases, the selected markers were followed-up by Single Ion Monitoring (SIM). The workflow proposed by our study for unbiased and specific markers selection can thus be used prior to such targeted QC method. The data generated can be transferred to techniques adapted to routine QCs by simple MS detectors for both main markers and selected multi-component signature. For this, considering the complexity of the composition of a typical TCM, the quantitation of the specific markers could be performed by targeted MS detection, either by single ion monitoring (SIM) or multiple reaction monitoring (MRM). In addition, the targeted monitoring of the multi-component signatures can be made by selecting their SIM traces or more specifically by monitoring their MRM transition.

In addition, the metabolome of an herb can vary according to its cultivation and geographical origin (Yang et al., 2017). The choice of multi-component signatures selected in this study should be validated by comparing several batches of the same herb for example by metabolomics (Marti et al., 2014; Afzan et al., 2019; Sehlakgwe et al., 2020). As an example, for *Oldenlandia diffusa*, our workflow selected specific markers from two independent chemical classes previously described for this herb but not considered by the Pharmacopoeias. A large variation of the chemical composition of this herb was observed in different QC studies (Chen et al., 2016) and further investigation are required. Then, the reproducibility of our observations in several batches of the formula should be conducted.

### Improvement and Automation of Processing Tools

However, the proposed approach has its limitations. While FBMN approaches currently allow data integration in a fast and efficient way, automatic scripts to extract the relevant data, visualize them, and choose markers do not yet exist. In the context of this work, this data extraction was done manually and semi-automatically. Software developments should enable detailed reports to be generated from the raw data, in order to provide all the reference information needed to define specific markers adapted to each TCM formula.

Another limitation concerns the identification of markers. The annotation strategy used in this study combined HRMS/MS spectra comparison with *in silico* predicted fragmentation spectra, filtering with taxonomic data and manual evaluation of the clusters. The structural assignments of the main markers were validated by targeted isolation. The obtained matching indicated that the structure annotation of the minor associated components in the formula (at least at the precise class of compounds level) is trustworthy even if isomeric structure cannot fully be ascertained (Supplementary Tables S2–S11). For improving the annotation process, re-ranking the annotations considering chemotaxonomic data was recently proposed (Rutz et al., 2019), and open-access spectral databases that also incorporate chemotaxonomic data should be further developed. In this direction, the concept of Digital Reference Standard (DRS) was proposed in the TCMs area (Wang et al., 2016b) and the platform MASST was launched in GNPS to fulfill this goal (Wang et al., 2020a). Furthermore, the automatic propagation of annotations in clusters has been proposed and could complement the approach used in this study (da Silva Ricardo et al., 2018).

## Materials and Methods

### Plant Materials and Chemicals

All medicinal plants were obtained from Conba company (Zhejiang CONBA Pharmaceutical and Drug Research Development Corporation, Hangzhou 310052, Zhejiang, China) which purchased them to Jinhua City, China Pharmaceuticals Co., Ltd. The raw material quality was in accordance with the *Dictionary of Traditional Chinese Medicine* and the *Chinese Pharmacopoeia* according to the laws of the Chinese government. Plant names were verified with www.theplantlist.org on April 27, 2019 and are summarized in Table 2, which presents the codes used in this study. For the plant *Reynoutria japonica* Houtt., the synonym *Polygonum cuspidatum* was employed in the previous study (Li et al., 2013) and was used here to avoid confusion.

Rosmarinic acid standard (≥98%) was purchased from Sigma-Aldrich Inc. (Darmstadt, Germany), ligustilide and isovitexin standards (98%) from Biopurify Phytochemicals Ltd. (Chengdu, China).

### Preparation of Extracts, Formula and Enriched Formula

Extracts of each single herbal drug and the mixture for the formula were prepared according to the proportions summarized in Table 2 and employed in Li et al. (2013). The powders of single herbal drugs (10 g) were extracted in 250 ml of boiling water and formula (5 g) in 500 ml of boiling water. The extraction was done twice for 1 h in each case. After filtration, these decoctions were freeze-dried. The yields of extraction were of: *A. sinensis* 61.8%, *C. indicum* 36.7%, *G. uralensis* 26.7%, *I. tinctoria* 36.5%, *O. diffusa* 12.2%, *P. cuspidatum* 15.9%, *P. vulgaris* 7.8%, *S. baicalensis* 55.3%, *S. glabra* 22.1%, *S. flavescens*, 28.9% and of 13.7% for the formula (w/w).

The polar compounds of the prepared formula were removed by C18 solid phase extraction (SPE 1,000 mg/12 ml, Finisterre, Teknokroma, Barcelona, Spain). 100 mg of formula solubilized in distilled water were introduced in the cartridge. 10 ml of water was used to obtain an aqueous fraction and 25 ml of methanol (Fisher Scientific, Bishop, United Kingdom) eluted the retained metabolites. After drying, the yields were 65.9% ± 2.8% (w/w) for the aqueous fraction and 17.5% ± 1.2% (w/w) for the methanolic fraction. The loss was 16.5% ± 3.1% (w/w).

For marker purification, a methanolic extract has been prepared to obtain a more enriched extract in secondary metabolites than the decoction. It was obtained from the powdered mixture (200 g) by maceration (eight times 24 h in 5 L) and evaporated, giving a yield of 18.0% (w/w). This methanolic extract was subsequently fractionated into two fractions by Vacuum Liquid Chromatography (VLC) on a RP Zeoprep 60 C18 15–25 µm (Zeochem^®^ Silicagel, Rüti, Switzerland). After equilibration with water, 3 g of extract mixed with the solid phase (1:6) was deposited and eluted with 0.6 L of water and then by 1 L of methanol. The extraction yield was 29.7% (w/w) for the aqueous fraction and 43.4% (w/w) for the methanolic fraction.

### Metabolite Profiling

#### Preliminary Profiles

The formula was solubilized in a solution of water and methanol (7:3), whereas the enriched extract was in water and methanol (2:8), at a concentration of 5 mg/ml for UHPLC-UV-PDA-ELSD and 1 mg/ml for UHPLC-HRMS-TOF. The solutions were sonicated (15 min) and centrifuged (10 min, 6,000 rpm) (Prism R, Labnet international, Inc., Edison, NJ, United States).

Preliminary profiles, were acquired on two independent Waters Acquity UPLC systems (Waters, Milford, MA, United States), the first equipped with a Waters Micromass LCT Premier Time-Of-Flight (TOF) mass spectrometer (Waters) with an electrospray interface (ESI) and the second with UV-PDA and ELSD detections. Chromatographic systems and TOF parameters were set as previously published (Eugster et al., 2014). The second system was controlled by Empower Software v2.0, its UV-PDA acquired from 200 to 500 nm (1.2 nm of resolution) and ELSD Sedex 85 (Sedere LT-ELSD, Alfortville, France) was set at 45°C with a gain of 8. Chromatographic traces were exported from the proprietary format to text files to be plotted on Prism 8 (GraphPad Software, Inc.) (Figure 2).

#### Optimized Profiles

In addition to the formula, freeze-dried extracts of single herbs were prepared as above (Section *Preliminary Profiles*). Optimized profiles were acquired on two independent UHPLC systems, one equipped with three in-line detections (UV-PDA, ELSD and single quadrupole) (UHPLC-UV-PDA-QMS-ELSD) and the second with HRMS/MS.

The chromatographic conditions were the same for both systems: samples were injected (2 µL) into an Acquity UPLC BEH C18 column (1.7 µm, 2.1 mm × 100 mm; Waters) and eluted (0.4 ml/min, 40°C) with water (A) and acetonitrile (B), both containing 0.1% formic acid with the following gradient: from 15 to 75% of B from 0 to 11 min (curve 7), 75–98% from 11 to 12 min, an isocratic step at 98% for 2 min and a re-equilibration step of 2 min.

### Three-Detector System

#### Data Acquisition

A system coupling UV-PDA detection, a single quadrupole mass spectrometer (QMS) and ELSD provided all three detections with the same pump system and thus achieved stably aligned retention times. This UHPLC-UV-PDA-QMS-ELSD system allowed the data from the three detections to be efficiently linked, and in addition, to verify via the QMS the ELSD attributions performed on the UHPLC-HRMS/MS data.

The formula, the enriched formula and the 10 single herb extracts were acquired on this three-detector system. This three-detector system, controlled by MassLynx^®^ V4.2 (Waters), was equipped with an Acquity UPLC system (Waters), which included a binary pumping system, an auto-sampler (set at 10°C), a column manager with a pre-column heater (set at 40°C), a PDA detector and an isocratic solvent manager which directed 10% of the flow to the single quadrupole (Acquity QDA, Waters) while adding a flow of 200 μL/min of water-acetonitrile (1:1) containing 0.1% formic acid. The remaining 90% of the flow was directed to an ELSD (Büchi ELS Detector C-650), set at 45°C, gain 8. The QDA, equipped with an ESI source, was set as follows in both modes: probe temperature 600°C, ESI capillary voltage 1.2 kV, cone voltage 15 V, source temperature 120°C, acquisition range 30–1,250 Da. In this setting, the greater sensitivity of the simple quadrupole allows to obtain relevant signals on the 10% of the flow coming from the column. The isocratic pump increases the flow to ensure adequate ionization by the ESI.

#### Evaporative Light Scattering Detection Data Treatment

The ELSD traces of each herb were integrated with MassLynx^®^ V4.2 (Waters) and only ELSD areas equal to or greater than 0.004 μV/s were considered. A table containing UV max and MS data was prepared in order to be compared with the HRMS data presented below. To visually compare the 10 herbs in an informative graphical representation, ELSD areas of each herb were represented using Prism software as a bar plot in a superimposed manner, each color representing an herb (Figures 5C,D and Supplementary Figures 2C,D). Two area scales were chosen to illustrate the herbs with the most intense peaks (area from 0.0 to 0.4 μV/s) (*G. uralensis*, *P. cuspidatum*, *S. flavescens*, and *S. baicalensis* (Figure 5C) and the herbs with less intense peaks (areas from 0.00 to 0.008 μV/s (Figure 5D) (*C. indicum*, *I. tinctoria*, *O. diffusa*, *P. vulgaris*, *S. glabra*). The peak areas were labeled by a letter according to their source herb and by a number in order of importance of their area, from the largest to the smallest. A total of 47 ELSD peaks were detected. The numbers of ELSD peaks for each herb were of seven for *C. indicum*, nine for *G. uralensis*, two for *I. tinctoria*, eight for *O. diffusa*, four for *P. cuspidatum*, five for *P. vulgaris,* one for *S. glabra*, ten for *S. baicalensis*, and one for *S. flavescens*. No ELSD peak was detected for *A. sinensis*. Peaks not retained under RP chromatographic conditions were not considered.

### High Resolution Spectrometry Analysis

The analyses were performed on an Acquity UPLC system interfaced to an Orbitrap Q-Exactive Focus mass spectrometer (Thermo Scientific) using a heated electrospray ionization source (HESI-II) and an Acquity UPLC PDA detector. Thermo Scientific Xcalibur 2.1 software was employed for instrument control. The detailed conditions are presented in Supplementary Section S9.

#### High Resolution Spectra Data Processing

*ThermoRAW* MS data were converted to .*mzXML* using ProteoWizzard (Kessner et al., 2008) and loaded to MZmine 2.37 (Pluskal et al., 2010; Pluskal et al., 2012). To prepare the peak lists, the ADAP workflow was employed (Myers et al., 2017), followed by a deisotoping step and an alignment step (Supplementary Section S10 for the precise parameters). The parameters were based on those previously used in our laboratory for single herb profiling (Barthelemy et al., 2019; Rutz et al., 2019), and no specific adaptation to a multi-herb extract has been made. Finally, the peak lists were filtered to keep only peaks with HRMS/MS scan. The HRMS level, which contained the peak height and retention time data of each feature was exported to a text file, whereas the HRMS/MS level was exported as an .*mgf* file to be submitted to the online workflow at GNPS (Wang et al., 2016a).

#### Feature-Based Molecular Network

Molecular network (MN) were created where edges were filtered to have a cosine score above 0.7 and more than six matched peaks. Further edges between two nodes were kept in the network if and only if each of the nodes appeared in each other’s respective top 50 most similar nodes. Tolerances for precursor and fragment ions were set at 0.02 Da. The spectra in the network were then searched against GNPS spectral libraries. All matches kept between network spectra and library spectra were required to have a score above 0.7 and at least six matched peaks. The GNPS job parameters are available at https://gnps.ucsd.edu/ProteoSAFe/status.jsp?task=4cffb19303354a6a814184facb930bc0 (PI) and https://gnps.ucsd.edu/ProteoSAFe/status.jsp?task=b949d909e6484ed6b6b28db4c09109ac (NI).

The next step was to compare the experimental spectra organized in the MN against an in-house *in silico Database* (ISDB-DNP) prepared from the Dictionary of the Natural Products (DNP, 2019), according to a previously reported workflow (Allard et al., 2016). To do this, the *.mgf* file from GNPS and the *.tsv* file containing the database matches from GNPS were used. A top six consultation was employed for annotating the peaks at a tolerance of 0.005 Da and a cosinus score threshold of 0.2. FBMNs were visualized with Cytoscape 3.7.0 (Figure 3B and Supplementary Figure S1B), and annotations were displayed thanks to the Chem Viz2 1.1.0. Tables from HRMS data, GNPS library and ISDB-DNP consultations were loaded in Cytoscape.

#### Node and Cluster Specificity

The table gathering all information in Cytoscape was then re-exported in a text file to be evaluated by an in-house Jupyter Notebook script in terms of nodes and clusters herb-specificity (Supplementary Figure S4). The Jupyter Notebook and the corresponding python script are available on gitlab: https://gitlab.unige.ch/Arnaud.Gaudry/node_treater. The data loaded into the script combined the MS data obtained after the alignment of the features (called the quantification table in GNPS terminology, which contained for each aligned feature its *m*/*z*, retention time and peak height(s)), with the information related to clusters (cluster numbers, called *component indexes* in GNPS terminology). First, at the node level, the relative intensity of each feature detected in the 10 herbs was calculated, allowing the description of each node in terms of contribution of each herb, referred to as a *specificity percentage*. The lists of aligned features were then reduced to features detected in the formula (deletion of the features detected in herbs but not in the formula, as well as in the blank analyses). A threshold of 90% was applied to retrieve the specific features in an individual herb extract. The number of features specific to one herb was then counted. At the cluster level, the averages of these specificity percentages were calculated, allowing the description of each cluster according to the contribution of the 10 herbs. This percentage was named the *cluster specificity percentage*. Furthermore, this in-house script could export the HRMS data (*m*/*z*, retention time and peak height) for a specific cluster, to facilitate the link between the FBMN and the metabolite profiles. The HRMS data exported by this script were then represented against the chromatogram in the form of a bar plot with Prism 8 software (Figures 7, 8 and Supplementary Section S8), referred to as *bar chromatograms*. For the clarity of some bar chromatograms, features with the same retention time within a given cluster were deleted to avoid representing in-source features (adducts, complexes and/or fragments).

#### Visualization in the Feature-Based Molecular Network

To visualize the contribution of each herb to the formula, the height values of the deconvoluted peaks were represented on the nodes in two layers (legend Figure 4). In the center of the node, a pie chart represented the formula (pale pink) and the blank analyses (white). On the external part of the node, an external ring represented the peak heights of the feature in the single herb extracts. The node size mapped the feature heights in the formula.

#### 2D Feature Maps

The aligned table obtained previously thanks to the in-house script was exported to a *.csv* file and loaded in Microsoft Excel 16.16.8. To prepare the 2D feature map, the feature height intensities in the formula have been assigned to the features specifically detected in one herb. Features detected below the threshold of 90% were not attributed to a given herb. This feature list was plotted as a scatter plot (arbitrary plot size for Figure 3A and proportional to peak height intensity in Figure 5A in PI; Supplementary Section S1 for NI). Features found in the formula were plotted as black circles, whereas aligned features from each herb were plotted as dots according to a color code representing each herb. Thus, a specific aligned feature was visible as a black circle surrounding a colored dot. Finally, ELSD peak areas from the single herb extract (Sections *Evaporative Light Scattering Detection data Treatment* and *Annotation Strategy*) were manually assigned to the specific features in an additional column in the aligned table. After this manual assignment, the same plot was prepared with the attributed ELSD areas (Figure 5E).

#### Annotation Strategy

The annotation strategy combined ELSD filtering, interpretation of MS, HRMS, HRMS/MS and UV-PDA spectra with chemotaxonomy information. ELSD filtering retrieved 47 peaks (Section *Evaporative Light Scattering Detection Data Treatment*). The MS data were interpreted in parallel in both modes. First, in regard to ELSD peaks, the corresponding nominal *m*/*z* were retrieved in the QMS traces (Section *Three Detector System*). These *m*/*z* were then searched in the peak lists obtained by processing the UHPLC-HRMS/MS metabolite profiles (Section *Data Acquisition*). The specificity of the peaks highlighted by ELSD was verified. The molecular formula of the specific peaks was then calculated and searched in the DNP (DNP, 2019). The number of potential structures were recovered for the species, genus, family, if previously described. These taxonomic data were then compared with the annotation obtained on HRMS/MS data by consultation of the theoretical *in silico* MS/MS spectral database (Section *Feature-Based Molecular Network*). This annotation workflow is presented with more details in Supplementary Section S5. The annotation results are summarized in Supplementary Tables S2–S11 and all associated metadata are available in the Cytoscape files in doi:10.25345/C5516P including IUPAC name and InChI key.

#### Isolation of the Markers

In order to rapidly isolate the makers, a fractionation at high-scale on Medium Liquid Chromatography (MPLC) was implemented. A gradient optimization was performed at High Pressure Liquid Chromatography (HPLC) scale before its transfer to MPLC (Büchi). The enriched extract was separated thanks to a HPLC system (Agilent Technologies 1260 Infinity), controlled with the software Chemstation for LC3D (Agilent Technologies), equipped with UV-PDA detection and ELSD (Sedex LT-ELSD 85 (Sedere, Oliver, France)). A Zeoprep^®^ column (A60 C18, 250 mm × 4.6 mm i.d., 15–25 mm, Zeochem) was used at a flow of 1 ml/min of water (A) and methanol (B) both with 0.1% of formic acid (Fisher Scientific, Bishop, United Kingdom). The optimized gradient was from 20 to 73% of B in 60 min, and then 73–100% in 20 min. Gradient transfer calculation (Challal et al., 2015) indicated the following MPLC conditions: 37 min of isocratic step at 20% of B, 747 min from 20 to 73% and 257 min from 73 to 100%, for a total of 19h30 of separation at a flow of 15 ml/min. The MPLC column (460 mm × 49 mm i.d.) was packed with the same solid phase as at HPLC scale. The enriched extract (7 g) (Section *Preparation of Extracts, Formula and Enriched Formula*) was introduced in MPLC by a dry load cell (Challal et al., 2015) and fractionated into 50 fractions. In order to monitor the MS features associated to the marker highlighted by the annotation strategy of the formula, all fractions and the enriched extract were checked in short chromatographic conditions with the same systems used for preliminary profiling (Section *Preliminary Profiles*), on one hand with UHPLC-UV-PDA-ELSD detection and on the other by UHPLC-HRMS-TOF, set as described in (Brillatz et al., 2018). HRMS data were converted to .*cdf* format through Databridge provided by MassLynx and loaded to MZmine. An aligned peak list was prepared as previously described, with specific parameters summarized in Supplementary Section S10. Annotated markers (Supplementary Tables S2–S11) were searched in the aligned table through their *m*/*z* in order to detect in which fraction(s) they were located. The ELSD traces of each fraction of interest were evaluated to verify the purity of the markers prior NMR analysis. Thus, seven targeted markers were identified directly in the MPLC fractions. Fraction M21 (82 mg) contained C2, fraction M34 (77 mg) and M35 (44 mg) contained mainly G1, fraction M31 (83 mg) mainly PO2, fraction 13 (199 mg) mainly SC1, fraction M25 (71 mg) mainly SC2, fraction M17 (83 mg) contained SM1. The ^1^H and ^13^C-NMR signal assignments are presented in Supplementary Section S5. The MS/MS spectra corresponding to these components in both ionization modes have been added to the GNPS library to be shared with the community.

### Semi-Preparative Purification

To isolate targeted compounds that were not obtained in sufficient purity in the MPLC fractions, semi-preparatory purifications were performed from the MPLC fractions. First the chromatographic conditions were optimized at the HPLC analytical scale in the same system described in Section *Isolation of the Markers*, on an X-bridge C18 column (250 mm × 4.6 mm i.d., 5 µm) (Waters) equipped with a pre-column cartridge holder (20 × 4.6 i.d., Waters). The flow was set at 1 ml/min of water and methanol both with 0.1% of formic acid. The optimized LC conditions at the analytical level were geometrically transferred to semi-preparative scale (Guillarme et al., 2007; Guillarme et al., 2008; Challal et al., 2015). Semi-preparative HPLC was performed on an ARMEN Spot Prep System (Saint-Avé, France), with an X-bridge Prep C18 OBD column (250 mm × 19 mm i.d., 5 µm) (Waters) equipped with a UV detector and an ELSD Sedex 55 (Sedere). The flow rate was set at 17 ml/min. Fraction M6 yielded C1 (1.1 mg) (30% of methanol), fraction M23 yielded C4 (2.3 mg) and SC3 (1.0 mg) (40% of methanol). Fraction M14 yielded SO1 (2.9 mg) (1% of methanol) and PO1 (1.5 mg) (gradient from 2 to 50% of methanol in 30 min). Fraction M20 yielded O1 (8.5 mg) and SC1 (8.1 mg) (gradient from 45 to 75% of methanol in 30 min). Fraction M15 yielded G2 (8.3 mg) (gradient from 10 to 100% of methanol in 30 min). The ^1^H and ^13^C-NMR signal assignments are presented in Supplementary Section S5.

### Spiking Experiments

To confirm the annotations of **A1**, **I1** and **PR1**, the retention time and spectra of the standards were compared with those observed in the corresponding herb extracts (*A. sinensis*, *I. tinctoria* and *P. vulgaris*) with the methods described in Sections *Optimized Profiles* and *Data Acquisition* (UHPLC-UV-PDA-QMS-ELSD). Spiking experiments were also performed (data not shown).

### Characterization of Isolated Constituents

NMR spectra were recorded on a Bruker Avance III HD 600 MHz NMR spectrometer equipped with a CQI 5 mm Cryoprobe and a SampleJet automated sample changer (Bruker BioSpin). Chemical shift were reported in parts per million (δ) using the deuterated dimethyl sulfoxide (DMSO-d6) signal (δ_h_ 2.50; δ_c_ 39.5) as internal standards for ^1^H and ^13^C NMR, respectively, and coupling constants (J) were reported in hertz. Assignments were obtained based on two-dimensional (2D) NMR experiments (COSY, NOESY, HSQC and HMBC) (Supplementary Section S7 for the precise descriptions).

## Conclusion

This untargeted exploratory study provided a comprehensive characterization of the chemical composition of a complex TCM formula to study the specific contribution of each herb to the formula and to select quality control markers. It exemplified how metabolomics data can help to perceive the chemical contribution of each single herb to the final formula. The data-driven workflow proposed in this study for a given formula is innovative and could be used to investigate other TCM formulae in view of selecting specific markers and sub-markers. As discussed, the proposed method is certainly not to be used for a regular routine control, but it shows how far herb contribution can be monitored with state-of-the-art method in the field. Such analysis produces highly informative compositional data on representative batches, that can be used as a reference for designing appropriate and simpler quality control methods.

The extensive use of FBMN for quality control purposes needs to be evaluated in more detail. For this, methods should be developed to compare formulae, to evaluate and quantify their composition consistency in an automated and generic manner. This mainly required dedicated software development since data acquisition is ensured by most state-of-the-art available platforms. With the rapid spread of molecular networking in many fields of plant analysis, integration of high-quality data with innovative automated methods and comprehensive natural products databases is expected and might be adopted in future by authorities and TCM producers.

## Data Availability Statement

The datasets presented in this study can be found in online repositories. The names of the repository/repositories and accession number(s) can be found below: https://massive.ucsd.edu/ProteoSAFe/dataset.jsp?task=a8219bbd768944e99257486adc672003, MassIVE MSV000085648.

## Author Contributions

JH designed and conceived the study, performed the experiments and interpreted the data, wrote the manuscript and created the figures. P-MA designed the HRMS/MS data interpretation and revised the manuscript. EQ designed the fractionation experiments and revised the manuscript. LM interpreted the NMR analyses of all isolated compounds. AG created the Jupyter notebook script to interpret HRMS and HRMS/MS data and revised the manuscript. LV performed part of the experiments of extraction and isolation. SL and RW developed the multi-herb formula. KK provided the multi-herb formula, was consulted during study design and revised the manuscript. J-LW conceived and designed the study, the experiments and the figures, wrote and revised the manuscript.

## Conflict of Interest

The authors declare that the research was conducted in the absence of any commercial or financial relationships that could be construed as a potential conflict of interest.
