# Supplementary material for: A Mass Spectrometry Based Metabolite Profiling Workflow for Selecting Abundant Specific Markers and Their Structurally Related Multi-Component Signatures in Traditional Chinese Medicine Multi‐Herb Formulae
Source: Front Pharmacol. 2020 Dec 3;11:578346. doi: 10.3389/fphar.2020.578346 (PMC7756971; doi:10.3389/fphar.2020.578346)
Supplement: Supplementary file 1 [file datasheet1.pdf]

Supplementary Material

1 *Visualization of the contribution of each herb to the formula and ELSD filtering* 2

2 *Script to evaluate the specificity of nodes and clusters* 5

3 *Features and clusters specificity* 7

4 *ELSD chromatograms of the TCM formula and individual herb extracts* 9

5 *Detailed annotations of the specific and abundant components* 11

6 *Identification and annotation tables* 17

7 *Description of isolated components* 30

8 *Multi-component signatures* 34

9 *UHPLC-UV-PDA HRMS/MS data acquisition* 39

10 *MZmine parameters for peak picking* 40

11 *References* 42

1 Visualization of the contribution of each herb to the formula and ELSD filtering

These are additional figures for sections 2.2. and 2.3.

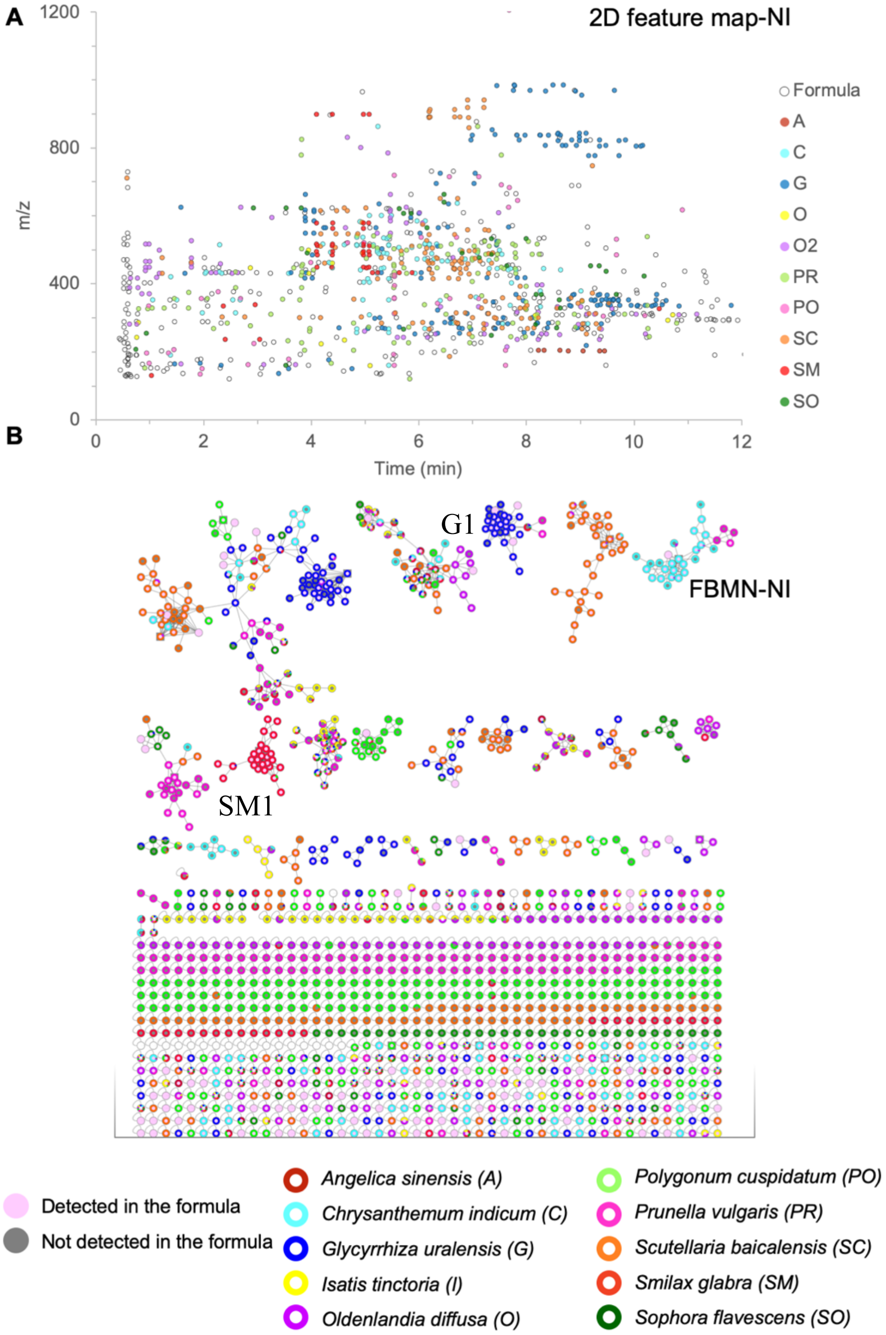

Supplementary Figure 1: Visualization of the contribution of the ten herbs to the formula, in which each herb is represented by a different color: A) 2D feature map in NI: each black circle represents a feature detected in the formula, the size of the circles is fixed and equal for all features. The inner color of the circle indicates that the feature is specifically detected in one of the ten herbs (90% specificity threshold). B) FBMN in NI for the organization of the MS/MS spectra of all features presented in A, which the same color coding and fixed node size.

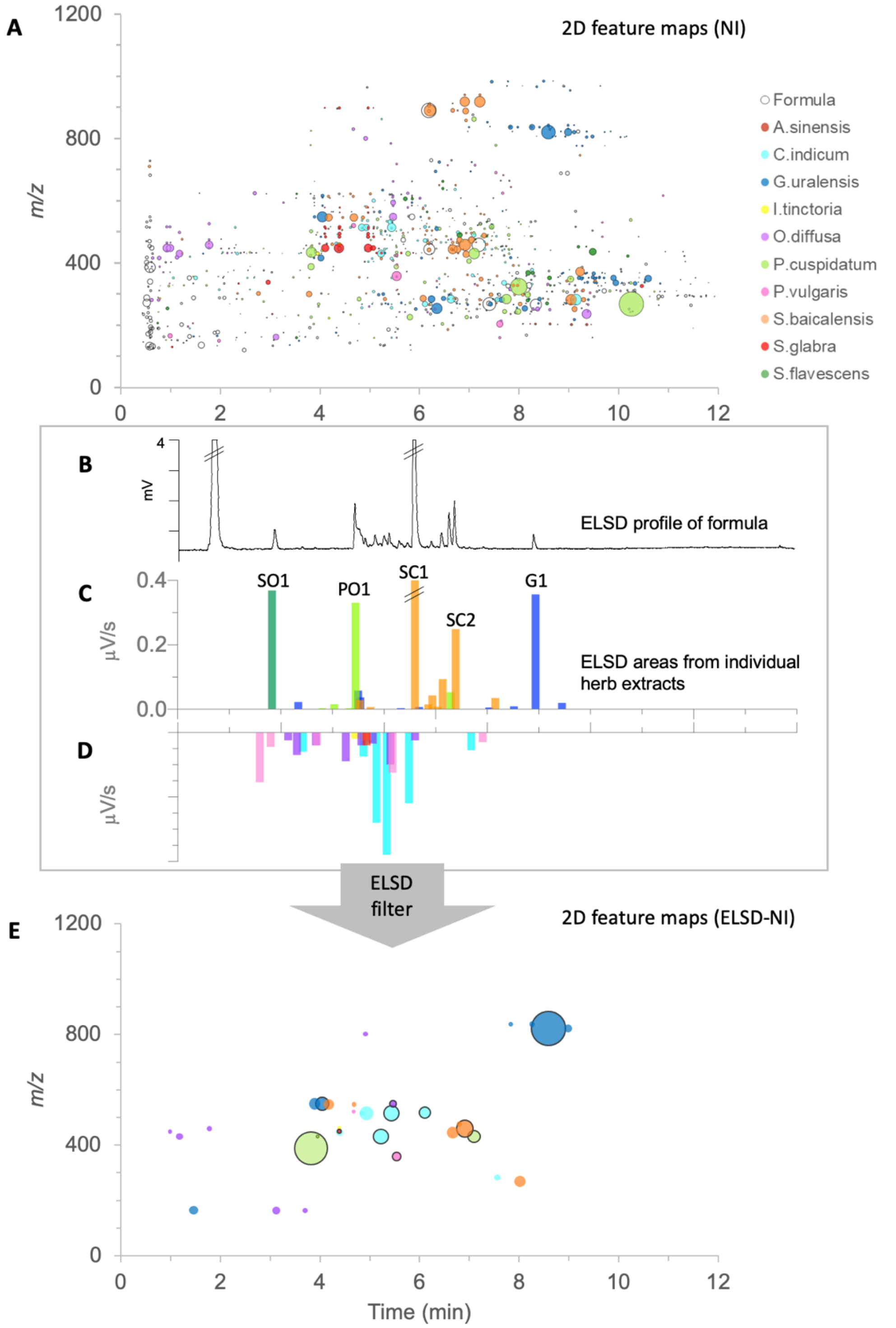

Supplementary Figure 2: A) 2D feature map of HRMS processed data in negative ionization, B) ELSD profiling of the formula, C) and D) bar plot with superimposed ELSD area of the single herb extracts adjusted to their proportion into the formula, divided into high (C) and low areas (D). E) 2D feature map (NI) with attributed ELSD areas. Dots with black circle represents the components that were further identified.

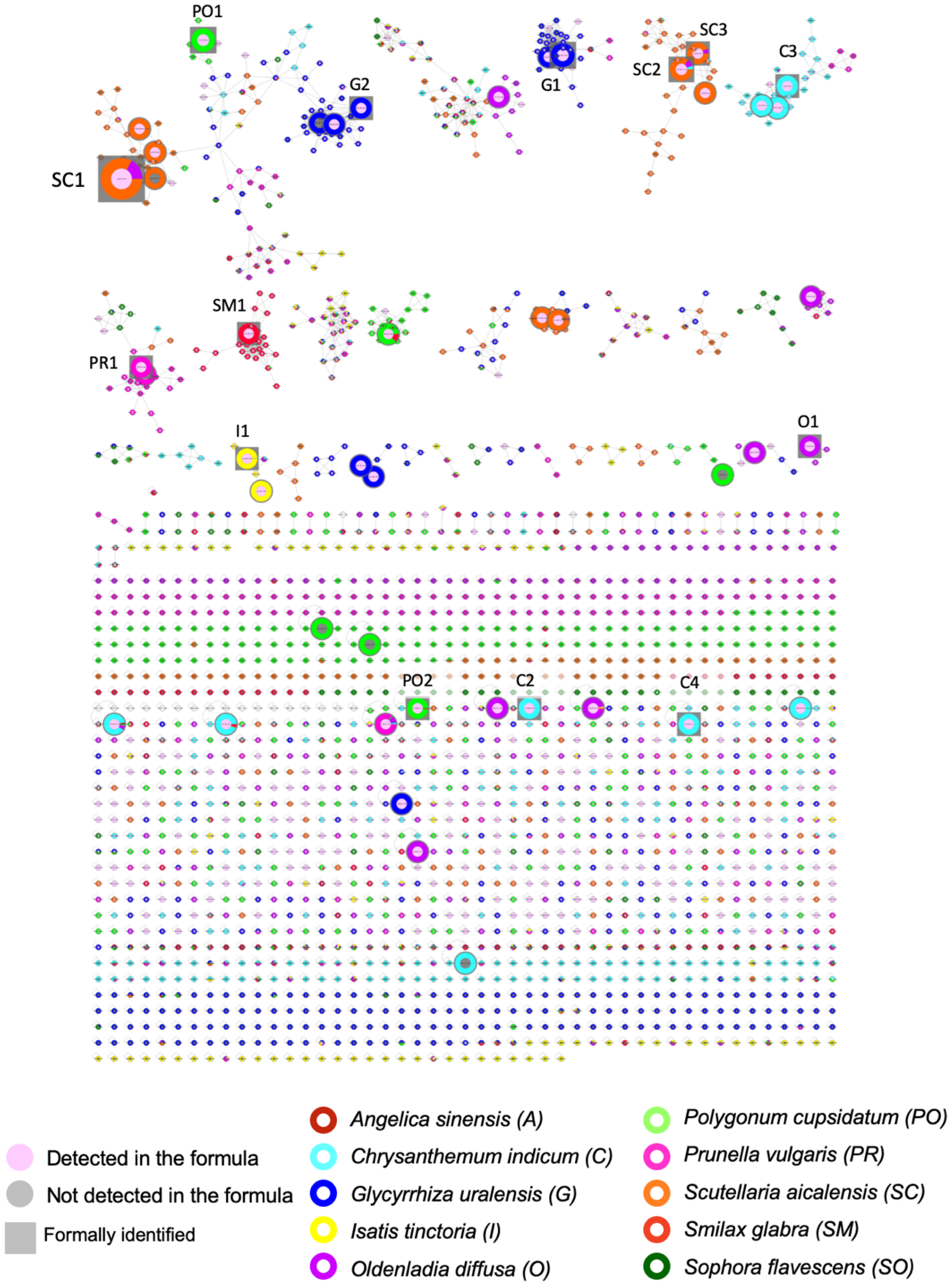

Supplementary Figure 3: Visualization of the ELSD detected peaks in the FBMN-NI. Each herb is represented by a different color, the size of the node is proportional to ELSD areas. See table 2 for the name of the components.

2 Script to evaluate the specificity of nodes and clusters

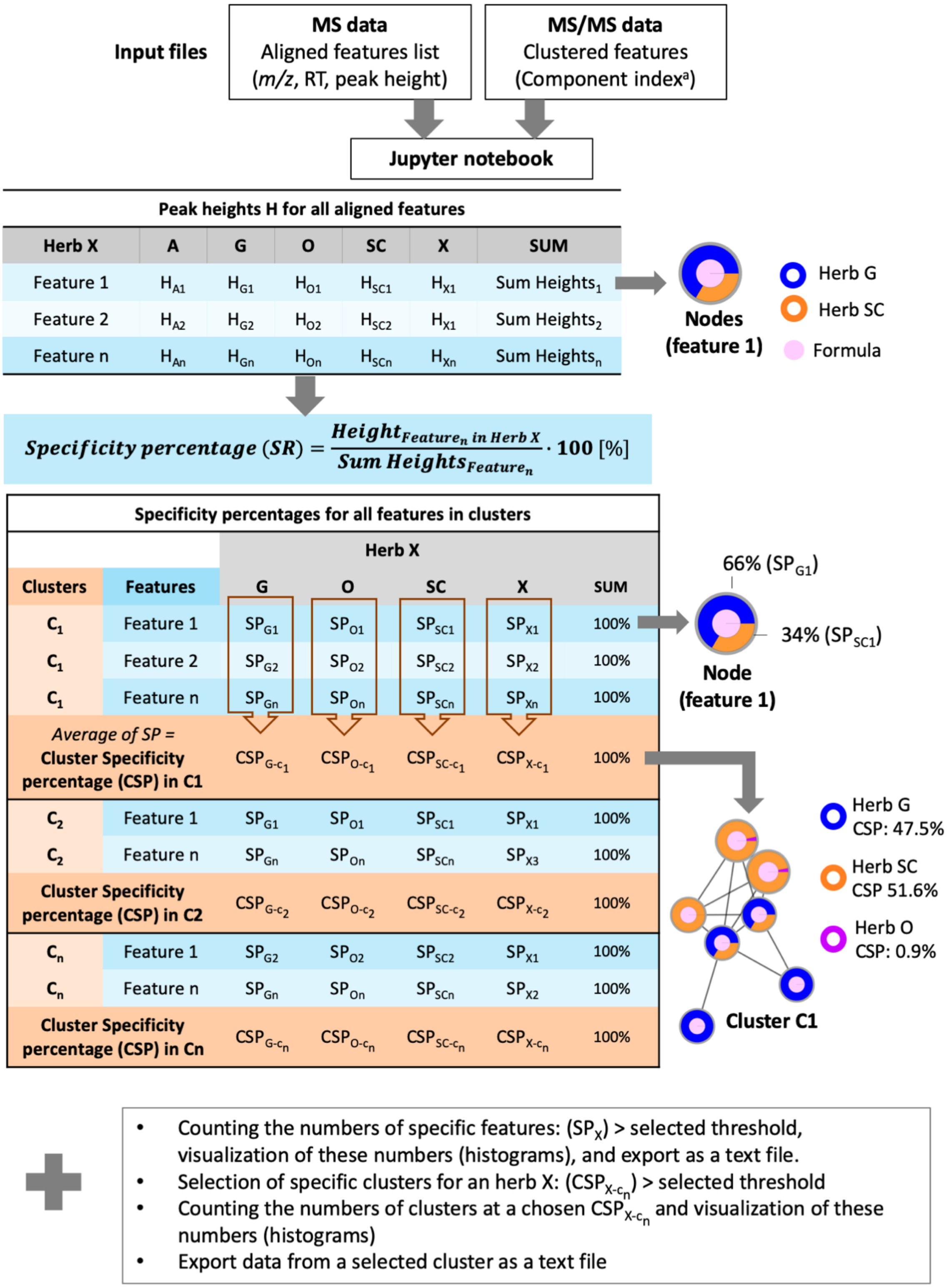

Supplementary Figure 4: Main steps of the script developed to express numerically the specificity of nodes and clusters. The *specificity percentage* is the relative height intensity of one feature in a multi-herb formula. The peak height of one aligned feature in one herb is divided by the sum of the heights in all herbs. The *cluster specificity percentage* is the average of the specificity percentages of the features grouped in a cluster for a given herb. [a] component index is a number that designates a cluster in GNPS terminology

3 Features and clusters specificity

These are additional figures for section 2.2. and 2.3

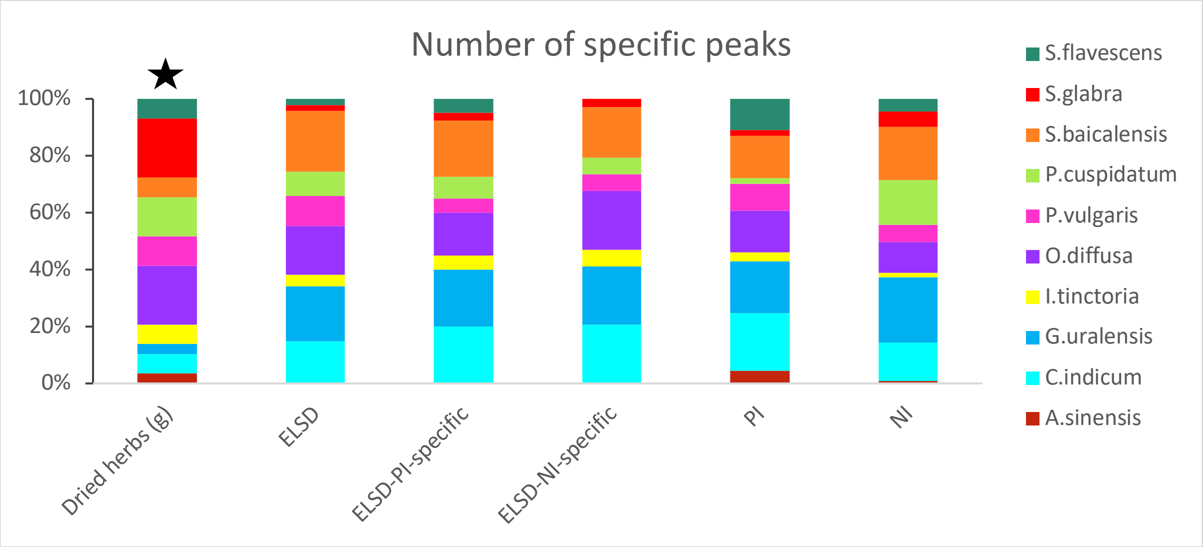

Supplementary Figure 5: Comparison of the numbers of specific peaks (90% threshold) in regard of the herbal drug proportion (★) in the formula before decoction. PI: positive ionization. NI: negative ionization.

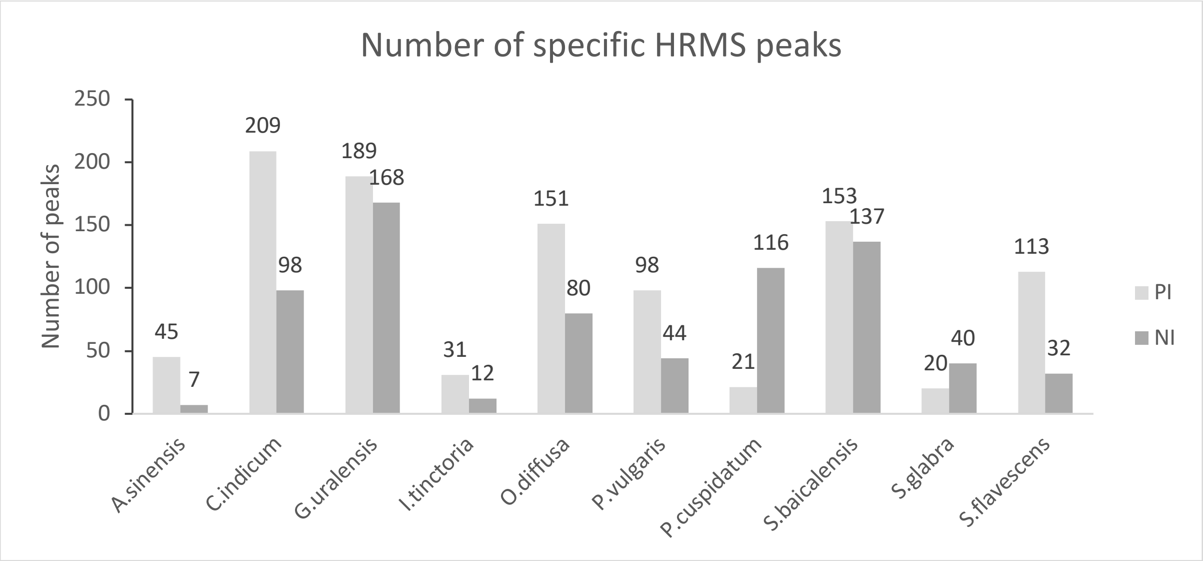

Supplementary Figure 6: Comparison of the numbers of specific peaks (90% threshold) for each herb in UHPLC-HRMS. PI: positive ionization. NI: negative ionization.

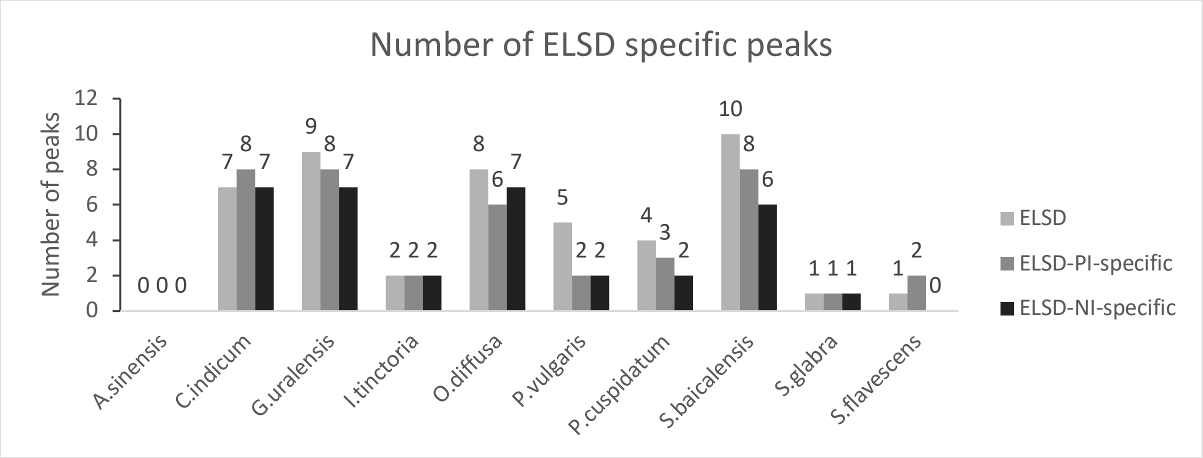

Supplementary Figure 7: Comparison of the numbers of specific peaks ELSD detected for each herb. PI: positive ionization. NI: negative ionization.

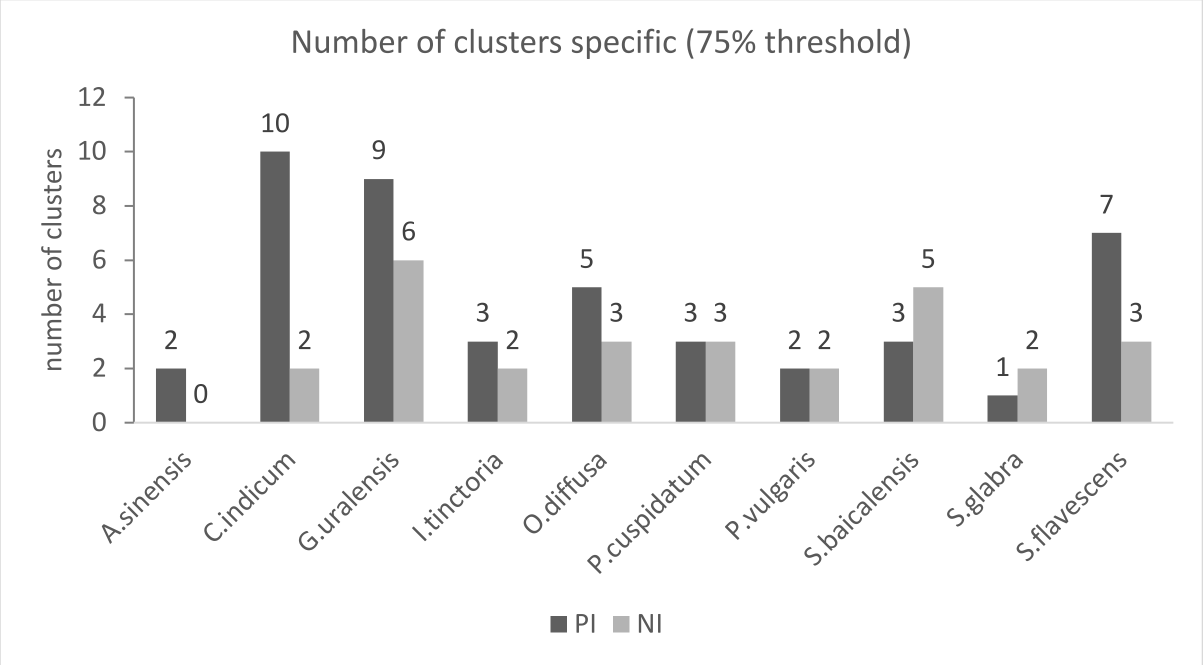

Supplementary Figure 8: number of specific clusters to each herb in both ionization mode in the FBMN (75% threshold).

4 ELSD chromatograms of the TCM formula and individual herb extracts

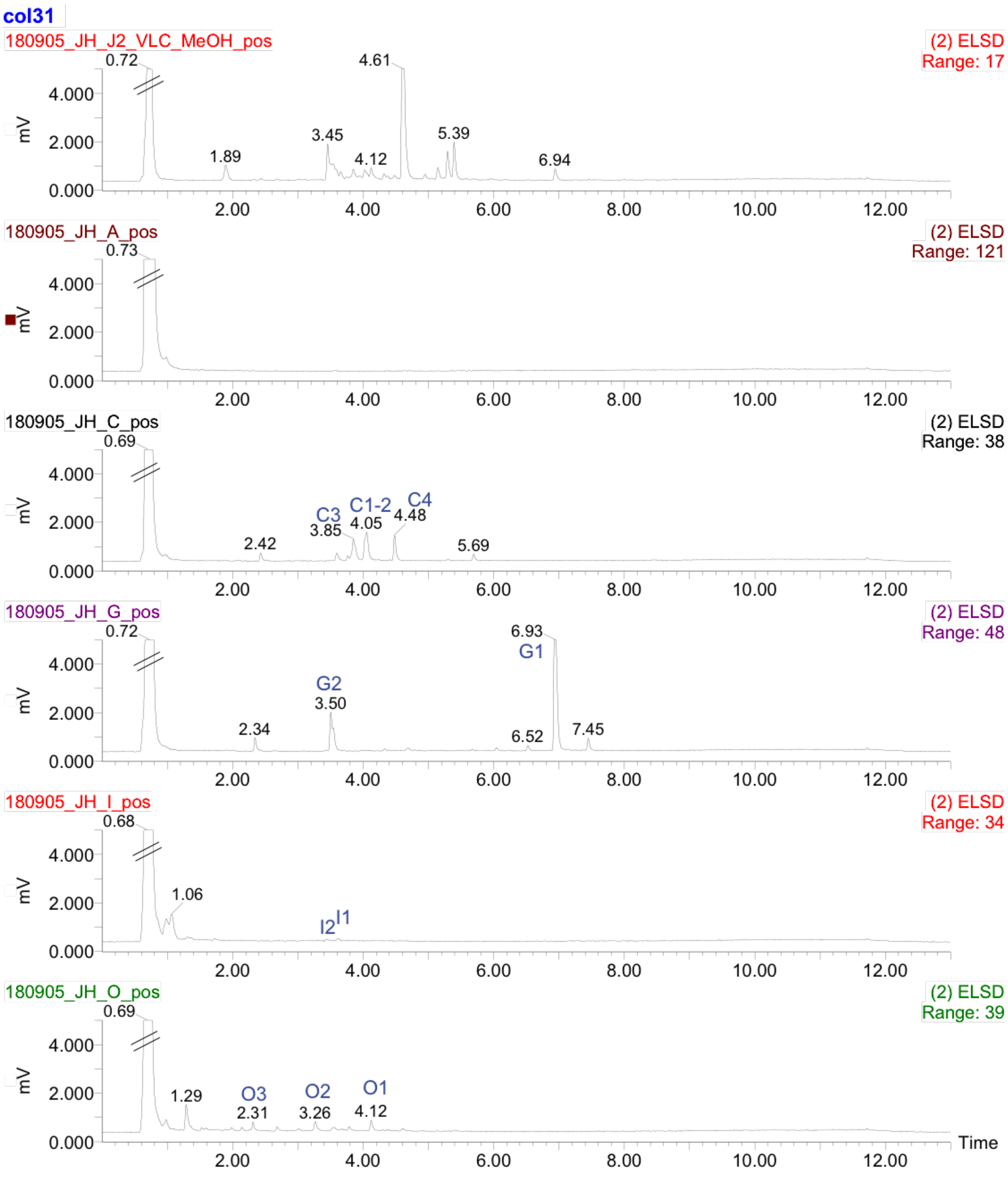

Supplementary Figure 9: ELSD chromatograms of the enriched extract (J2-VLC-MeOH) and single herb extract (*A.sinensis* (A), *C. indicum* (C), *G. uralensis* (G), *I.tinctoria* (I), *O.diffusa* (O)) with ELSD peak numbering corresponding to subsequent annotations and identifications.

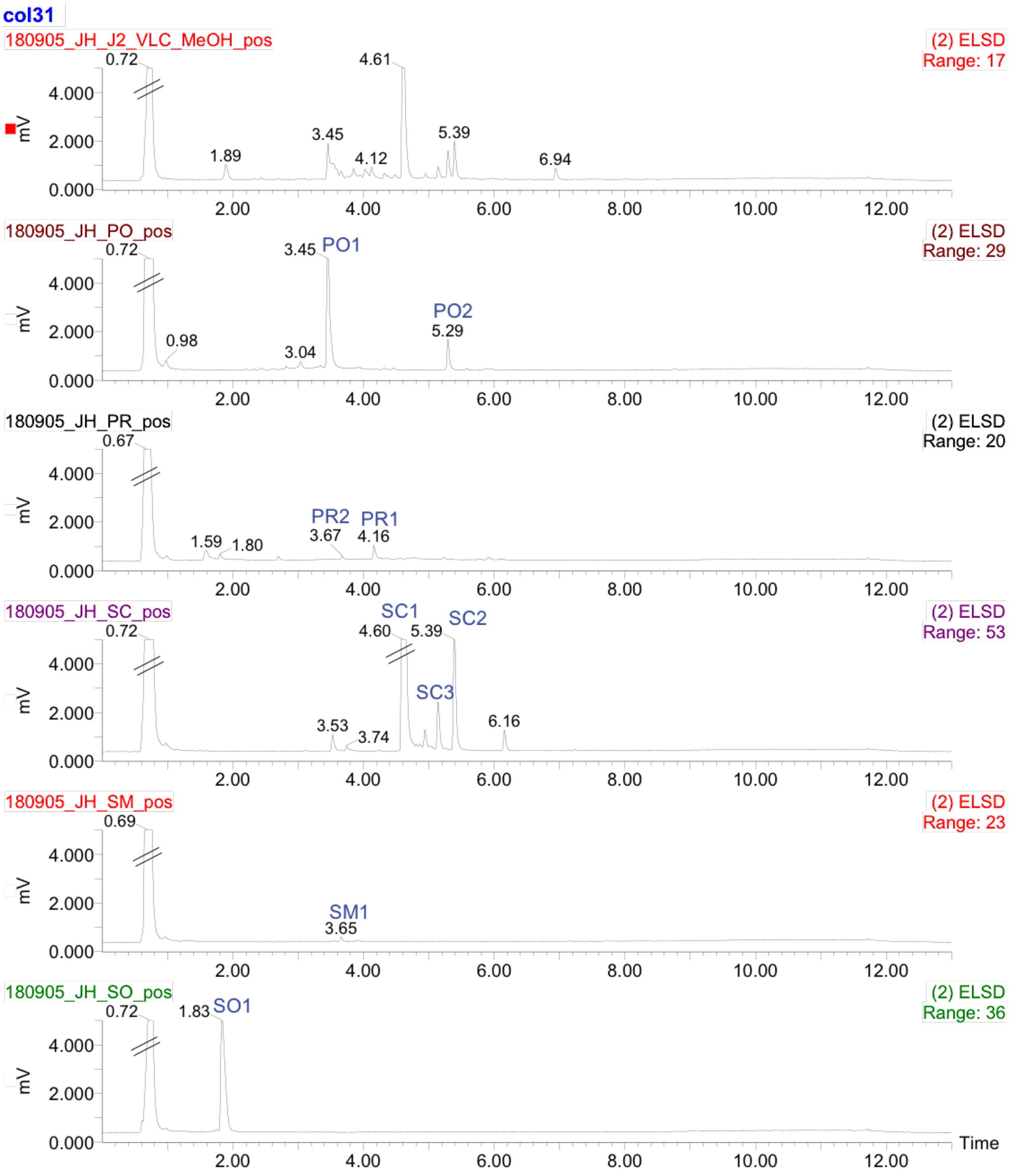

Supplementary Figure 10: ELSD chromatograms of the enriched extract (J2-VLC-MeOH) and single herb extract (*P.cuspidatum* (PO), *P.vulgaris* (PR), *S.baicalensis* (SC), *S.glabra* (SM), *S.flavescens* (SO)), with ELSD peak numbering corresponding to subsequent annotations and identifications.

5 Detailed annotations of the specific and abundant components

5.1 Annotation workflow

For the features selected by ELSD filtering, their ISDB annotations obtained in the FBMN were verified and completed in detail. The annotation strategy combined MF assignment with taxonomic data since most of the constituting herbs were rather well documented from a phytochemical point of view. HRMS/MS and UV spectra were used for checking annotation consistency (Main text Fig. 1.6 and Suppl. Mat. Fig.5). The number of potential structures for the MF retrieved from HRMS data were reduced by applying a taxonomic filter (from the species to the family taxa level) (Suppl. Mat. Fig. 5). The annotation consistency was checked between the hits obtained by MF assignment from HRMS and filtering by taxonomy and ISDB spectral scoring (Top 6 hits) (Suppl. Mat. Fig. 5). When necessary, UV spectra were used as orthogonal information to confirm or discriminate the classes of compounds. Finally, a comparison with the markers referenced in the European and Chinese Pharmacopoeias was performed. Three detailed examples of this annotation process are presented in Suppl. Mat. Fig. 6 to 8. The annotation presented below were obtained from a first batch of metabolites profiling (data not shown). This workflow resulted in the thorough annotation of 22 potential markers among the features selected by ELSD, which included between 2 to 4 potential markers per herb, at the exception of *Angelica sinensis* for which no ELSD peak were detected. The combination of MF assignment, taxonomic filter, MS/MS scoring and orthogonal UV check provided in half of the cases a single highly probable structure, and in the other half up to 4 putative structures belonging to the same chemical class of components (Suppl. Mat., Table S1). Interestingly, the results of the taxonomic filtering and ISDB annotations were consistent in 80% of cases. Furthermore, orthogonal control by UV spectra permitted the discrimination of all annotated structures belonging to more than one class of compounds even after taxonomic filtering. Finally, the verification of the annotations against the Pharmacopoeias permitted to discriminate between 2 annotations and to modify a case.

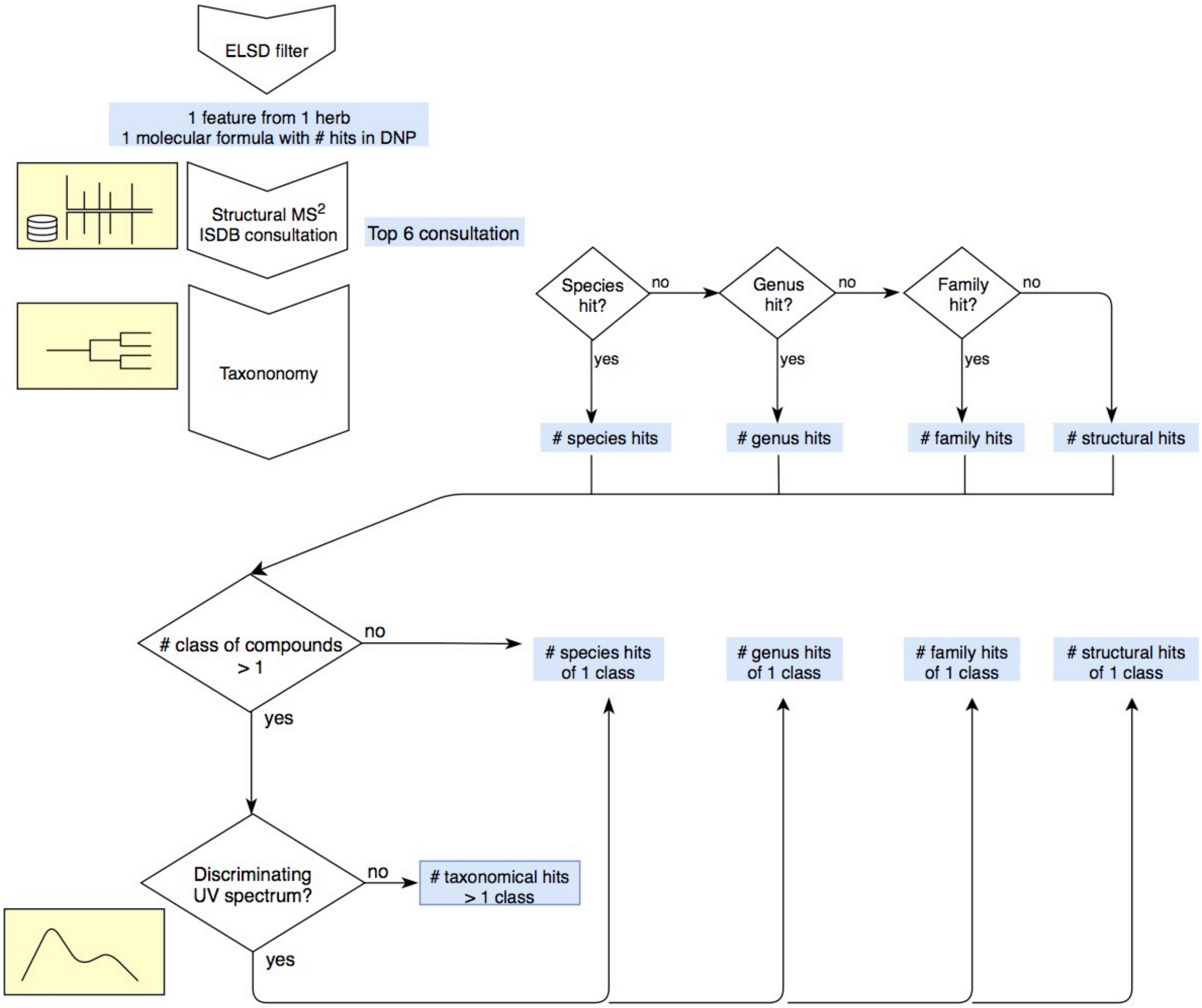

Supplementary Figure 11: scheme of the annotation strategy.

5.2 Example of annotations

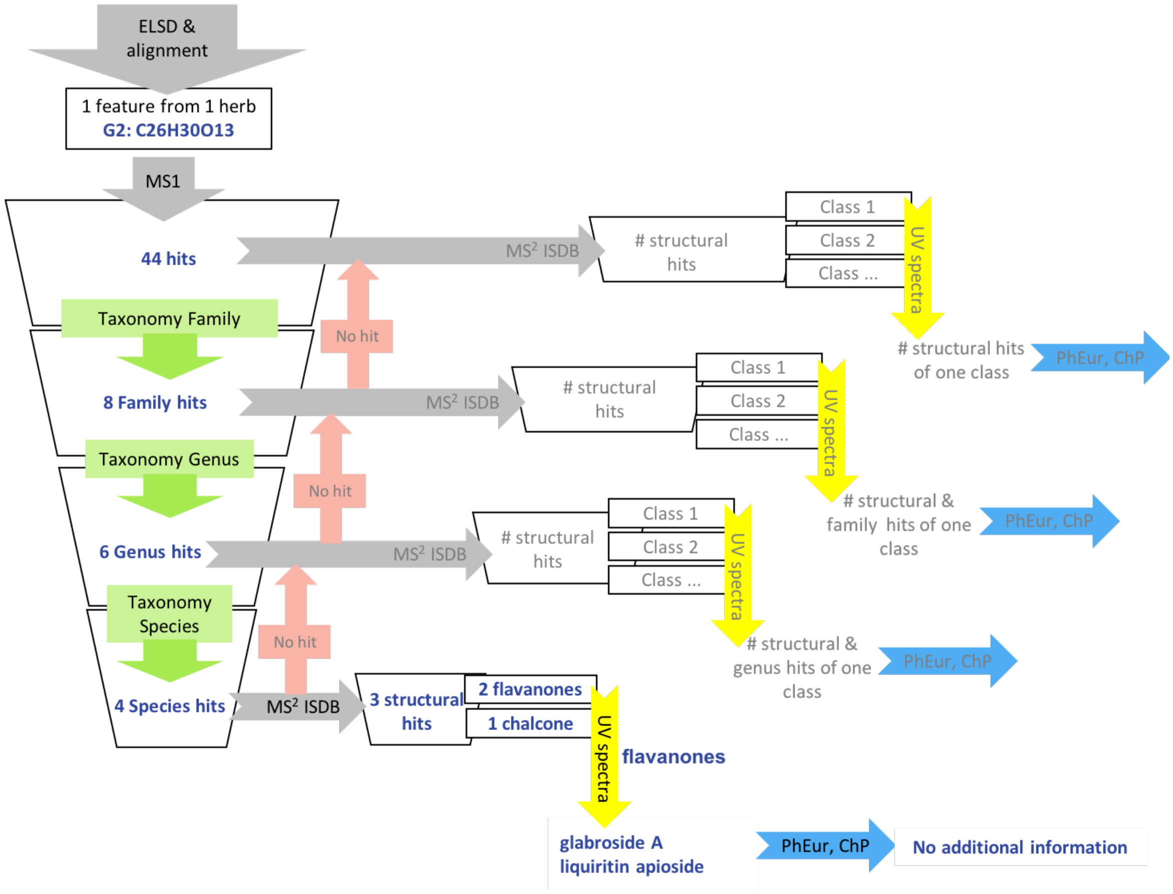

Supplementary Figure 12: annotation scheme for G2, (formally identified as liquiritin apioside): for G2 molecular formula, the DNP listed 4 possible structures for the species, three of which were proposed by the ISDB consultation. Among them, the UV spectrum eliminated the chalcone, resulting in two possible structures of the same chemical class at the end of the annotation.

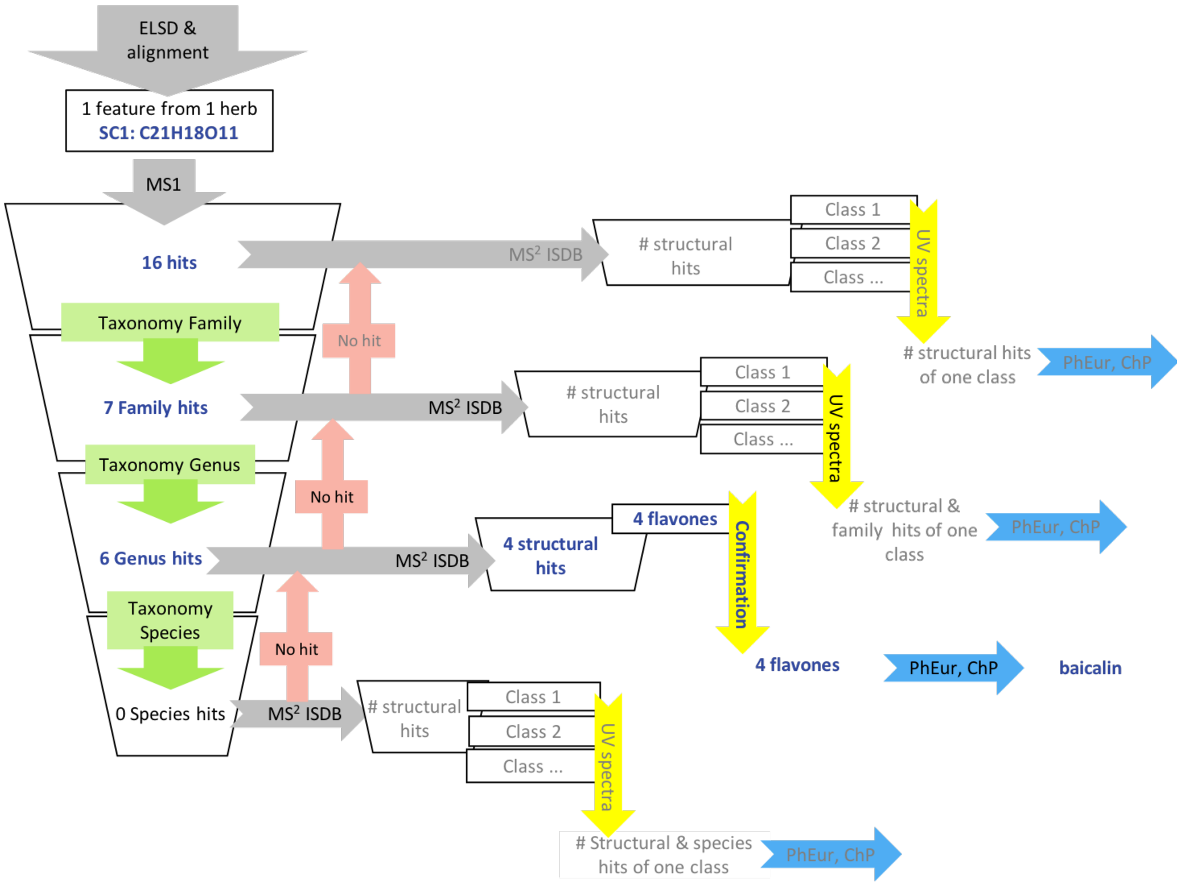

Supplementary Figure 13: annotation scheme for SC1 (baicalin): this component was classified as a trihydroxyflavone by taxonomy (6 genus hits), four of which were then proposed by ISDB consultation. Among the 4 final hits, the most relevant was baicalin, which is the marker of both European and Chinese Pharmacopoeias.

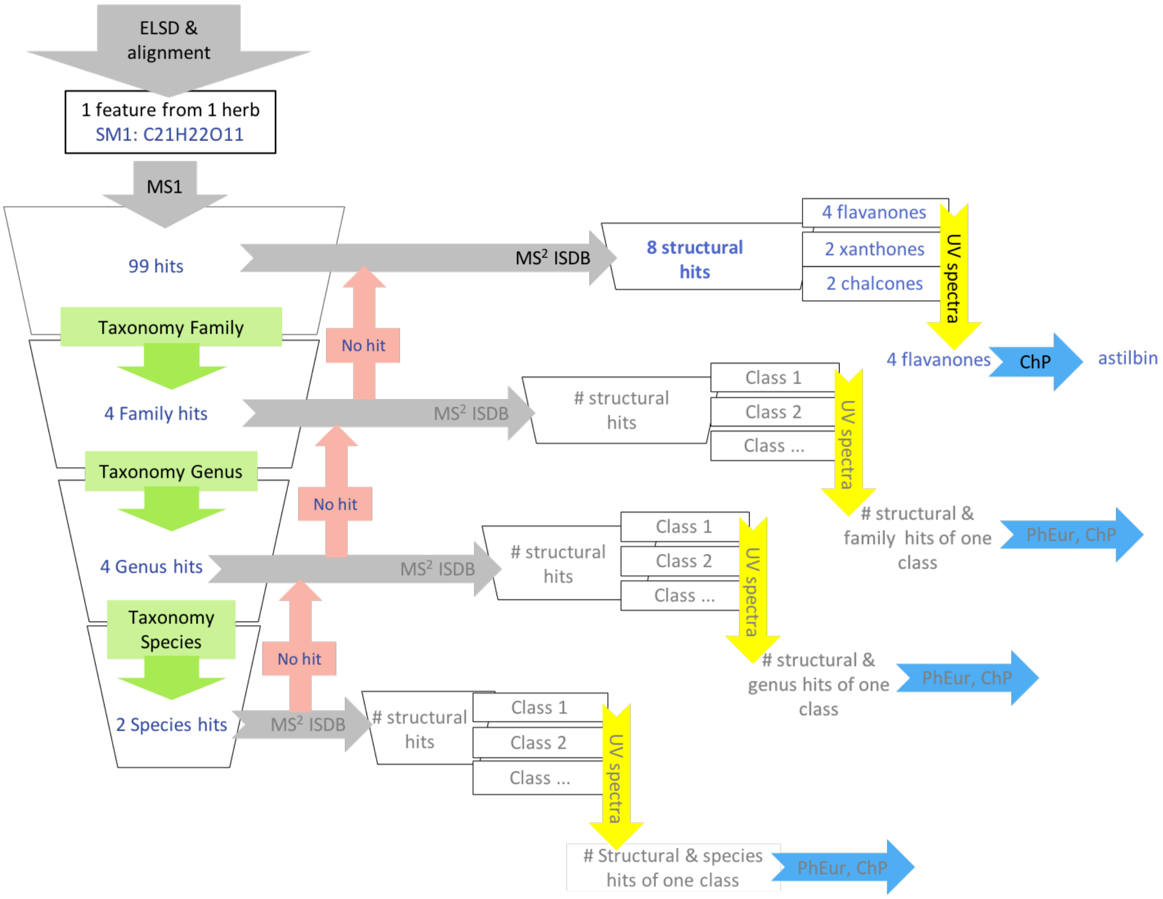

Supplementary Figure 14: annotation schema for SM1 (astilbin): no match between taxonomy and ISDB was found. The ISDB consultation retrieved 3 classes of compounds. UV spectrum discriminated them by demonstrating the presence of flavanone and discarding the chalcones and xanthones. If no concordant taxonomical hit was retrieved by the ISDB consultation, three pentahydroxyflavanones were suggested. The consultation of the Chinese Pharmacopoeia (CP) permitted to discriminate between them and to select the official marker, astilbin, which is not referenced in the DNP for *S.glabra*.

5.3 Summary of annotations

Supplementary Table S1: summary of the annotations

**HRMS/MS: 21 annotations**	**Number of cases**<br>**Cases**
**Taxonomy-ISDB consistency**	**17 (81%)**
1 structure	9
> 1 structure	8
> 1 class of components	4
UV discrimination	**4**
**Taxonomy-ISDB inconsistency**	4 (19%)
> 1 class of components	4
UV discrimination	**4**
**Number of structures of 1 class after UV discrimination**	
1 hit	11
2 to 4	10
Literature (ChP, PhEur) discrimination	2
Literature (ChP, PhEur) modification	1

+ 1 annotation at HRMS + taxonomic level => 22 annotations

<table>
<tr><th colspan="2">Formal identification</th><th colspan="2">NMR</th><th>Spiking</th></tr>
<tr><th colspan="2">Number of hits of 1 class after annotation</th><td>confirmation</td><td>modification</td><td>confirmation</td></tr>
<tr><td>1 hit</td><td>13</td><td>6</td><td>2</td><td>3</td></tr>
<tr><td>2 hits</td><td>2</td><td rowspan="3">3</td><td rowspan="3">2</td><td rowspan="3">-</td></tr>
<tr><td>3 hits</td><td>3</td></tr>
<tr><td>4 hits</td><td>4</td></tr>
<tr><td>Number of formally identified components</td><td>16</td><td></td><td></td><td></td></tr>
</table>

6 Identification and annotation tables

These tables summarize the identifications and annotations mentioned in the main text. The complete metadata is available in the Cytoscape files in doi:10.25345/C5516P] including IUPAC and InChI key.

Supplementary Table S2 : identification and annotations for *Angelica sinensis*

N°	Co-marker	RT (min)	Molecular formula	*m/z*	Feature	Spec[a]	Identification/ annotation	Tax[b]	MSI[c]	Chemical family	Cluster specificity PI[d]	NI[e]	CI[f]	ID[g]
**A1**		**10.37**	**$C_{12}H_{14}O_2$**	**191.1068**	**$[M+H]^+$**	**1**	**ligustilide**	**S**	**1**			**ND**		**110**
	Co-A1-1	10.11	$C_{12}H_{14}O_2$	191.1068	$[M+H]^+$	1	neoligustilide | buthylphtalide	G	3					252
	Co-A1-2	11.87	$C_{12}H_{14}O_2$	191.1068	$[M+H]^+$	1	neoligustilide | buthylphtalide	G	3					1270
	Co-A1-3	5.85	$C_{12}H_{14}O_4$	207.102	$[M+H]^+$	1	6, 7-epoxyligustilide | 9-hydroxyligustilide |	F	2	**isobenzofurans**	**85%**		**PI-17**	502
	Co-A1-4	6.20	$C_{12}H_{14}O_4$	207.102	$[M+H]^+$	1	6, 7-epoxyligustilide 9-hydroxyligustilide	F	2					698
	Co-A1-5	11.87	$C_{24}H_{28}O_4$	381.207	$[M+H]^+$	1	11 potential structures	S	3					721

[a] Spec: feature specificity; [b] Tax: taxonomic data referenced in (DNP, 2019a): S: species, G: genus, F: family, ND: not described, [c] MSI: *Metabolomic Standard Initiative*, level of identification proposed in (Sumner et al., 2007). [d] PI: positive ionization; [e] NI: negative ionization; [f] CI: cluster index; [g]: identification number in MZmine and Cytoscape.

Supplementary Table S3 : identification and annotations for *Chrysanthemum indicum*

N°	Co-marker	RT (min)	Molecular formula	*m/z*	Feature	Spec[a]	Identification/ annotation	Tax[b]	MSI[c]	Chemical family	Cluster specificity PI[d]	NI[e]	CI[f]	ID[g]
**C3**		**4.90**	$\mathbf{C_{25}H_{24}O_{12}}$	**517.1346**	$\mathbf{[M+H]^+}$	**1**	**1,3-Dicaffeoyl-epi-quinic acid**	**F**	**1**		**8%**			**148**
				**515.1196**	$\mathbf{[M\text{-}H]^-}$	**1**								**49**
C1	Co-C3-1	5.40	$C_{25}H_{24}O_{12}$	517.1344	$[M+H]^+$	1	trans-Di-O-caffeoylquinic acid	F	3					82
				515.1197	$[M\text{-}H]^-$	1								26
C8	Co-C3-2	4.84	$C_{25}H_{24}O_{12}$	517.1347	$[M+H]^+$		trans-Di-O-caffeoylquinic acid	F	3	**Caffeoylquinic acids**		**84%**	**P-I3 NI-34**	152
	Co-C3-3	4.61	$C_{25}H_{24}O_{12}$	515.1199	$[M\text{-}H]^-$	1	trans-Di-O-caffeoylquinic acid	F	3					396
	Co-C3-4	6.19	$C_{25}H_{24}O_{12}$	515.1198	$[M\text{-}H]^-$	1	trans-Di-O-caffeoylquinic acid							854
C6	Co-C3-5	1.37	$C_{16}H_{18}O_9$	355.1024	$[M+H]^+$	0.91	chlorogenic acid	F	2					184
				353.0882	$[M\text{-}H]^-$	0.89								102
**C2**		**5.20**	$\mathbf{C_{21}H_{20}O_{10}}$	**433.1131**	$\mathbf{[M+H]^+}$	**1**	**Cosmosiin**	**F**	**1**	**trihydoxyflavone**	**selfloop**			**58**
				**431.0985**	$\mathbf{[M\text{-}H]^-}$	**1**								**41**
**C4**		**6.08**	$\mathbf{C_{24}H_{22}O_{13}}$	**519.1131**	$\mathbf{[M+H]^+}$	**1**	**Cosmosiin-6''-O-Malonyl**	**F**	**1**	**trihydoxyflavone**	**selfloop**			**706**
				**517.0990**	$\mathbf{[M\text{-}H]^-}$	**1**								**195**
C5		4.31	$C_{21}H_{20}O_{11}$	449.1079	$[M+H]^+$	1	3',5,5',7-Tetrahydroxyflavone; 7-O-β-D-Glucopyranoside	NA	3	tetrahydroxyflavone	selfloop			206
				447.0937	$[M\text{-}H]^-$	0.94								117
C7		7.55	$C_{25}H_{24}O_{13}$	533.1294	$[M+H]^+$	0.99	QRM00[h] | LHF87[h] | LMY39[h]	NA		dihydroxymethoxyflavone malonylglucoside	selfloop			71
				531.1147	$[M\text{-}H]^-$	*ND-F*[i]								*1157*
C7-F			$C_{16}H_{12}O_5$	283.0613	[M-malonylhexose] (*m/z* -248.05321)	0.99								92

[a] Spec: feature specificity; [b] Tax: taxonomic data referenced in (DNP, 2019a): S: species, G: genus, F: family, ND: not described, [c] MSI: *Metabolomic Standard Initiative*, level of identification proposed in (Sumner et al., 2007). [d] PI: positive ionization; [e] NI: negative ionization; [f] CI: cluster index; [g]: identification number in MZmine and Cytoscape; [h] CRC number, structure available in (DNP, 2019b); [i] ND-F not detected in the formula.

Supplementary Table S4 : identification and annotations for *Glycyrrhiza uralensis*

N°	Co-marker	RT (min)	Molecular formula	*m/z*	Feature	Spec[a]	Identification/ annotation	Tax[b]	MSI[c]	Chemical family	Cluster specificity PI[d]	Cluster specificity NI[e]	CI[f]	ID[g]
**G1**		**8.57**	**$C_{42}H_{62}O_{16}$**	**823.4116**	**$[M+H]^+$**	**1**	**uralsaponin A**	**S**	**1**					**63**
				**821.3974**	**$[M-H]^-$**	**1**								**4**
G5	Co-G1-1	8.98	$C_{42}H_{62}O_{16}$	823.4116	$[M+H]^+$	1	licoricesaponin K2 or isomers	S/G	2					230
				821.3973	$[M-H]^-$	1	licoricesaponin K2 or isomers	S/G						38
	Co-G1-2	9.08	$C_{42}H_{62}O_{16}$	823.4116	$[M+H]^+$	1	licoricesaponin K2 or isomers	S/G						
	Co-G1-3	9.17	$C_{42}H_{62}O_{16}$	823.4116	$[M+H]^+$	1	licoricesaponin K2 or isomers	S/G						
	Co-G1-4	9.92	$C_{42}H_{64}O_{16}$	825.427	$[M+H]^+$	1	uralsaponin C | licoricesaponin J2	S					PI-1	
G6	Co-G1-6	8.24	$C_{42}H_{62}O_{17}$	839.4061	$[M+H]^+$	1	uralsaponin U | Licoricesaponin G2 or isomers	S/G	2	**oleanane triterpenoids**	**100%**	**90%**	**NI-8**	96
				837.3921	$[M-H]^-$	1								71
	Co-G1-5	8.24	$C_{42}H_{62}O_{17}$	839.4061	$[M+H]^+$	1	uralsaponin U | Licoricesaponin G2 or isomers	S/G						
	Co-G1-7	8.61	$C_{42}H_{62}O_{17}$	839.4061	$[M+H]^+$	1	uralsaponin U | Licoricesaponin G2 or isomers	S/G						
	Co-G1-8	7.81	$C_{42}H_{62}O_{17}$	839.4061	$[M+H]^+$	1	uralsaponin U | Licoricesaponin G2 or isomers	S/G						
G8	Co-G1-9	7.81	$C_{42}H_{62}O_{17}$	839.406	$[M+H]^+$	1	uralsaponin U | Licoricesaponin G2 or isomers	S/G	2					584
				837.3922	$[M-H]^-$	1								109
**G2**		**3.99**	**$C_{26}H_{30}O_{13}$**	**551.1762**	**$[M+H]^+$**	**1**	**liquiritin apioside**	**S**	**1**	**flavanones**				**168**
				**549.1617**	**$[M-H]^-$**	**1**								**17**
G3	Co-G2-2	3.84	**$C_{26}H_{30}O_{13}$**	551.176	$[M+H]^+$	1	glabroside	S	1	flavanones				1452
				549.1618	$[M-H]^-$	1								244
G9	Co-G2-1	5.81	$C_{26}H_{30}O_{13}$	551.1759	$[M+H]^+$	0.98	neolicuroside |isoliquiritin-apioside	S/G	2		**21%**	**38%**	**PI-3**	251
				549.1619	*$[M-H]^-$*	*ND-F*[i]				chalcones O-apiosides			**NI-5**	*1300*
	Co-G2-3	5.99	$C_{26}H_{30}O_{13}$	551.1762	$[M+H]^+$	1	neolicuroside |isoliquiritin-apioside	S/G	2					1258
	Co-G2-4	3.97	$C_{21}H_{22}O_9$	419.1330	$[M+H]^+$	1	liquiritin | neisoliquiritin | isoliquiritin	S/G	2					419
	Co-G2-5	6.00	$C_{21}H_{22}O_9$	419.1330	$[M+H]^+$	1	liquiritin | neisoliquiritin | isoliquiritin	S/G	2	flavanones				764
	Co-G2-6	6.19	$C_{21}H_{22}O_9$	419.1330	$[M+H]^+$	1	liquiritin | neisoliquiritin | isoliquiritin	S/G	2					1537
G4		1.46		165.0548	$[M-?]^-$	1	unknown							587
G7		6.15	$C_{22}H_{22}O_9$	431.1335	$[M+H]^+$	0.95	isoononin	G	2	isoflavones (2 substituants)	selfloop			106

[a] Spec: feature specificity; [b] Tax: taxonomic data referenced in (DNP, 2019a): S: species, G: genus, F: family, ND: not described, [c] MSI: *Metabolomic Standard Initiative*, level of identification proposed in (Sumner et al., 2007). [d] PI: positive ionization; [e] NI: negative ionization; [f] CI: cluster index; [g]: identification number in MZmine and Cytoscape; [h] CRC number, structure available in (DNP, 2019b); [i] ND-F not detected in the formula.

Supplementary Table S5 : identification and annotations for *Isatis tinctoria*

											Cluster specificity			
**N°**	**Co-marker**	**RT (min)**	**Molecular formula**	***m/z***	**Feature**	**Spec[a]**	**Identification/ annotation**	**Tax[b]**	**MSI[c]**	**Chemical family**	**PI[d]**	**NI[e]**	**CI[f]**	**ID[g]**
**I1**		**3.90**	**$C_{21}H_{20}O_{10}$**	**433.1131**	**$[M+H]^+$**	**0.99**	**isovitexin**	**S**	**1**	**flavones**				**232**
				**431.0986**	**$[M-H]^-$**	**0.99**								**147**
I2	Co-I1-1	4.33	$C_{22}H_{22}O_{11}$	463.1235	$[M+H]^+$	1	isoscoparin	S	2	Trihydroxymethoxyflavone -C-glucoside	**50%**	**80%**	**PI-57 NI-24**	195
				461.1095	$[M-H]^-$	1								172
	Co-I1-2	2.08	$C_{21}H_{20}O_{11}$	449.1080	$[M+H]^+$	1	HGS57[h] | LJX37[h] | KRF92[h]	NA	3	Tetrahydroxyflavone -C-glucoside				1530
				447.0939	$[M-H]^-$	1								719

[a] Spec: feature specificity; [b] Tax: taxonomic data referenced in (DNP, 2019a): S: species, G: genus, F: family, ND: not described, [c] MSI: *Metabolomic Standard Initiative*, level of identification proposed in (Sumner et al., 2007). [d] PI: positive ionization; [e] NI: negative ionization; [f] CI: cluster index; [g]: identification number in MZmine and Cytoscape; [h] CRC number, structure available in (DNP, 2019b).

Supplementary Table S6 : identification and annotations for *Oldenlandia diffusa*

N°	Co-marker	RT (min)	Molecular formula	*m/z*	Feature	Spec[a]	Identification/ annotation	Tax[b]	MSI[c]	Chemical family	Cluster specificity PI[d]	NI[e]	CI[f]	ID[g]
**O1**		**5.43**	**$C_{26}H_{30}O_{13}$**	**573.1580**	**$[M+Na]^+$**	**1**	**Scandoside; 10-O-(4-Hydroxy-Z-cinnamoyl), Me ester**		**1**	**Iridoid monoterpenoids**	**100%**	**100%**	**PI-102 NI-71**	**189**
				**549.1611**	**$[M-H]^-$**	**1**								**37**
O2		3.07	$C_9H_8O_3$	*165.0548*	*[M+H]+ (?)*	*ND-F[i]*	*coumaric acid (fragment?)*	*NA*			*selfloop*			*2256*
				163.0392	$[M-H]^-$ (?)	0.93								61
O3		1.16	$C_{18}H_{24}O_{12}$	455.1164	$[M+Na]^+$	1	asperulosidic acid	F	2	Iridoid monoterpenoids	100%		PI-186	193
		1.18		431.1197	$[M-H]^-$	1								45
O4				*ND*	*?*	*ND-F[i]*								
		3.72		163.0391	$[M-H]^-$ (?)	1	coumaric acid (fragment?)	NA			selfloop			774
O5	Co-O-1	4.87	$C_{37}H_{38}O_{20}$	803.203	$[M+H]^+$	1	KBY51 [h]	S	2	flavonol-di-glycoside-cinnamoyl	60%	71%	PI-100 NI-28	272
				801.1896	$[M-H]^-$	1		S	2					116
	Co-O-2	4.63	$C_{38}H_{40}O_{21}$	833.213	$[M+H]^+$	1	OYT10 [h]	S	2					508
	Co-O-3	5.38	$C_{37}H_{38}O_{19}$	787.208	$[M+H]^+$	1	JOH23 [h]							1027
O6		1.73	$C_{18}H_{22}O_{11}$	432.1503	$[M+NH_4]^+$ (*m/z* +17.0265)	1	6'-Acetyl-deacetylasperuloside.	G	2	Iridoid monoterpenoids	25%		PI-3	224
				415.1235	$[M+H]^+$	1								784
				**459.115**	$[M+FA]^-$	1								31
O7		0.98	$C_{17}H_{24}O_{11}$	427.1211	$[M+Na]^+$	1	feretoside	G	2	Iridoid monoterpenoids	selfloop			128
				449.1306	$[M+FA]^-$	1								39
SC1 (O8)		6.16	$C_{21}H_{18}O_{11}$	447.0918	$[M+H]^+$	0.06	baicalin	S	1	flavones				3
				445.0780	$[M-H]^-$	0.17								10

[a] Spec: feature specificity; [b] Tax: taxonomic data referenced in (DNP, 2019a): S: species, G: genus, F: family, ND: not described, [c] MSI: *Metabolomic Standard Initiative*, level of identification proposed in (Sumner et al., 2007). [d] PI: positive ionization; [e] NI: negative ionization; [f] CI: cluster index; [g]: identification number in MZmine and Cytoscape; [h] CRC number, structure available in (DNP, 2019b); [i] ND-F not detected in the formula.

Supplementary Table S7 : identification and annotations for *Polygonum cuspidatum*

											Cluster specificity			
N°	**Co-marker**	**RT (min)**	**Molecular formula**	***m/z***	**Feature**	**Spec**[a]	**Identification/ annotation**	**Tax**[b]	**MSI**[c]	**Chemical family**	**PI**[d]	**NI**[e]	**CI**[f]	**ID**[g]
PO1		**3.76**	**$C_{20}H_{22}O_8$**	**391.1395**	**$[M+H]^+$**	**1**	**E-piceid**		**1**					**145**
				**389.1244**	**$[M-H]^-$**	**1**								**52**
				435.1300	$[M+FA]^-$	**0.99**								
	Co-Po-1	2.55	$C_{20}H_{22}O_8$	391.1395	$[M+H]^+$	1	resveratroloside	NA	3	**stilbenes**	**11%**	**6%**	**PI-3 NI-5**	1636
	Co-Po-2	5.13	$C_{14}H_{12}O_3$	229.086	$[M+H]^+$	1	resveratrol analog	NA	3					1619
	Co-Po-3	5.50		229.086	$[M+H]^+$	1	resveratrol analog							656
	Co-Po-4	5.79		229.0862	$[M+H]^+$	1	resveratrol analog							1067
PO2		**7.07**	**$C_{21}H_{20}O_{10}$**	**455.0950**	**$[M+Na]^+$**	**1**	**emodin-8-glucoside**	**1**	**1**	**anthraquinones**	**selfloop**			416
			**$C_{15}H_{10}O_5$**	**271.0599**	**$[M\text{-hexose}]^+$** (*m/z* -162.0528)	**0.99**								**135**
			**$C_{21}H_{20}O_{10}$**	**431.0986**	**$[M-H]^-$**	**0.98**								**13**
PO3		*2.69*	*$C_{37}H_{30}O_{16}$*	*731.1606*	*[M+H]+*	*ND-F*[i]	*3'-galloylprocyanidin B1 or B2*	*G*		*proanthocyanidin flavonoids*				*2336*
				729.1472	*$[M-H]^-$*	*ND-F*[i]								*1833*
PO4		2.03	$C_{15}H_{14}O_6$	291.0862	$[M+H]^+$	0.87	Catechin | epicatechin	G			60%		PI 120	752
			$C_{37}H_{30}O_{16}$	*731.1607*	*$[M+H]^+$*	*ND-F*[i]	*3'-galloylprocyanidin B1 or B2*	*G*		*proanthocyanidin flavonoids*				*2388*
PO4-F			*$C_{15}H_{14}O_6$*	*289.0721*	*$[M-H]^-$*	*0.88*								*171*
PO4			*$C_{37}H_{30}O_{16}$*	*729.1472*	*$[M-H]^-$*	*ND-F*[i]								*1778*
PO5		*3.66*	*$C_{44}H_{30}O_{20}$*	*883.1716*	*$[M+H]^+$*	*ND-F*[i]	*3,3'-digalloylprocyanidin B2*			*proanthocyanidin flavonoids*				*2372*
		3.73		*881.1584*	*$[M-H]^-$*	*ND-F*[i]		*G*						*1777*

[a] Spec: feature specificity; [b] Tax: taxonomic data referenced in (DNP, 2019a): S: species, G: genus, F: family, ND: not described, [c] MSI: *Metabolomic Standard Initiative*, level of identification proposed in (Sumner et al., 2007). [d] PI: positive ionization; [e] NI: negative ionization; [f] CI: cluster index; [g]: identification number in MZmine and Cytoscape; [i] ND-F not detected in the formula.

Supplementary Table S8 : identification and annotations for *Prunella vulgaris*

											Cluster specificity			
**N°**	**Co-marker**	**RT (min)**	**Molecular formula**	***m/z***	**Feature**	**Spec[a]**	**Identification/ annotation**	**Tax[b]**	**MSI[c]**	**Chemical family**	**PI[d]**	**NI[e]**	**CI[f]**	**ID[g]**
**PR1**		**5.49**	$\mathbf{C_{18}H_{16}O_8}$	**361.0918**	$\mathbf{[M+H]^+}$	**1**	**rosmarinic acid**	**S**	**1**					646
				**359.0778**	$\mathbf{[M-H]^-}$	1								22
			$C_9H_6O_3$	163.039	$[M-?]^+$	1								340
PR2	Co-PR1-1	4.63	$C_{24}H_{26}O_{13}$	521.1306	$[M-H]^-$	1	salviaflaside	F	2	**rosmarinic/salvianolic acid lignans**	**6%**	**69%**	**PI-3 NI-10**	105
			$C_9H_6O_3$	163.0388	$[M-?]^+$	1								1159
PR3	Co-PR1-2	2.27	$C_{18}H_{18}O_9$	377.0883	$[M-H]^-$	0.93	danshensuan C	F	2					947
		1.94	$C_9H_8O_4$	*181.0496*		*ND-F[i]*								*2405*
				179.0341		0.85								136

[a] Spec: feature specificity; [b] Tax: taxonomic data referenced in (DNP, 2019a): S: species, G: genus, F: family, ND: not described, [c] MSI: *Metabolomic Standard Initiative*, level of identification proposed in (Sumner et al., 2007). [d] PI: positive ionization; [e] NI: negative ionization; [f] CI: cluster index; [g]: identification number in MZmine and Cytoscape; [i] ND-F not detected in the formula.

Supplementary Table S9 : identification and annotations for *Scutellaria baicalensis*

N°	Co-marker	RT (min)	Molecular formula	*m/z*	Feature	Spec[a]	Identification/ annotation	Tax[b]	MSI[c]	Chemical family	Cluster specificity PI[d]	NI[e]	CI[f]	ID[g]
**SC1**		**6.16**	**$C_{21}H_{18}O_{11}$**	**447.0918**	**$[M+H]^+$**	**0.94**	**baicalin**	**S**	**1**					**3**
				**445.0780**	**$[M-H]^-$**	**0.93**								**10**
SC4	Co-Sc1-1	6.63	$C_{21}H_{18}O_{11}$	447.0914	$[M+H]^+$	1	2', 5, 7-trihydroxyflavone-7-O-glucoside | 5, 7, 8-trihydroxyflavone-7-O-glucoside	G	2					18
				445.0781	$[M-H]^-$	1				**Flavones (3 O substituents)**	**14%**	**25%**	**PI-3 NI-5**	23
	Co-Sc1-2	7.11	$C_{21}H_{18}O_{11}$	447.0923	$[M+H]^+$	0.99	2', 5, 7-trihydroxyflavone-7-O-glucoside | 5, 7, 8-trihydroxyflavone-7-O-glucoside	G	2					105
				445.0781	$[M-H]^-$	1								
	Co-Sc1-3	7.02	$C_{22}H_{20}O_{12}$	477.1030	$[M+H]^+$	0.98	hispidulin-7-glucuronide | NMM41 [h]	S/G	3					31
**SC2**		**7.16**	**$C_{22}H_{20}O_{11}$**	**461.1079**	**$[M+H]^+$**	**0.91**	**wogonoside**	**S**	**1**		**selfloop**			**6**
				**459.0938**	**$[M-H]^-$**	**0.85**				**flavones (3 O substituents); O-glucuronides**				**5**
**SC3**		**6.89**	**$C_{22}H_{20}O_{11}$**	**461.1078**	**$[M+H]^+$**	**0.97**	**oroxyloside**	**S**	**1**		**selfloop**			**7**
				**459.0938**	**$[M-H]^-$**	**0.93**								**8**
SC8	Co-Sc2-1	6.81	$C_{22}H_{20}O_{12}$	475.0886	$[M-H]^-$	1	hispidulin-7-glucuronide | NMM41 [h]	S/G	3	trihydroxymethoxyflavone		**96%**	**NI-1**	74
	Co-Sc2-2	7.04	$C_{22}H_{20}O_{12}$	475.0886	$[M-H]^-$	1	hispidulin-7-glucuronide | NMM41 [h]	S/G	3					50
	Co-Sc2-3	7.29	$C_{23}H_{22}O_{12}$	489.1041	$[M-H]^-$	1	LHK67 [h]	G	2	tetrahydroxymethoxyflavone				79
	Co-Sc2-4	6.25	$C_{23}H_{22}O_{13}$	505.0992	$[M-H]^-$	1	MWZ53 [h] or isomers	G/NA	3	pentahydroxyflavone				519
	Co-Sc2-5	6.14	$C_{23}H_{22}O_{13}$	505.0992	$[M-H]^-$	1								357
	Co-Sc2-6	7.97	$C_{17}H_{14}O_{7}$	329.0668	$[M-H]^-$	1	thymusin	F	2	trihydroxydimethoxyflavone				209
	Co-Sc2-7	9.29	$C_{17}H_{14}O_{6}$	313.0721	$[M-H]^-$	1	velutin	F		dihydroxydimethoxyflavone				216
SC5		7.99	$C_{15}H_{10}O_{5}$	271.0602	$[M+H]^+$	1	baicalein | norwogonin |	S|2G	3	flavones (3 O substituents)	selfloop		selfloop	143

						2',5,7-trihydroxflavone						
			269.0458	[M-H]-	1							139
SC6	4.10	$C_{26}H_{28}O_{13}$	549.1603	$[M+H]^+$	0.96	chrysin 6-C-glucoside 8-C-arabinoside| chrysin 6-C-arabinoside 8-C-glucoside	S	2	flavones (3 O substituents) di-C-glycosides	61%	PI-74	35
			547.1463	$[M-H]^-$	0.98							40
SC7	6.52	$C_{21}H_{20}O_{11}$	*449.1075*	*$[M+H]^+$*	*ND-F*[i]	*dihydrobaicalin or CYL29*[h]	*S*	*2*	*flavones (4 O substituents); O-glucuronides*		*PI-3*<br>*NI-5*	*2551*
			447.0924	*$[M-H]^-$*	*ND-F*[i]							*1888*
SC9	4.65	$C_{26}H_{28}O_{13}$	549.1604	$[M+H]^+$	0.97	chrysin 6-C-glucoside 8-C-arabinoside | chrysin 6-C-arabinoside 8-C-glucoside	S	2	flavones (3 O substituents) di-C-glycosides	61%	PI-74	42
			547.1464	$[M-H]^-$	0.97							33
SC10	4.98	$C_{27}H_{30}O_{14}$	579.1711	$[M+H]^+$	1	nivyaside | RDD41[h] | KRG85[h]	NA	3	flavones (3 O substituents) di-C-glycosides	50%	PI-57	827
SC11	5.19	$C_{21}H_{20}O_9$	417.118	$[M+H]^+$	1	chrysin 6-C-glucopyranoside | chrysin 8-C-glucopyranoside.	S	2	flavones (3 O substituents) C-glycosides	50%	PI-57	588
SC12	6.22	$C_{21}H_{20}O_9$	417.117	$[M+H]^+$	1	chrysin 6-C-glucopyranoside | chrysin 8-C-glucopyranoside.	S	2	flavones (3 O substituents) C-glycosides	50%	PI-57	455

[a] Spec: feature specificity; [b] Tax: taxonomic data referenced in (DNP, 2019a): S: species, G: genus, F: family, ND: not described, [c] MSI: *Metabolomic Standard Initiative*, level of identification proposed in (Sumner et al., 2007). [d] PI: positive ionization; [e] NI: negative ionization; [f] CI: cluster index; [g]: identification number in MZmine and Cytoscape; [h] CRC number, structure available in (DNP, 2019b); [i] ND-F not detected in the formula.

Supplementary Table S10 : identification and annotations for *Smilax glabra*

N°	Co-marker	RT (min)	Molecular formula	*m/z*	Feature	Spec[a]	Identification/ annotation	Tax[b]	MSI[c]	Chemical family	Cluster specificity PI[d]	NI[e]	CI[f]	ID[g]
**SM1**		**4.33**	**$C_{21}H_{22}O_{11}$**	**451.1236**	**$[M+H]^+$**	**1**	**astilbin**	**S**	**1**	**pentahydroxyflavanone-O-rhamoside**	**100%**	**99%**	**PI-25 NI22**	**98**
				**449.1088**	**$[M-H]^-$**	**1**								**19**
	Co-SM1-1	4.92	$C_{21}H_{22}O_{11}$	451.1237	$[M+H]^+$	1	OBC08 | aceronidin | NJL24[h]	NA	3					257
	Co-SM1-2	4.04	$C_{21}H_{22}O_{11}$	451.1237	$[M+H]^+$	1	OBC08 | aceronidin | NJL24 [h]	NA	3					315
	Co-SM1-3	5.02	$C_{21}H_{22}O_{11}$	451.1237	$[M+H]^+$	1	OBC08 | aceronidin | NJL24 [h]	NA	3					667
	Co-SM1-4	4.98	$C_{21}H_{20}O_{11}$	449.108	$[M+H]^+$	1	PYZ08 [h] | POT37 [h] | HBN30 [h] | KQW57| [h] NRN98 [h] | LHZ69 [h]	NA	3					716
	Co-SM1-5	2.90	$C_{15}H_{16}O_9$	341.087	$[M+H]^+$	1	eucryphin	S	2	benzopyran				779

[a] Spec: feature specificity; [b] Tax: taxonomic data referenced in (DNP, 2019a): S: species, G: genus, F: family, ND: not described, [c] MSI: *Metabolomic Standard Initiative*, level of identification proposed in (Sumner et al., 2007). [d] PI: positive ionization; [e] NI: negative ionization; [f] CI: cluster index; [g]: identification number in MZmine and Cytoscape; [h] CRC number, structure available in (DNP, 2019b).

Supplementary Table S11 : identification and annotations for *Sophora flavescens*

N°	Co-marker	RT (min)	Molecular formula	*m/z*	Feature	Spec[a]	Identification/ annotation	Tax[b]	MSI[c]	Chemical family	Cluster specificity PI[d]	NI[e]	CI[f]	ID[g]
**SO1-1**		**0.83**	**$C_{15}H_{24}N_2O_2$**	**265.1911**	**$[M+H]^+$**	**0.98**	**oxymatrine**	**S**	**1**			**NA**		**1**
	Co-SO1-1	0.83	$C_{15}H_{22}N_2O_2$	263.1756	$[M+H]^+$	0.97	allomatrine isomers	G	3					5
	Co-SO1-2	0.63	$C_{15}H_{20}N_2O$	245.1644	$[M+H]^+$	1	isosophoramine	G	2					202
	Co-SO1-3	0.77	$C_{15}H_{22}N_2O$	247.1809	$[M+H]^+$	1	5-episophocarpine	S	2	**quinolizidine alkaloid**	**89%**		**PI-4**	21
	Co-SO1-4	1.04	$C_{15}H_{20}N_2O_2$	261.1601	$[M+H]^+$	1	7-hydroxysophoramine	G	2			-		798
	Co-SO1-6	1.1	$C_{15}H_{22}N_2O_2$	263.1756	$[M+H]^+$	1	allomatrine isomers	G	3					29
	Co-SO1-7	1.59	$C_{15}H_{24}N_2O_3$	281.1859	$[M+H]^+$	1	6,7-dihydroxylupanine	G	2					81
	Co-SO-1	7.67	$C_{26}H_{32}O_7$	457.222	$[M+H]^+$	1	kushenol F	S	2					
	Co-SO-2	7.73	$C_{26}H_{32}O_7$	457.222	$[M+H]^+$	1	kurarinol	S	2					
	Co-SO-3	7.95	$C_{26}H_{32}O_7$	457.2222	$[M+H]^+$	1	PPB59[h]	S	2					
	Co-SO-3	7.62	$C_{26}H_{30}O_7$	455.2057	$[M+H]^+$	1	kushenol I	S/G	3	prenylated flavanone	97%	15%	PI-26	
	Co-SO-4	9.18	$C_{26}H_{30}O_7$	455.2064	$[M+H]^+$	1	leachianone D	S/G	3				NI-10	
				453.1927	$[M-H]^-$	1								259
	Co-SO-5	9.48	$C_{26}H_{30}O_6$	437.1976	$[M-H]^-$	1	cyclokaridin	S	2					46
	Co-SO-6	10.18	$C_{25}H_{28}O_6$	423.182	$[M-H]^-$	1	kushenol F	S	2					253

[a] Spec: feature specificity; [b] Tax: taxonomic data referenced in (DNP, 2019a): S: species, G: genus, F: family, ND: not described, [c] MSI: *Metabolomic Standard Initiative*, level of identification proposed in (Sumner et al., 2007). [d] PI: positive ionization; [e] NI: negative ionization; [f] CI: cluster index; [g]: identification number in MZmine and Cytoscape; [h] CRC number, structure available in (DNP, 2019b).

7 Description of isolated components

1,3-Dicaffeoyl-epi-quinic acid (C1). Pure in M6S8 and S9. UV max: 219; 328 nm. ^{1}H NMR (DMSO-d_6, 600 MHz) δ 1.84 (1H, t, *J*=11.7 Hz, H-2"), 2.22 (1H, d, *J*=14.2 Hz, H-6"), 2.32 (1H, d, *J*=12.9 Hz, 2'), 2.36 (1H, m, H-6'), 3.55 (1H, d, *J*=8.9 Hz, H-4), 4.04 (1H, s, H-5), 5.22 (1H, td, *J*=9.1, 4.2 Hz, H-3), 6.17 (1H, d, *J*=15.9 Hz, H-2"), 6.23 (1H, d, *J*=15.9 Hz, H-2'), 6.75 (1H, d, *J*=8.1 Hz, H-8"), 6.75 (1H, d, *J*=8.1 Hz, H-8'), 6.95 (1H, dd, *J*=8.1, 2.1 Hz, H-9"), 6.98 (1H, dd, *J*=8.1, 2.1 Hz, H-9'), 7.03 (1H, d, *J*=2.1 Hz, H-5"), 7.08 (1H, d, *J*=2.1 Hz, H-5'), 7.40 (1H, d, *J*=15.9 Hz, H-3"), 7.47 (1H, d, *J*=15.9 Hz, H-3'); ^{13}C NMR (DMSO-d_6, 151 MHz) δ 34.6 (C-6), 36.8 (C-2), 68.2 (C-5), 70.3 (C-3), 71.6 (C-4), 114.4 (C-2'), 114.8 (C-5"), 114.9 (C-5'), 115.5 (C-2"), 115.8 (C-8', C-8"), 120.7 (C-9"), 121.3 (C-9'), 125.5 (C-4'), 125.7 (C-4"), 144.1 C- (3"), 144.9 (C-3'), 145.7 (C-6', C-6"), 148.2 (C-7'), 148.6 (C-7"), 165.0 (C-1"), 166.1 (C-1'). ESI-HRMS: *m/z* 515.1191 (calculated for $C_{25}H_{23}O_{12}$, *m/z* 515.1190, 1.2 ppm) (Patiny and Borel, 2013) (Kim and Lee, 2005).

Cosmosiin (C2). Impur in M21. UV max: 266, 340 nm.^{1}H NMR (MeOD, 600 MHz) δ 3.41 (1H, t, *J*=9.6, 8.9 Hz, H-4"), 3.50 (2H, m, H-2", H-3"), 3.55 (1H, m, H-5"), 3.73 (1H, dd, *J*=12.1, 5.9 Hz, H-6"b), 3.95 (1H, dd, *J*=12.2, 2.3 Hz, H-6"a), 5.07 (1H, d, *J*=7.0 Hz, H-1"), 6.49 (1H, d, *J*=2.1 Hz, H-6), 6.65 (1H, s, H-3), 6.81 (1H, d, *J*=2.1 Hz, H-8), 6.93 (2H, d, *J*=8.4 Hz, H-3', H-5'), 7.88 (2H, d, *J*=8.4 Hz, H-2', H-6'); ^{13}C NMR (MeOD, 151 MHz) δ 62.5 (C-6"), 71.3 (C-4"), 74.7 (C-2"), 77.9 (C-3"), 78.4 (C-5"), 96.1 (C-8), 101.2 (C-6), 101.6 (C-1"), 104.1 (C-3), 107.1 (C-10), 117.0 (C-3', C-5'), 123.1 (C-1'), 129.6 (C-2', C-6'), 159.0 (C-9), 162.9 (C-5), 162.9 (4'), 164.8 (C-7), 166.8 (C-2), 184.1 (C-4). ESI-HRMS: m/z 431.0988 (calculated for $C_{21}H_{19}O_{10}$, *m/z* 431.0978, 1.0 ppm) (Redaelli et al., 1980).

Malonylcosmosiin (C4) (Cosmosiin; 6''-*O*-malonyl). Pure in M23S7**.** UV max: 266; 336 nm. ^{1}H NMR (DMSO-d_6, 600 MHz) δ 3.11 (2H, m, H-8"), 3.21 (1H, t, *J*=9.3 Hz, H-4"), 3.28 (1H, overlapped, H-2"), 3.31 (1H, overlapped, H-3"),3.72 (1H, ddd, *J*=9.3, 6.9, 2.0 Hz, H-5"), 4.12 (1H, dd, *J*=12.1, 6.9 Hz, H-6"b), 4.30 (1H, dd, *J*=12.1, 2.0 Hz, H-6"a), 5.06 (1H, d, *J*=7.4 Hz, H-1"), 6.46 (1H, d, *J*=2.3 Hz, H-6), 6.78 (1H, d, *J*=2.3 Hz, H-8), 6.83 (1H, s, H-3), 6.94 (2H, d, *J*=8.6 Hz, H-3', H-5'), 7.95 (2H, d, *J*=8.6 Hz, H-2', H-6'); ^{13}C NMR (DMSO-d_6, 151 MHz) δ 44.2 (C-8"), 63.5 (C-6"), 69.6 (C-4"), 73.1 (C-1"), 74.1 (C-5"), 76.0 (C-3"), 94.7 (C-8), 99.6 (C-6), 99.9, 103.2 (C-3), 105.7 (C-10), 116.1 (C-3', C-5'), 121.0 (C-1'), 128.6 (C-2', C-6'), 157.1 (C-9), 161.2 (C-5), 161.4 (4'), 162.7 (C-7), 164.0 (C-2), 167.7 (C-9"), 168.5 (C-7"), 181.6 (C-4). ESI-HRMS: *m/z* 517.0995 (calculated for $C_{24}H_{21}O_{13}$, *m/z* 517.0982, 1.5 ppm) (Svehlikova et al., 2004).

Uralsaponin A (G1) (Glycyrrhetic acid; 1'-Epimer). Amorphous powder. Major in M34. UV max 250 nm. ^{1}H NMR (DMSO-d_6, 500 MHz) δ 0.71 (1H, m, H-5), 0.71 (3H, s, H-24), 0.75 (3H, s, H-28), 0.95 (3H, s, H-23), 0.96 (2H, m, H-1b, H-16b), 1.03 (6H, s, H-25, H-26), 1.09 (3H, s, H-29), 1.14 (1H, m, H-15b), 1.26 (1H, m, H-22b), 1.34 (4H, m, H-6b, H-7b, H-21b, H-22a), 1.34 (3H, s , H-27), 1.49 (1H, m, H-6a), 1.59 (1H, m, H-2b),

1.62 (1H, m, H-7a), 1.65 (1H, m, H-2a), 1.68 (2H, m, H-19), 1.73 (1H, m, H-15a), 1.80 (1H, m, H-21a), 2.05 (1H, m, H-18), 2.08 (1H, m, H-16a), 2.32 (1H, m, H-9), 2.55 (1H, dt, *J*=13.5, 3.4 Hz, H-1a), 3.04 (2H, m, H-2'', H-3), 3.19 (1H, m, H-3''), 3.26 (1H, t, *J*=6.5 Hz, H-4''), 3.31 (1H, m, H-2'), 3.36 (1H, t, *J*=9.2 Hz, H-4'), 3.41 (1H, t, *J*=9.2 Hz, H-3'), 3.55 (1H, d, *J*=9.7 Hz, H-5''), 3.63 (1H, d, *J*=9.4 Hz, H-5'), 4.40 (1H, d, *J*=7.5 Hz, H-1'), 4.49 (1H, d, *J*=7.7 Hz, H-1''), 5.39 (1H, s, H-12); ^{13}C NMR (DMSO-d_6, 126 MHz) δ 15.5 (C-24), 15.8 (C-25), 16.5 (C-6), 18.0 (C-26), 22.6 (C-27), 25.3 (C-2), 25.4 (C-16), 25.6 (C-15), 26.8 (C-23), 27.4 (C-29), 28.0 (C-28), 30.0 (C-21), 31.1 (C-17), 31.7 (C-7), 35.9 (C-10), 37.2 (C-22), 38.2 (C-1), 38.6 (C-4), 40.3 (C-19), 42.5 (C-14), 42.7 (C-20), 44.5 (C-8), 47.7 (C-18), 53.9 (C-5), 60.7 (C-9), 70.9 (C-4'), 71.1 (C-4''), 74.5 (C-2''), 74.7 (C-5'), 75.3 (C-3''), 75.5 (C-3'), 75.8 (C-5''), 82.2 (C-2'), 87.8 (C-3), 103.0 (C-1'), 104.3 (C-1''), 126.9 (C-12), 169.2 (C-13), 169.6 (C-6''), 169.9 (C-6'), 177.2 (C-30), 198.6 (C-11). ESI-HRMS : *m/z* 821.3981 [M-H]$^-$, calculated for $C_{42}H_{61}O_{16}$, *m/z* 821.3960, 2.0 ppm) (Baltina et al., 2005).

Liquiritin apioside (G2) (4'-7-dihydroxyflavanone ; 4'-*O*-[β-D-apiofuranosyl-(1→2)-β-D-glucopyranoside]). Amorphous powder. Pure in M15S2.UV max: 220; 275; 325 nm. ^{1}H NMR (DMSO-d_6, 500 MHz) δ 2.67 (1H, dd, *J*=16.7, 2.9 Hz, H-3b), 3.14 (2H, m, H-3', H-4'', 3', 4''), 3.31 (2H, m, H-5'', H-5'''a), 3.47 (3H, m, H-2'', H-3'', H-6''b), 3.64 (1H, d, *J*=9.4 Hz, H-4'''b), 3.70 (1H, dt, *J*=12.1, 2.5 Hz, H-6''a), 3.75 (1H, m, H-2'''), 3.95 (1H, d, *J*=9.4 Hz, H-4'''a), 4.95 (1H, d, *J*=7.5 Hz, H-1''), 5.36 (1H, d, *J*=1.2 Hz, H-1'''), 5.52 (1H, dt, *J*=12.9, 3.3 Hz, H-2), 6.35 (1H, d, *J*=2.2 Hz, H-8), 6.51 (1H, dd, *J*=8.7, 2.2 Hz, H-6), 7.06 (2H, d, *J*=8.6 Hz, H-3', H-5'), 7.44 (4H, d, *J*=8.6 Hz, H-2', H-6'), 7.65 (1H, d, *J*=8.7 Hz, H-5); ^{13}C NMR (DMSO-d_6, 126 MHz) δ 43.0 (C-3), 60.5 (C-6''), 64.2 (C-5'''), 69.7 (C-4''), 73.8 (C-4'''), 75.6 (C-2''), 76.0 (C-2'''), 76.8 (C-5''), 76.9 (C-3''), 78.5 (C-2), 79.2 (C-3'''), 98.5 (C-1''), 102.4 (C-8), 108.6 (C-1'''), 110.5 (C-6), 113.3 (C-10), 115.9 (C-3', C-'5'), 127.8 (C-2', C-6'), 128.2 (C-5), 132.2 (C-1'), 157.1 (C-4'), 162.9 (C-7), 164.5 (C-9), 189.7 (C-4). ESI-HRMS : *m/z* 549.1620 [M-H]$^-$, calculated for $C_{26}H_{29}O_{13}$, *m/z* 549.50167, 1.2 ppm) (Kitagawa et al., 1994).

10-*O-p-cis*-Coumaroyl Scandoside Methyl Ester (O1). Major in M20S2. UV max 227; 312 nm. ^{1}H NMR (DMSO-d_6, 500 MHz) δ 2.99 (2H, m, H-2', H-9), 3.05 (1H, t, *J*=9.4 Hz, H-4'), 3.16 (3H, m, H-3', H-5', H-5), 3.44 (1H, dd, *J*=11.9, 6.2 Hz, H-6'b), 3.58 (4H, s, H-12), 3.67 (1H, d, *J*=11.9 Hz, H- 6'a), 4.04 (1H, d, *J*=15.9 Hz, H-10b), 4.19 (1H, d, *J*=15.9 Hz, 10a), 4.49 (1H, d, *J*=8.0 Hz, H-1'), 5.31 (1H, d, J=5.9 Hz, H-1), 5.53 (1H, bs, H-6), 5.74 (1H, s, H-7), 5.76 (1H, d, *J*=12.9 Hz, H-2''), 6.76 (2H, d, *J*=8.4 Hz, H-6'', H-8''), 6.86 (1H, d, *J*=12.9 Hz, H-3''), 7.47 (1H, s, H-3), 7.66 (2H, d, *J*=8.4 Hz, H-5'', H-9''); ^{13}C NMR (DMSO-d_6, 126 MHz) δ 39.8 (C-5), 45.6 (C-9), 51.0 (C-12), 58.9 (C-10), 61.0 (C-6'), 70.0 (C-4'), 73.2 (C-2'), 76.6 (C-3'), 77.3 (C-5'), 81.4 (C-6), 94.9 (C-1), 98.4 (C-1'), 108.1 (C-4), 114.9 (C-6'', C-8''), 115.4 (C-2''), 124.5 (C-7), 125.3 (C-4''), 132.6 (C-5'', C-9''), 143.1 (C-3''), 150.1 (C-8), 152.3 (C-3), 158.9 (C-7''), 165.5 (C-1''), 166.5 (C-11). ESI-HRMS: m/z 549.1623 (calculated for $C_{26}H_{29}O_{13}$, m/z 549.16082, 1.7 ppm) (Otsuka et al., 1991).

E-Piceid (PO1) 1-(3,5-Dihydrophenyl)-2-(4-hydroxyphenyl)-ethylene ; (E)-form, 3-*O*-β-D-glucopyranoside). Amorphous powder. Pure in M14S12. UV max: 221; 281; 425 nm. ^{1}H NMR (DMSO-d_6, 600 MHz) δ 3.16 (1H, t, *J*=9.0 Hz, H-4'), 3.21 (1H, t, *J*=9.0, 7.7 Hz, H-2'), 3.27 (1H, t, *J*=9.0 Hz, H-3'), 3.31 (1H, overlapped, H-5'), 3.48 (1H, dt, *J*=11.0, 5.4 Hz, H-6'b), 3.72 (1H, dd, *J*=11.0, 5.4 Hz, H-6'a), 4.61 (1H, t, *J*=5.4 Hz, 6'OH), 4.79 (1H, d, *J*=7.7 Hz, H-1'), 5.01 (1H, s, 4'OH), 5.07 (1H, s, 3'OH), 5.26 (1H, s, 2'OH), 6.33 (1H, t, *J*=1.8 Hz, H-4), 6.56 (1H, t, *J*=1.8 Hz, H-6), 6.72 (1H, t, *J*=1.8 Hz, H-2), 6.75 (2H, d, *J*=8.6 Hz, H-11, H-13), 6.86 (1H, d, *J*=16.3 Hz, H-7), 7.02 (1H, d, *J*=16.3 Hz, H-8), 7.39 (2H, d, *J*=8.6 Hz, H-10, H-14), 9.43 (1H, s, 5OH), 9.57 (1H, s, 12OH); ^{13}C NMR (DMSO-d_6, 151 MHz) δ 60.7 (C-6'), 69.8 (C-4'), 73.3 (C-2'), 76.7 (C-3'), 77.1 (C-5'), 100.7 (C-1'), 102.7 (C-4), 104.7 (C-2), 107.2 (C-6), 115.5 (C-11, C-13), 125.2 (C-7), 127.9 (C-10, C-14), 128.0 (C-9), 128.5 (C-8), 139.3 (C-1), 157.3 (C-12), 158.4 (C-5), 158.9 (C-3). ESI-HRMS m/z 389.1246 [M-H]$^-$ (calculated for $C_{20}H_{19}O_8$ m/z 389.1236, 1.1ppm) (Chen et al., 2001).

Emodin-8-*O*-glucoside (PO2) (1,3,8-trihydroxy-6-methylanthraquinone ; 8-*O*-β-glucopyranoside. Yellow amorphous powder. Pure in M31. UV max: 221; 281; 425 nm. ^{1}H NMR (DMSO-d_6, 500 MHz) δ 2.40 (3H, s, 3Me), 3.24 (1H, td, *J*=9.3, 3.9 Hz, H-4'), 3.41 (2H, m, H-2', H-5'), 3.52 (1H, dt, *J*=11.3, 5.0 Hz, H-6'b), 3.72 (1H, bd, *J*=11.3 Hz, H-6'a), 4.59 (1H, t, *J*=5.0 Hz, 6'OH), 5.05 (1H, d, *J*=7.4 Hz, H-1'), 5.05 (2H, overlapped, 2'OH, 4'OH), 5.09 (1H, d, *J*=5.0 Hz, 3'OH), 7.00 (1H, d, *J*=2.4 Hz, H-7), 7.15 (1H, d, *J*=1.7 Hz, H-2), 7.28 (1H, d, *J*=2.4 Hz, H-5), 7.46 (1H, d, *J*=1.7 Hz, H-4), 13.16 (1H, s, 1OH); ^{13}C NMR (DMSO-d_6, 126 MHz) δ 21.4 (3Me), 60.6 (C-6'), 69.5 (C-4'), 73.3 (C-2'), 76.4 (C-3'), 77.3 (C-5'), 100.8 (C-1'), 108.3 (C-5, C-7), 113.3 (C-8a), 114.4 (C-9a), 119.2 (C-4), 124.1 (C-2), 132.1 (C-4a), 136.5 (C-10a), 146.9 (C-3), 161.0 (C-8), 161.7 (C-1), 164.2 (C-6), 182.1 (C-10), 186.4 (C-9). ESI-HRMS : *m/z* 431.0990 [M-H]$^-$ (calculated for C21H19O10, *m/z* 431.0978, 1.5 ppm) (Demirezer et al., 2001).

Baicalin (SC1) (5,6,7-trihydroxyflavone ;7-*O*-β-D-glucuronopyranoside). Amorphous powder. Major in M12-13, pure in M20S3. UV max: 220; 276; 318 nm. ^{1}H NMR (DMSO-d_6, 500 MHz) δ 3.38 (3H, m, H-2'', H-3'', H-4''), 3.97 (1H, d, *J*=9.5 Hz, H-5''), 5.19 (1H, d, *J*=7.5 Hz, H-1''), 7.00 (1H, s, H-3), 7.04 (1H, s, H-8), 7.60 (3H, m, H-3', H-4', H-5'), 8.08 (2H, d, *J*=7.3 Hz, H-2', H-6'), 8.64 (1H, s, 6OH), 12.57 (1H, s, 5OH); ^{13}C NMR (DMSO-d_6, 126 MHz) δ 182.4 (C-4), 170.1 (C-6''), 163.5 (C-2), 151.3 (C-7), 149.1 (C-9), 146.6 (C-5), 131.8 (C-4'), 130.6 (C-6), 128.9 (C1', C-3', C-5'), 126.1 (C-2', C-6'), 106.0 (C-10), 105.1 (C-3), 100.0 (C-1''), 93.7 (C-8), 75.2 (C-3''), 75.0 (C-5''), 72.6 (C-2''), 71.2 (C-4''). ESI-HRMS *m/z* 445.0782 [M-H]$^-$ (calculated for $C_{21}H_{17}O_{11}$, *m/z* 445.0771, 1.3 ppm) (Wu et al., 2005) .

Wogonoside (SC2) (5,7-dihydroxy-8-methoxyflavone ; 7-*O*-β-D-glucuronopyranoside). Amorphous powder. Pure in M25. UV max: 220; 276; 355 nm. ^{1}H NMR (DMSO-d_6, 500 MHz) δ 3.39 (3H, m, H-2'', H-3'', H-4''), 3.89 (3H, s, 8OMe), 4.01 (1H, d, *J*=9.7 Hz, H-5''), 5.27 (1H, d, *J*=7.0 Hz, H-1''), 6.70 (1H, s, H-6), 7.05 (1H, s,

H-3), 7.61 (3H, m, H-3', H-4', H-5'), 8.08 (2H, d, *J*=7.6 Hz, H-2', H-6'), 12.52 (1H, s, 5OH); ^{13}C NMR (DMSO-d_6, 126 MHz) δ 61.4 (C-8OMe), 71.2 (C-4''), 72.9 (C-2''), 75.3 (C-5''), 75.8 (C-3''), 98.7 (C-6), 99.7 (C-1''), 105.2 (C-3), 105.4 (C-10), 126.4 (C-2', C-6'), 129.3 (C-3', C-5'), 129.3 (C-8), 130.7 (C-1'), 132.2 (C-4'), 149.2 (C-9), 156.0 (C-5, C-7), 163.6 (C-2), 170.0 (C-6''), 182.3 (C-4). ESI-HRMS *m/z* 459.0941 [M-H]$^-$ (calculated for $C_{22}H_{19}O_{11}$, *m/z* 459.0927, 1.8 ppm) (Wu et al., 2005).

Oroxyloside (SC3) (5, 7-dihydroxy-6-methoxyflavone; 7-*O*-β-D-glucuronopyranoside). Amorphous powder. Pure in M23S17. UV max: 214; 271; 310 nm. ^{1}H NMR (CD_3OD, 600 MHz) δ 3.56 (1H, t, *J*=9.1 Hz, H-3''), 3.62 (2H, t, *J*=9.1 Hz, H-2'', H-4''), 3.91 (3H, s, OCH_3), 4.02 (1H, d, *J*=9.8 Hz, 5''), 5.21 (1H, d, *J*=7.5 Hz, H-1''), 6.82 (1H, s, H-3), 7.03 (1H, s, H-8), 7.58 (3H, m, H-3', H-4', H-5'), 8.04 (2H, d, *J*=7.0 Hz, H-2', H-6'); ^{13}C NMR (CD_3OD, 151 MHz) δ 61.5 (C-OCH_3), 73.1 (C-4''), 74.5 (C-2''), 76.6 (C-5''), 77.5 (C-3''), 96.0 (C-8), 101.9 (C-1''), 105.9 (C-3), 107.9 (C-6), 127.7 (C-2', C-6'), 130.3 (C-3', C-5'), 132.5 (C-10), 133.2 (C-4'), 134.4 (C-1'), 154.2 (C-5), 154.5 (C-9), 158.1 (C-7), 166.4 (C-2), 174.0 (6''), 184.5 (C-4). ESI-HRMS *m/z* 459.0942 [M-H]$^-$ (calculated for $C_{22}H_{19}O_{11}$, *m/z* 459.0927, 2 ppm) (Abe et al., 1990).

Astilbin (SM1), (3, 3', 4', 5, 7-pentahydroxyfavanone; (2R, 3R)-form, 3-*O*-α-L-rhamnopyranoside). Impur in M17. UV max: 200; 220; 290 nm. ^{1}H NMR (MeOD, 600 MHz) δ 1.19 (3H, d, *J*=6.2 Hz, H-6''), 3.30 (1H, overlapped, H-4''), 3.54 (1H, dd, *J*=3.3, 1.7 Hz, H-2''), 3.66 (2H, dd, *J*=9.6, 3.3 Hz, H-3''), 4.05 (1H, d, *J*=1.7 Hz, H-1''), 4.25 (1H, dq, *J*=9.6, 6.2 Hz, H-5''), 4.58 (1H, d, *J*=10.7 Hz, H-3), 5.07 (1H, d, *J*=10.7 Hz, H-2), 5.90 (1H, d, *J*=2.2 Hz, H-8), 5.92 (1H, d, *J*=2.2 Hz, H-6), 6.80 (3H, d, *J*=8.2 Hz, H-5'), 6.84 (1H, dd, *J*=8.2, 2.0 Hz, H-6'), 6.96 (1H, d, *J*=2.0 Hz, H-2'); ^{13}C NMR (MeOD, 151 MHz) δ 17.9 (C-6''), 70.5 (C-5''), 71.8 (C-2''), 72.2 (C-3''), 73.8 (C-4''), 78.6 (C-3), 84.0 (C-2), 102.2 (C-1''), 102.5 (C-10), 115.5 (C-2'), 116.3 (C-5'), 120.5 (C-6'), 129.2 (C-1'), 146.5 (C-3'), 147.4 (C-4'), 164.1 (C-9), 165.5 (C-5), 168.6 (C-7), 196.0 (C-4). ESI-HRMS: *m/z* 449.1088 [M-H]$^-$ (calculated for $C_{21}H_{21}O_{11}$, *m/z* 449.1083, -0.2 ppm) (Galotta et al., 2008).

Oxymatrine (SO1) (matrine; N^1-Oxide). Amorphous powder. Pure in M14S6. ^{1}H NMR (DMSO-d_6, 600 MHz) δ 1.24 (1H, tdd, *J*=12.7, 9.6, 3.1 Hz, H-12''), 1.53 (1H, m H-13''), 1.56 (2H, m, H-3'', H-9''), 1.58 (1H, m, H-8''), 1.64 (2H, m, H-4'', H-7), 1.71 (2H, m, H-4', H-13'), 1.79 (1H, m, H-5), 1.94 (1H, d, *J*=13.8 Hz, H-8'), 2.15 (2H, m, H-12', H-14''), 2.24 (2H, m, H-9', H-14'), 2.32 (1H, m, H-3'), 3.37 (4H, m, H-2, H-10), 3.45 (1H, bs, H-6), 3.59 (1H, q, *J*=11.4 Hz, H-17''), 4.23 (1H, dd, *J*=12.4, 5.3 Hz, H-17'), 4.49 (1H, bs, H-11); ^{13}C NMR (DMSO-d_6, 151 MHz) δ 16.6 (C-9), 16.6 (C-3), 18.5 (C-13), 23.3 (C-8), 24.9 (C-4), 27.8 (C-12), 32.6 (C-14), 33.3 (C-5), 41.0 (C-17), 41.1 (C-7), 52.9 (C-11), 66.2 (C-2), 66.4 (C-6), 66.6 (C-10), 168.8 (C-15). ESI-HRMS: *m/z* 265.1918 [M+H]$^+$ (calculated for $C_{15}H_{25}N_2O_2$, *m/z* 265.1916, 2.7 ppm) (Aslanov et al., 1987)

8 Multi-component signatures

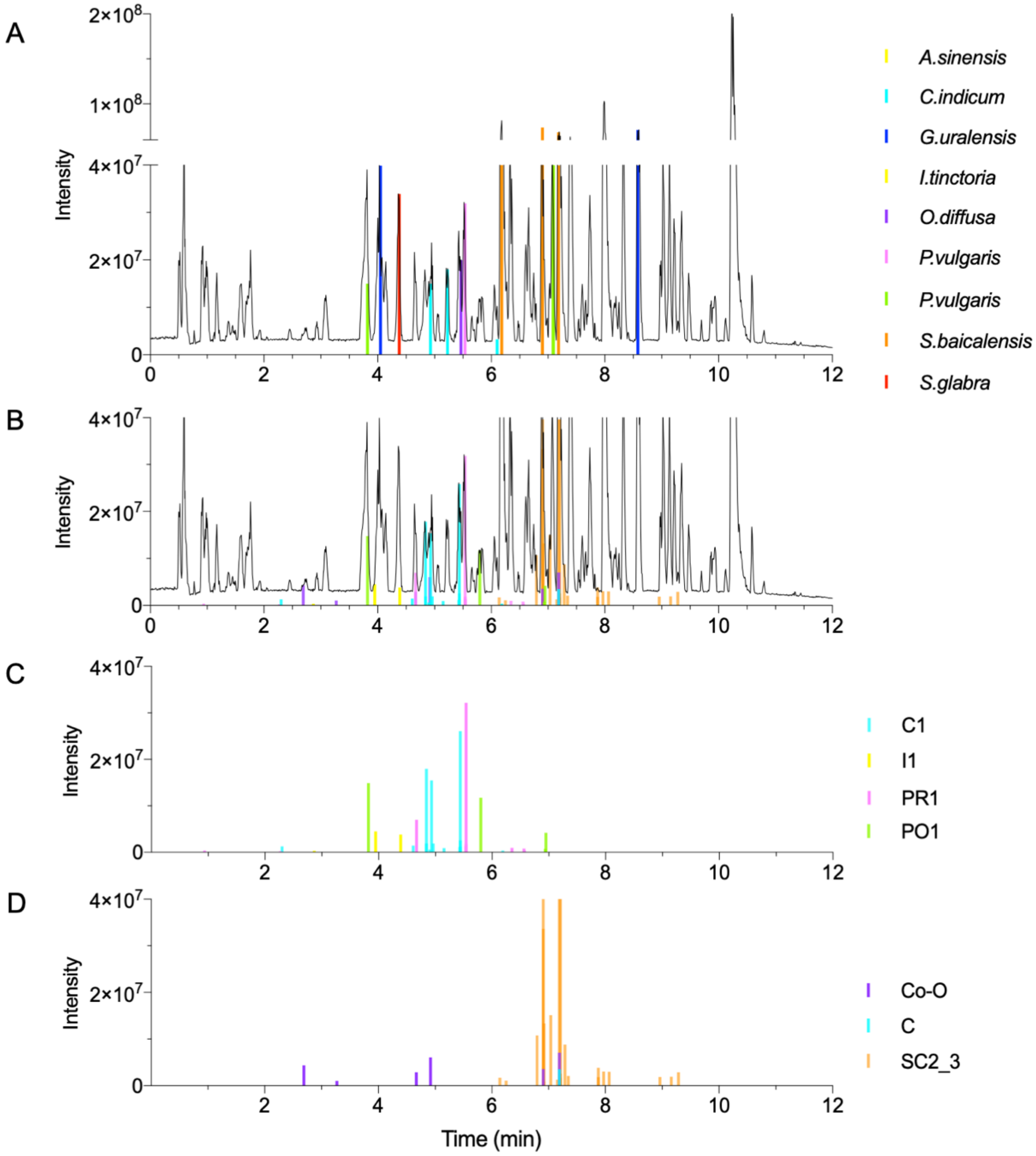

Supplementary Figure 15: multi-component signatures in NI*:* A) metabolite profile of the formula showing all formally identified components. B) metabolite profile of the formula showing some of the selected multi-component signatures. C) multicomponent signatures for *I. tinctoria* and its marker isovitexin (I1), *P. cuspidatum* and *E*-piceid (PO1), *P.vulgaris* and rosmarinic acid (PR1) and *C.indicum* and C1. D) C)

multicomponent signatures for *S.baicalensis* and wogonoside and oroxyloside (SC2 and SC3), and for *O.diffusa.* See table S3 to S12 for annotations.

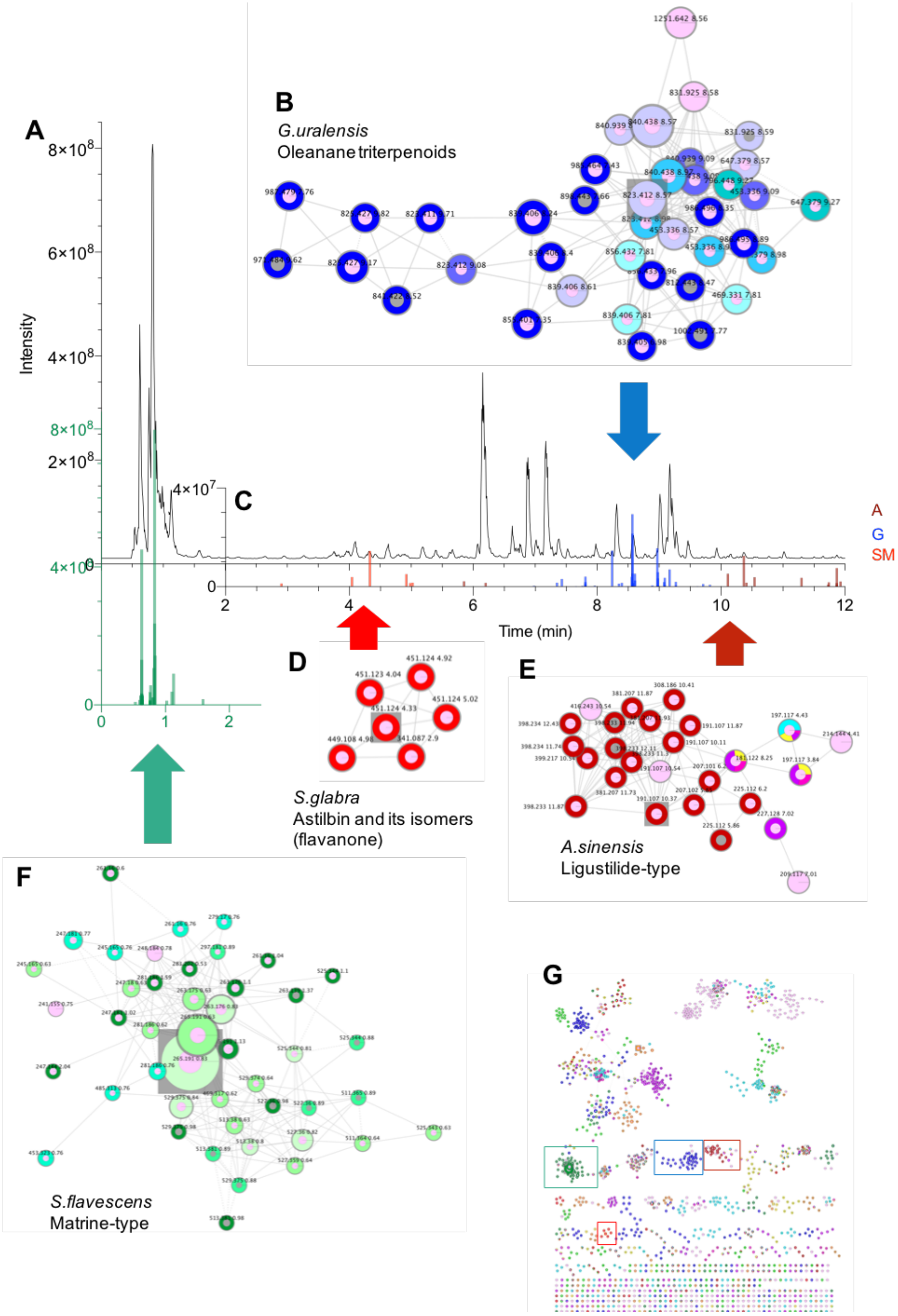

Supplementary Figure 16: representation of clusters from the FBMN-PI in relation to the chromatogram of the formula: A) UHPLC-HRMS metabolite profile of the formula in PI, B) cluster containing potential co-markers for *G.uralensis*, the color tones of the nodes indicate the features with the same retention time, C) bar plot showing the peak height intensity of related clusters, D) cluster with potential co-markers for *S.glabra*, E)

cluster with potential co-markers for *A.sinensis*, F) cluster with potential co-markers for *S.flavescens,* the color tones of the nodes indicate the features with the same retention time. The numbers indicate the *m/z* ratio of the nodes, followed by their retention time See table S3, S5, S11 and S12 for related annotation. G) FBMN. See Fig.4 for the node layout legends.

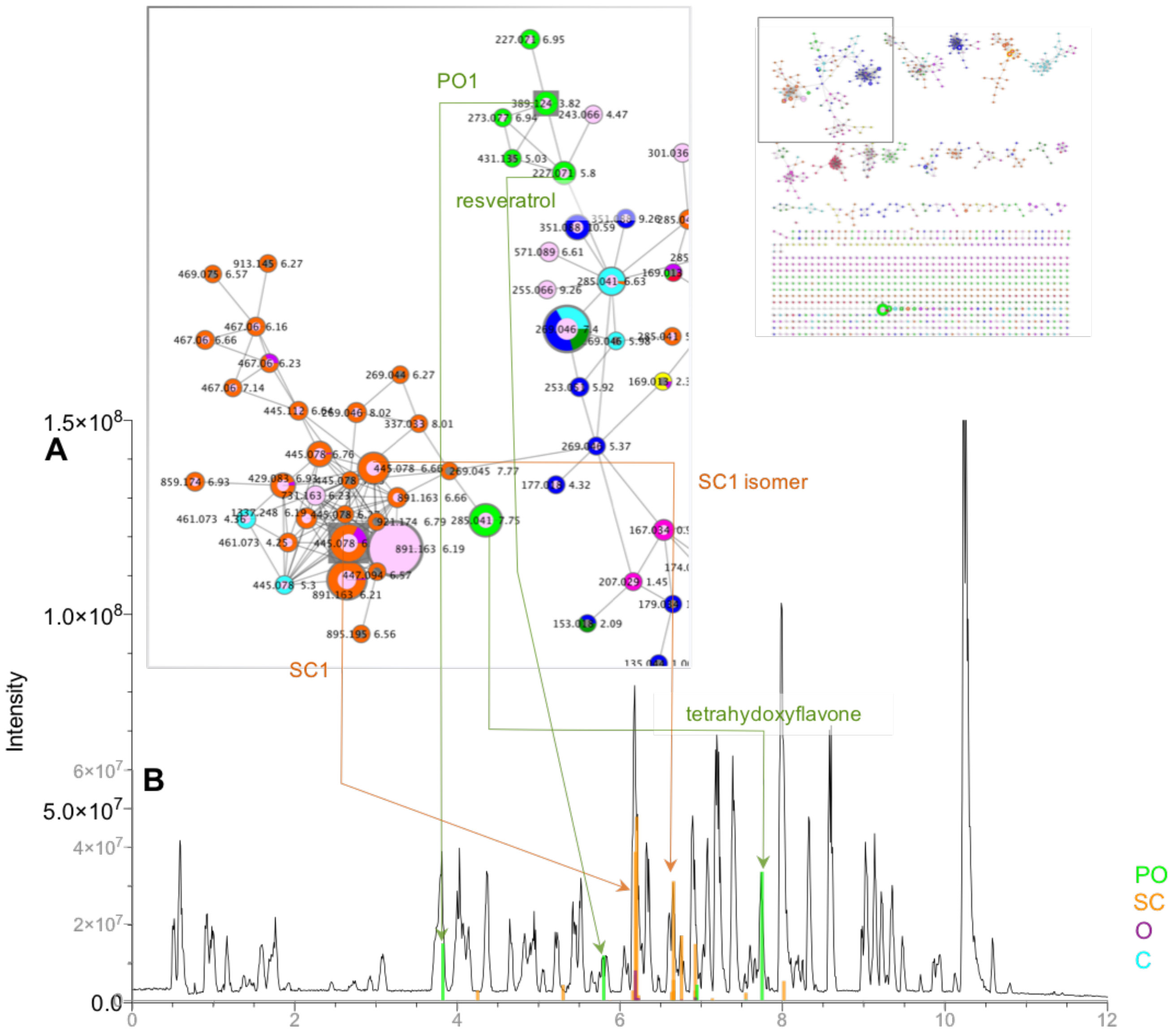

Supplementary Figure 17: representation of a cluster from the FBMN-NI in relation to the chromatogram of the formula: A) UHPLC-HRMS metabolite profile of the formula in NI, B) bar plot showing the peak height intensity of related cluster, which contains potential co-markers for *S.baicalensis* and *P.cuspidatum*. The numbers indicate the *m/z* ratio of the nodes, followed by their retention time. See table S8 and S10 for related annotation. See Fig.4D for the node layout legends.

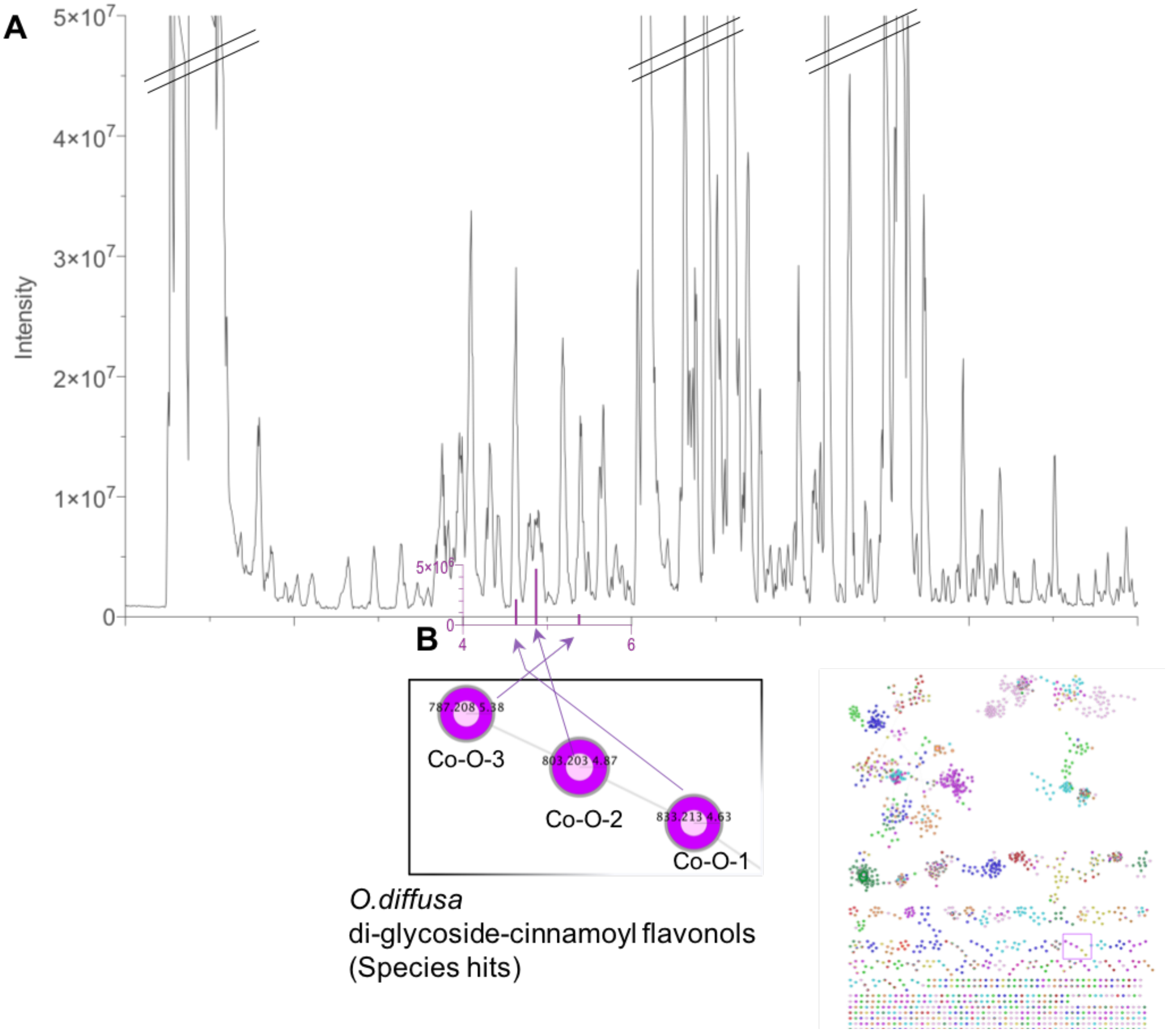

Supplementary Figure 18: representation of a cluster from the FBMN-PI in relation to the chromatogram of the formula: A) UHPLC-HRMS/MS metabolite profile of the formula in PI, B) cluster with potential co-markers for *O.diffusa* and the corresponding histogram of the node peak heights as a function of time. The numbers indicate the *m/z* ratio of the nodes, followed by their retention time. See table S7 for related annotation. See Fig. 4D for the node layout legends.

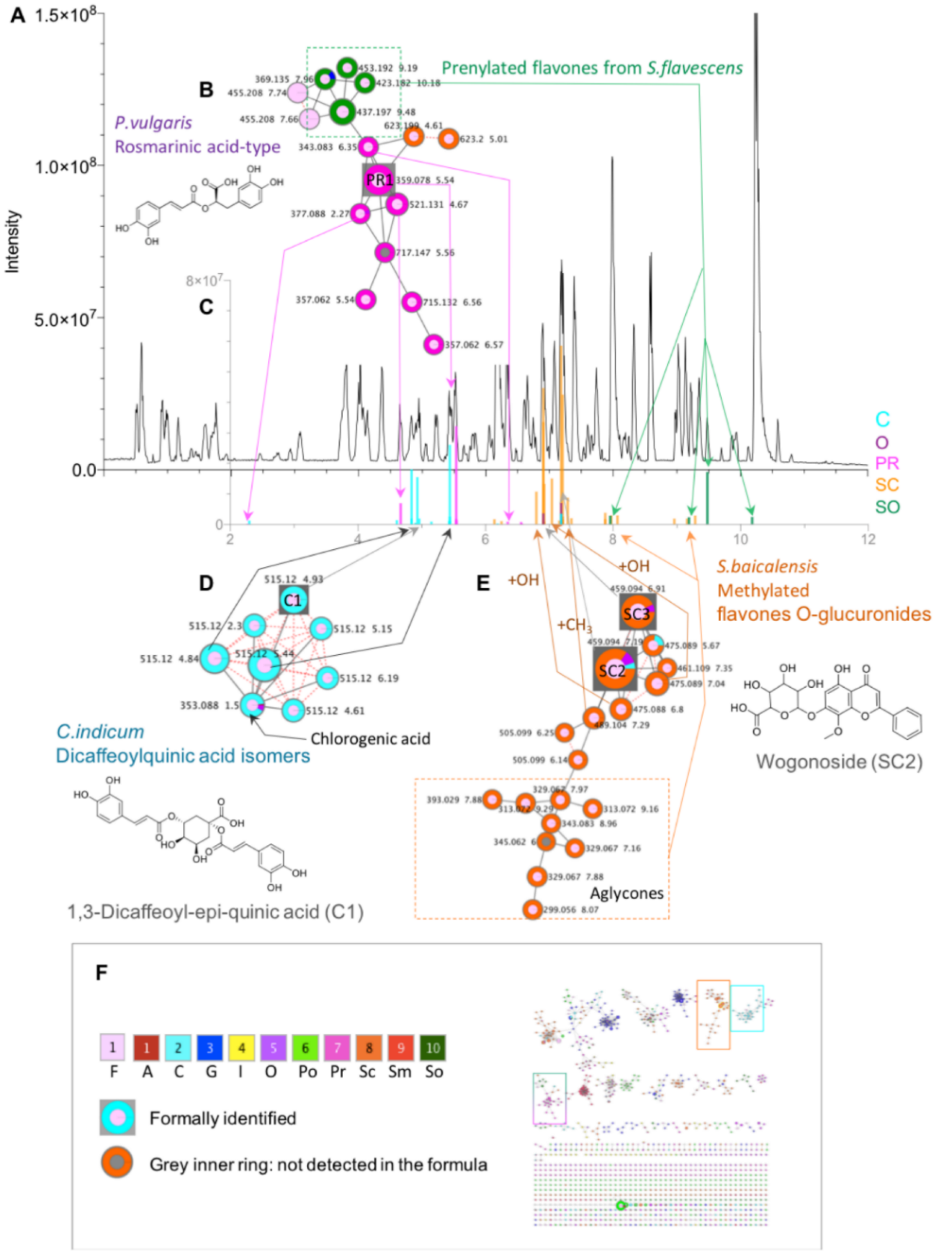

Supplementary Figure 19: representation of a cluster from the FBMN in NI in relation to the chromatogram of the formula: A) UHPLC-HRMS/MS metabolite profile of the formula in NI, B) cluster with potential co-markers for *P.vulgaris and S. flavescens*, C) bar plot showing the peak heights of related nodes in the cluster, D) cluster with potential co-markers for *C. indicum,* E) cluster with potential co-markers for *S.baicalensis*. The

numbers on the nodes indicate the *m/z* ratio of the nodes, followed by their retention time. See table S4, S8, S8 and S9 for related annotation. See Fig. 4D for the node layout legends.

9 UHPLC-UV-PDA HRMS/MS data acquisition

The analyses were performed on an Acquity UPLC system interfaced to an Orbitrap Q-Exactive Focus mass spectrometer (Thermo Scientific) using a heated electrospray ionization (HESI-II) source and an Acquity UPLC PDA detector. Thermo Scientific Xcalibur 2.1 software was employed for instrument control. PDA detector recorded from 210 to 450 nm (resolution 1.2 nm). The optimized HESI-II parameters were as follows: source voltage, 3.5 kV (PI), 2.5 kV(NI); sheath gas flow rate (N2), 47.5 units; auxiliary gas flow rate, 11.25 units; spare gas flow rate, 2.25; capillary temperature, 256.25°C, S-Lens RF Level, 45. The mass analyzer was calibrated using a mixture of caffeine, methionine–arginine–phenylalanine– alanine–acetate (MRFA), sodium dodecyl sulfate, sodium taurocholate, and Ultramark 1621 in an acetonitrile/methanol/ water solution containing 1% formic acid by direct injection. The data-dependent MS/MS events were performed on the three most intense ions detected in full scan MS (Top3 experiment). The MS/MS isolation window width was 1 Da, and the stepped normalized collision energy (NCE) was set to 15, 30 and 45 units. In data-dependent MS/MS experiments, full scans were acquired at a resolution of 35,000 FWHM (at m/z 200) and MS/MS scans at 17,500 FWHM both with an automatically determined maximum injection time. After being acquired in a MS/MS scan, parent ions were placed in a dynamic exclusion list for 2.0 s

10 MZmine parameters for peak picking

Supplementary Table S3: MZmine parameters

<table>
<tr><td colspan="2" rowspan="2">MZmine Parameters</td><td colspan="2">HRMS/MS</td><td>Fraction control</td></tr>
<tr><td>PI</td><td>NI</td><td>NI</td></tr>
<tr><td>1</td><td colspan="4">Mass detection</td></tr>
<tr><td></td><td>Noise level MS1</td><td>1E5</td><td>1E5</td><td>1E2</td></tr>
<tr><td></td><td>Noise level MS2</td><td>1</td><td>1</td><td>NA</td></tr>
<tr><td>2</td><td colspan="4">Peak detection -> ADAP chromatogram builder</td></tr>
<tr><td></td><td>Min group size in # of scans</td><td>5</td><td>5</td><td>5</td></tr>
<tr><td></td><td>Group intensity threshold</td><td>1E5</td><td>5E4</td><td>1E2</td></tr>
<tr><td></td><td>Min highest intensity</td><td>5E5</td><td>2E5</td><td>2E2</td></tr>
<tr><td></td><td>m/z tolerance [ppm]</td><td>5</td><td>5</td><td>20</td></tr>
<tr><td>3</td><td colspan="4">Peak detection -> chromatogram deconvolution Wavelets (ADAP)</td></tr>
<tr><td></td><td>n/z center calculation</td><td colspan="3">MEDIAN</td></tr>
<tr><td></td><td>S/N threshold</td><td colspan="3">10</td></tr>
<tr><td></td><td>S/N estimator:</td><td colspan="3">intensity window SN</td></tr>
<tr><td></td><td>Min feature height</td><td>1E5</td><td>1E5</td><td>2E2</td></tr>
<tr><td></td><td>Coefficient area threshold</td><td colspan="3">100</td></tr>
<tr><td></td><td>Peak duration range [min]</td><td colspan="3">0.00- 1.50</td></tr>
<tr><td></td><td>RT wavelet range [min]</td><td colspan="3">0.00 – 0.06</td></tr>
<tr><td></td><td>m/z range for MS2 scan pairing [Da]</td><td colspan="2">0.025</td><td>NA</td></tr>
<tr><td></td><td>RT range for MS2 scan pairing [min]</td><td colspan="2">0.08</td><td>NA</td></tr>
<tr><td>4</td><td colspan="4">Isotopic peaks grouper</td></tr>
<tr><td></td><td>m/z tolerance [ppm]</td><td colspan="2">5</td><td>10</td></tr>
<tr><td></td><td>Retention time tolerance absolute [min]</td><td colspan="3">0.01</td></tr>
<tr><td></td><td>Maximum charge</td><td colspan="3">2</td></tr>
<tr><td></td><td>Representative isotope</td><td colspan="3">Most intense</td></tr>
<tr><td>5</td><td colspan="4">Filtering -> Duplicate peak filter</td></tr>
<tr><td></td><td>m/z tolerance [ppm]</td><td colspan="3">1</td></tr>
<tr><td></td><td>Retention time tolerance absolute [min]</td><td colspan="3">0.01</td></tr>
<tr><td>6</td><td colspan="4">Alignment -> JOIN</td></tr>
<tr><td></td><td>m/z tolerance [ppm]</td><td colspan="3">5</td></tr>
<tr><td></td><td>Weight for m/z [Da]</td><td colspan="3">1</td></tr>
<tr><td></td><td>Retention time tolerance absolute [min]</td><td colspan="3">0.02</td></tr>
</table>

	Weight for RT	1	
	Require same charge state	☑	
	Compare isotope pattern ->	5 ppm<br>1E3<br>Min score 70%	20 ppm<br>1E2<br>Min score 70%
9	Identification -> Custom Database, adduct search, complex search		
	Retention time tolerance absolute [min]	0.01	0.05
	m/z tolerance [ppm]	5	20
	Max complex/adduct peak height	10000%	
11	Filtering -> peak list rows filter		
	Keep only peaks with MS2 scan (GNPS)	☑	NA
	Reset the peak number ID		
	Export to .csv		
	Export to .mgf for GNPS		

11 References

Abe, K., Inoue, O., and Yumioka, E. (1990). Biliary excretion of metabolites of baicalin and baicalein in rats. *Chem. Pharm. Bull.* 38(1)**,** 208-211. doi: 10.1248/cpb.38.208.

Aslanov, K.A., Kushmuradov, Y.K., and Sadykov, A.S. (1987). Lupine alkaloids. *Alkaloids (Academic Press)* 31**,** 117-192.

Baltina, L.A., Kunert, O., Fatykhov, A.A., Kondratenko, R.M., Spirikhin, L.V., Baltina, L.A., Jr., et al. (2005). High-Resolution 1H and 13C NMR of Glycyrrhizic Acid and Its Esters. *Chem. Nat. Compd.* 41(4)**,** 432-435. doi: 10.1007/s10600-005-0171-2.

Chen, L., Han, Y., Yang, F., and Zhang, T. (2001). High-speed counter-current chromatography separation and purification of resveratrol and piceid from Polygonum cuspidatum. *J. Chromatogr. A* 907(1-2)**,** 343-346. doi: 10.1016/s0021-9673(00)00960-2.

Demirezer, L.O., Kuruuzum-Uz, A., Bergere, I., Schiewe, H.J., and Zeeck, A. (2001). The structures of antioxidant and cytotoxic agents from natural source: anthraquinones and tannins from roots of Rumex patientia. *Phytochemistry* 58(8)**,** 1213-1217. doi: 10.1016/s0031-9422(01)00337-5.

DNP (2019a). Dictionary of Natural Products on DVD (27.2), CRC Press, Taylor & Francis Group.

DNP (2019b). *Dictionary of Natural Products online, CRC Press, Taylor & Francis Group* [Online]. Available: http://dnp.chemnetbase.com/faces/chemical/ChemicalSearch.xhtml;jsessionid=799431583FE9D168C0362E77D720728D [Accessed 17.09.2019].

Galotta, A.L.Q.A., Boaventura, M.A.D., and Lima, L.A.R.S. (2008). Antioxidant and cytotoxic activities of 'acai' (Euterpe precatoria Mart.). *Quim. Nova* 31(6)**,** 1427-1430. doi: 10.1590/s0100-40422008000600028.

Kim, H.J., and Lee, Y.S. (2005). Identification of new dicaffeoylquinic acids from Chrysanthemum morifolium and their antioxidant activities. *Planta Med.* 71(9)**,** 871-876. doi: 10.1055/s-2005-873115.

Kitagawa, I., Chen, W.Z., Hori, K., Harada, E., Yasuda, N., Yoshikawa, M., et al. (1994). Chemical Studies of Chinese Licorice-Roots. I. Elucidation of five new flavonoid constituents from the roots of Glycyrrhiza glabra L. collected in Xinjiang. *Chem. Pharm. Bull.* 42(5)**,** 1056-1062. doi: 10.1248/cpb.42.1056.

Otsuka, H., Yoshimura, K., Yamasaki, K., and Cantoria, M.C. (1991). Isolation of 10-acyl iridoid glucosides from a Philippine medicinal plant, Oldenlandia corymbosa L. (Rubiaceae). *Chem. Pharm. Bull.* 39(8)**,** 2049-2052. doi: 10.1248/cpb.39.2049.

Patiny, L., and Borel, A. (2013). ChemCalc: A Building Block for Tomorrow's Chemical Infrastructure. *Journal of Chemical Information and Modeling* 53(5)**,** 1223-1228. doi: 10.1021/ci300563h.

Redaelli, C., Formentini, L., and Santaniello, E. (1980). Apigenin 7-glucoside and its 2″- and 6″-acetates from ligulate flowers of Matricaria chamomilla. *Phytochemistry* 19(5)**,** 985-986. doi: doi.org/10.1016/0031-9422(80)85160-0.

Sumner, L.W., Amberg, A., Barrett, D., Beale, M.H., Beger, R., Daykin, C.A., et al. (2007). Proposed minimum reporting standards for chemical analysis. Chemical Analysis Working Group (CAWG) Metabolomics Standards Initiative (MSI). *Metabolomics* 3(3)**,** 211-221. doi: 10.1007/s11306-007-0082-2.

Svehlikova, V., Bennett, R.N., Mellon, F.A., Needs, P.W., Piacente, S., Kroon, P.A., et al. (2004). Isolation, identification and stability of acylated derivatives of apigenin 7-O-glucoside from chamomile (Chamomilla recutita [L.] Rauschert). *Phytochemistry* 65(16)**,** 2323-2332. doi: 10.1016/j.phytochem.2004.07.011.

Wu, S., Sun, A., and Liu, R. (2005). Separation and purification of baicalin and wogonoside from the Chinese medicinal plant Scutellaria baicalensis Georgi by high-speed counter-current chromatography. *J. Chromatogr. A* 1066(1-2)**,** 243-247. doi: 10.1016/j.chroma.2005.01.054.
